# Development of a predictive risk model for school readiness at age 3 years using the UK Millennium Cohort Study

**DOI:** 10.1136/bmjopen-2018-024851

**Published:** 2019-06-17

**Authors:** Christine Camacho, Viviane S Straatmann, Jennie C Day, David Taylor-Robinson

**Affiliations:** 1 Department of Public Health and Policy, University of Liverpool, Liverpool, UK; 2 Aging Research Centre, Karolinska Institute, Stockholm, Sweden

**Keywords:** public health, epidemiology, social medicine

## Abstract

**Objectives:**

The aim of this study is to develop a predictive risk model (PRM) for school readiness measured at age 3 years using perinatal and early infancy data.

**Design and participants:**

This paper describes the development of a PRM. Predictors were identified from the UK Millennium Cohort Study wave 1 data, collected when participants were 9 months old. The outcome was school readiness at age 3 years, measured by the Bracken School Readiness Assessment. Stepwise selection and dominance analysis were used to specify two models. The models were compared by the area under the receiver operating characteristic curve (AUROC) and integrated discrimination improvement (IDI).

**Results:**

Data were available for 9487 complete cases. At age 3, 11.7% (95% CI 11.0% to 12.3%) of children were not school ready. The variables identified were: parents’ Socio-Economic Classification, child’s ethnicity, maternal education, income band, sex, household number of children, mother’s age, low birth weight, mother’s mental health, infant developmental milestones, breastfeeding, parents’ employment, housing type. A parsimonious model included the first six listed variables (model 2). The AUROC for model 1 was 0.80 (95% CI 0.78 to 0.81) and 0.78 (95% CI 0.77 to 0.79) for model 2. Model 1 resulted in a small improvement in discrimination (IDI=1.3%, p<0.001).

**Conclusions:**

Perinatal and infant risk factors predicted school readiness at age three with good discrimination. Social determinants were strong predictors of school readiness. This study demonstrates that school readiness can be predicted by six attributes collected around the time of birth.

**Strengths and limitations of this study**

- Use of a large, representative and contemporary cohort study to demonstrate the feasibility of predicting school readiness from data collected in infancy.
- Multiple imputation and bootstrapping were used to evaluate the impact of missing data and internal validity, respectively.
- The main outcome measure, the Bracken School Readiness Assessment, was developed in the USA and is not routinely used in the UK.
- This model was not externally validated, which would have given an indication of generalisability.

## Introduction

Early childhood is a critical time for lifelong physical, social, emotional and cognitive development. A wide range of factors are associated with early cognitive development (ECD).^1^ Interventions in the first 3 years of life can improve the trajectory of ECD^2^ and deliver the greatest return on investment,^3^ yet it is unclear how best to identify children at most risk of delayed ECD, to enable appropriate targeting of interventions.

Cognitive development measures in children are good indicators of later educational achievement, predict health and social care needs in adults,^4 5^ and are associated with long term health outcomes.^6^ There has been a growing policy interest in school readiness as a measure of ECD,^7^ and school readiness is a key public health indicator in children in the UK. Good school readiness lays a platform for future learning, employment and health.^8 9^

School readiness is currently a major focus in England for policy makers, educators and the public health community^10^ and national metrics are collected to capture changes over time. In 2017, 29% of children in England were deemed not school ready at the end of their reception year (aged 4–5 years).^11^ The percentage of children school ready was nearly 20% higher in the most affluent decile (80% school ready) compared with the most deprived decile (62% school ready) when areas were classified into deciles according to the Index for Multiple Deprivation.^12^ In UK policy there has been a focus on demographic factors e.g. maternal age, in targeting early interventions for children.^13^ This study will explore the importance of different variables in predicting school readiness.

Previous research has identified a wide range of variables associated with ECD. Predictive risk models (PRMs) are well-established in many clinical disciplines and have more recently been applied to child development. Using PRMs in this context could facilitate targeted early intervention as part of a proportionate universalism approach, which requires universal action with the scale and intensity of interventions proportionate to the level of need.^6^ Most models thus far have shown fair or poor discrimination and there have been very few studies in the UK.^14–18^ The aim of this study was to develop, for the first time, a PRM for school readiness measured at age 3 years using perinatal and early infancy data from the UK Millennium Cohort Study (MCS).

## Methods

### Overview

Data from the MCS were used to explore the relationship between the outcome, school readiness and 29 predictor variables using logistic regression analysis. Following univariable analysis to test for unadjusted associations, automated stepwise regression analyses were used to select variables for inclusion in the PRM. Dominance analysis was used to rank and weight included predictors, and integrated discrimination improvement (IDI) was calculated to assess the difference in performance between models. A receiver operator characteristic (ROC) curve was used to evaluate how well the model discriminated school readiness. The area under an ROC curve (AUROC) gives a measure of how well the regression model predicts school readiness at age 3. Traditionally accepted AUROC cut-off points are: 0.9–1=excellent, 0.8–<0.9= good, 0.7–<0.8= fair, 0.6–<0.7=poor, 0.5–<0.6=fail.^19^ Multiple imputation was used to assess the impact of missing data in the sample.

### Data source

The PRM was developed and validated using MCS data. The MCS is a nationally representative birth cohort study which recruited 18 550 children born from September 2000 to January 2002, followed up in ongoing data collection waves. The sampling frame was government child benefit records, which had almost universal coverage at the time of sampling. The sample was clustered at the level of electoral ward and stratified to allow over representation of children living in deprived areas and areas with high concentrations of ethnic minorities.^20^ Further information about the MCS sample is available in the cohort profile.^21^ Data were collected from the main responder (usually mothers) by trained interviewers in participants’ homes using a combination of interviews and self-completed questions. All singleton children in the first (aged 9 months) and second (aged 3 years) waves of the MCS with completed data for the outcome and predictors were eligible for inclusion (n=9487).

### Outcome

School readiness was measured using the Bracken School Readiness Assessment (BSRA) which consists of 6 subtests relating to colours, letters, numbers/counting, sizes, comparisons and shapes.^20^ The assessment was carried out by interviewers during the second data collection wave when children were aged approximately 3 years old. The BSRA and its predecessors have demonstrated good reliability^22^ and validity against other measures and teacher assessments.^23^

The BSRA raw scores were summed and adjusted for age to provide a standardised composite score.^20^ Scores were grouped according to cut-offs recommended by Bracken which reflected a ‘normative classification’ whereby children were categorised as very delayed, delayed, average, advanced or very advanced.^24^ We used the same cut-off score as Bracken (mean standardised composite score <85, 1 SD below mean) but collapsed the categories of delayed or very delayed into a single category equivalent to not being school ready. We have dichotomised the outcome ‘school readiness’ in line with UK policy, and to allow the testing of a PRM using ROC analysis which requires a binary outcome.^25^

### Predictors

**Figure 1:**
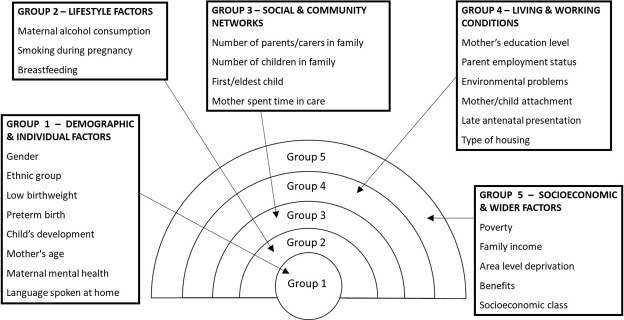
Rainbow Model showing determinants of school readiness (adapted from Dahlgren and Whitehead^34^).

Twenty-nine predictor variables were used, which were collected at age 9 months in the first wave of MCS data collection during which data relevant to pregnancy, birth and the perinatal period was captured retrospectively. These were identified from previous research to predict cognitive development and were included in the MCS.^1 2 4 6 26–33^ The selected predictor variables were grouped according to the Dahlgren and Whitehead theoretical model^34^ of social determinants of health as depicted in figure 1. This model was chosen to provide a framework for categorising predictors to allow analysis of the determinants of ECD.

#### Group 1: demographic and individual factors

Demographic characteristics included child sex, maternal ethnicity, child weight, pre-term birth, mother’s age, home language, maternal mental health and child development categorised as shown in box 1.

Box 1 **Coding of group 1 demographic and individual factors**

Categorisation of demographic and individual factors

Child sex: ‘female’ and ‘male’.

Maternal ethnicity: ‘white’, ‘mixed’, ‘Indian’, ‘Pakistani and Bangladeshi’, ‘Black’ and ‘other’.

Child weight at birth: low (<2.5 kg) or normal/high (≥2.5 kg).

Preterm birth: gestation period less than 37 weeks.

Mother’s age in years at birth of first child: grouped into four categories (14–19, 20–29, 30–39, 40+years).

Home language: ‘English only’, ‘English and another language’, ‘another language only’.

Mental health (1): sad or low for>2 weeks since baby, coded as ‘yes’ or ‘no’.

Mental health (2): diagnosis of depression or serious anxiety, coded as ‘yes’ or ‘no’.

Mental health (3): 9-item modified version of the Rutter Malaise Inventory,^39^ coded as ‘low’ or (0–3) ‘high’ (4-9) scores.^27^

Child development: – eight items from Denver Developmental Screening Test and five items from MacArthur Communicative Development Inventory, scored on a continuous scale from 13 (above average) to 36 (below average).

#### Group 2: lifestyle factors

Self-reported maternal smoking was coded as ‘never smoked’, ‘smoked before pregnancy’ and ‘smoked during pregnancy’. Maternal alcohol consumption during pregnancy were categorised as ‘never or very infrequent’, ‘occasional’, ‘regularly’ and ‘most or everyday’. Breastfeeding duration was grouped as ‘never’, ‘1 week or less’, ‘1–6 weeks’, ‘6 weeks – 6 months’ and ‘over 6 months’.

#### Group 3: social and community networks

The number of children in household was coded as ‘1’, ‘2–3’ or ‘4+’, and being the eldest or only child was recoded as ‘yes’ or ‘no’. The number of parents or carers was either ‘1’ or ‘2’. Mothers were asked how much time they had spent time in care before the age of 17, this was recoded as ‘yes’ or ‘no’ to indicate if they had ever been in care.

#### Group 4: living and working conditions

Maternal education was categorised into six groups ‘degree plus (higher degree and first degree qualifications)’, ‘diploma (in higher education)’, ‘A-levels’, ‘General Certificate of Secondary Education (GCSE) grades A–C’, ‘GCSE grades D–G’ and ‘none of these qualifications’. Parent’s employment status was classified as either ‘both’, ‘one’ or ‘neither’ parents in work (being on leave from work is classed as being in employment). Housing tenure was coded as ‘owner occupied’, ‘private rented’, ‘social housing’ and ‘other’. The response to the question, ‘How common is pollution, grime or other environmental problems?’ was recoded as ‘common’, ‘not common’ and ‘not at all’. Presentation for first antenatal visit was recoded as late if after 12 weeks. Maternal attachment was measured using a 6-item Condon Maternal Attachment Questionnaire^35^ grouped as ‘low (10–21), ‘average’ (22–23) and ‘high (24–27).

#### Group 5: socioeconomic and wider factors

The National Statistics Socio-Economic Classification was used to code job details for main respondents (the majority of which were mothers) as: ‘managerial & professional’, ‘intermediate’, ‘small employers & own account’, ‘lower supervisory & technical’, ‘semi-routine & routine’, ‘never worked & long-term unemployed’. Net household income was reported by identification of the correct band on a show card and grouped into four quartile bands^26^ : ‘£0–£11 000’, ‘£11 000–£22 000’, ‘£22 000–£33 000’ and ‘£33 000+’. Poverty was defined as an equivalised household income 60% below the median before housing costs according to the Organisation for Economic Co-operation and Development Household Equivalence Scale. Families reported receipt of any means-tested benefits, including Jobseekers Allowance, Income Support, Working Families Tax Credit or Disabled Persons Tax Credit. Indices of Multiple Deprivation (IMD) from 2004 which had been retrospectively linked to wave 1 data were used to give small area level deprivation measures.^20^ IMD scores were divided into quintiles, with one the most deprived quintile and five the least deprived.

### Statistical analyses

Analyses were conducted using Stata V.14.2 (StataCorp LP, 2017). Survey weights were applied to take account of clustering, stratification and oversampling in the survey design, and attrition between survey waves, using the svyset command (Pweight=BOVWT2) and svy prefix for regression modelling.^36^ The number of events per variable exceeds 35, the predictors were checked for collinearity, a large number of predictors were used and all were significantly associated with the outcome suggesting a robust logistic regression model with sufficient sample size.^37 38^

Descriptive analysis of each predictor and school readiness was carried out to ascertain the prevalence of each predictor in the sample. Univariable logistic regression analyses calculating ORs and 95% CI were carried out to assess the unadjusted association of each variable with the outcome.

A multivariable logistic regression model including all 29 variables was reduced using automated forward and backwards stepwise selection (using a cut-off p value of 0.1). Dominance analysis (repeated regression analyses on subsets of variables) was used to produce a ranking and weighting for each predictor in model 1.^39^ These rankings were used to specify a more parsimonious model (model 2) containing the top six predictors, selected to maximise parsimony and performance. The IDI using the complete case sample from model 1 was calculated to assess difference in performance between models as the percentage change in individuals being correctly assigned by the model.^40^

The AUROC and its 95% CI was used to measure discriminatory power of the models. Classification, including sensitivity and specificity, was assessed at the maximised probability cut-off point where the sensitivity and specificity curves intersected. Calibration of the model was assessed using the Pearson χ^2^ test.^41^ Bootstrapping was used for internal validation of the final model, without repeating selection of predictors in each bootstrap sample. Model performance was assessed using 1000 bootstrap samples, model optimism was averaged across all iterations to obtain an optimism estimate. An optimism-corrected AUROC, which takes account of overfitting, was calculated by subtracting the optimism estimate from the uncorrected AUROC.^42^

A complete case approach was used for the primary analysis. As a sensitivity analysis, multiple imputation by chained equation was performed to impute missing data using the ‘mi impute chained’ command in Stata. We used predictor variables with relatively little missing data (maternal education, child’s sex, mother’s age at birth of first child) and the outcome as regular variables in the imputation model. As such individuals with missing data for these four items were not included in the final imputed sample (n=11 897). Twenty imputed datasets were generated, and Rubin’s rules were used to calculate results across the imputed datasets.^43^

Robustness tests were carried out in which the final model was tested with an alternative outcome measure for ECD (the British Ability Scales, also tested at age three in the MCS); different coding of outcome and predictor variables (eg, maternal age as a continuous variable); and with the addition of another predictor variable (child care type at age 9 months). See online supplementary file 1 for further details.

10.1136/bmjopen-2018-024851.supp1 Supplementary data

### Ethics and patient and public involvement

Ethical approval for each wave of the MCS was granted by NHS Multicentre Research Ethics Committees.^44^ No further ethical approval was required for this secondary analysis of MCS data. There was no direct patient or public involvement in this analysis. However, the MCS has an ongoing programme of participant and public engagement.

## Results

There were 15 381 singleton children surveyed in MCS2, of which 13 650 had an outcome recorded for school readiness. Of these children 70% (n=9487) had complete data for the outcomes and all the predictor variables. There were no significant differences in the characteristics of the imputed sample and the complete case sample (p value >0.05 for all χ^2^ tests) (table 1); results are reported for complete cases (see online supplementary file 2 for imputed sample results).

10.1136/bmjopen-2018-024851.supp2 Supplementary data

**Table 1:** Description of perinatal, sociodemographic and economic characteristics by school ready of sample and imputed sample

Is child school ready?	Complete cases (n=9487)		Imputed data (n=11 897)	
	Yes (%)	No (%)	Yes (%)	No (%)
All	88.3	11.7	85.5	14.5
**Group 1: demographic and individual factors**				
Gender				
Female	91.6	8.4	89.4	10.6
Male	85.1	14.9	82.6	17.4
Ethnicity				
White	90.4	9.6	88.6	11.4
Mixed	91.1	8.9	84.7	15.3
Indian	79.3	20.7	78.1	21.9
Pakistani and Bangladeshi	55.7	44.3	56.3	43.7
Black or Black British	79.8	20.2	68	32
Other ethnic group	73.6	26.4	74.3	25.7
Mother’s age at birth of first child				
14–19	78	22	76.4	23.6
20–29	87.9	12.1	86.1	13.9
30–39	95	5	94.4	5.6
40+	76.9	23.1	76	24
Birth weight (<2500 g)				
Normal/high	88.8	11.2	86.1	13.9
Low birth weight	80.2	19.8	77.7	22.3
Maternal mental health (diagnosed depression/anxiety)				
No	89	11	86	14
Yes	86	14	84.4	15.6
Child developmental milestones				
Child development score (mean, 95% CI)	19.3 (19.2 to 19.3)	19.9 (19.7 to 20.1)	19.1 (19.0 to 19.1)	19.6 (19.4 to 19.7)
**Group 2: lifestyle factors**				
Duration of breast feeding				
6 months or more	92.5	7.5	90.5	9.5
6 weeks–6 months	89.8	10.2	87.8	12.2
1–6 weeks	88.8	11.2	85.9	14.1
1 week or less	88.8	11.2	86.4	13.6
Never	82.6	17.4	80	20
**Group 3: social and community networks**				
Number of children in family				
One child	92	8	89.1	10.9
Two or three children	87.7	12.3	85	15
Four or more children	71.7	28.3	70.2	29.8
**Group 4: living and working conditions**				
Maternal education				
Degree plus	95.6	4.4	95.1	4.9
Diploma	94.6	5.4	93.9	6.1
A levels	92.7	7.3	92	8
GCSE A-C	88.5	11.5	87.4	12.6
GCSE D-G	81	19	79.1	20.9
None	71.3	28.7	69.2	30.8
Workforce status				
Both parents in work	92.6	7.4	91.6	8.4
One parent in work	85.8	14.2	83.4	16.6
Neither parent in work	68.5	31.5	70.1	29.9
Housing tenure				
Owner occupied	91.9	8.1	90.7	9.3
Private rented	83.8	16.2	80.5	19.5
Social housing	75.8	24.2	74.8	25.2
Other	83.4	16.6	81	19
**Group 5: socioeconomic and wider factors**				
Social class				
Managerial and professional	95.5	4.5	94.6	5.4
Intermediate	93.1	6.9	92.1	7.9
Small employers and own account	91.3	8.7	89.1	10.9
Lower supervisory and technical	87.2	12.8	84	16
Semiroutine and routine	81.9	18.1	80	20
Never worked and long-term unemployed	60.2	39.8	62.1	37.9
Annual income				
£33 000+	95.7	4.3	94.9	5.1
£22 000–£33 000	92.5	7.5	91.7	8.3
£11 000–£22 000	85	15	83.9	16.1
£0–£11 000	73.8	26.2	74.1	25.9

About 11.7% (95% CI 11.0% to 12.3%) of children aged 3 years were classified as not being school ready, but this varied significantly by the parents’ ethnicity, maternal education and social class (table 1). All 29 predictor variables were significantly associated with school readiness in univariable logistic regression analysis (p<0.1), so none were excluded at this stage.

The stepwise method reduced the final multivariable logistic regression model to 13 predictors: child’s sex and ethnicity, mother’s age at birth of first child, birth weight, maternal mental health, child development milestones, duration of breastfeeding, number of children in family, maternal education, parents’ workforce status, housing tenure, social class and annual family income. In the adjusted analysis, Pakistani and Bangladeshi children were four times more likely to not be school ready than white children (OR 4.19, 95% CI 3.14 to 5.58). The full results are shown in table 2. There was no evidence of collinearity.

**Table 2:** Unadjusted and adjusted associations and dominance analysis for the predictor variables in model 1 (13 predictors)

Predictors	Unadjusted OR (95% CI)	Adjusted OR (95% CI)	Weighting (rank)
**Group 1: demographic and individual factors**			
Gender			
Female	1	1	9.5 (5)
Male	1.76 (1.54 to 2.01)	2.03 (1.72 to 2.39)	
Ethnicity			
White	1	1	14.7 (2)
Mixed	1.4 (0.96 to 2.04)	1.42 (0.78 to 2.58)	
Indian	1.85 (1.23 to 2.77)	2.58 (1.65 to 4.03)	
Pakistani and Bangladeshi	5.94 (4.82 to 7.32)	4.27 (3.20 to 5.69)	
Black or Black British	4.06 (2.90 to 5.69)	2.1 (1.13 to 3.88)	
Other ethnic group	2.33 (1.38 to 3.93)	2.92 (1.55 to 5.48)	
Mother’s age at birth of first child			
30–39	1	1	2.9 (11)
40+	2.83 (2.29 to 3.49)	1.05 (0.68 to 1.63)	
20–29	5.57 (4.20 to 7.37)	1.28 (0.98 to 1.66)	
14–19	6.02 (4.84 to 7.48)	1.32 (0.95 to 1.83)	
Birth weight (<2500 g)			
Normal/high	1	1	1.4 (12)
Low birth weight	1.7 (1.34 to 2.16)	1.26 (0.92 to 1.72)	
Maternal mental health (diagnosed depression/anxiety)			
No	1	1	0.4 (13)
Yes	1.33 (1.16 to 1.53)	1.28 (1.07 to 1.53)	
Child developmental milestones			
Developmental score	1.07 (1.05 to 1.10)	1.1 (1.07 to 1.14)	3.9 (11)
**Group 2: lifestyle factors**			
Duration of breast feeding			
6 months or more	1	1	3.9 (10)
6 weeks–6 months	1.25 (1.02 to 1.53)	1.05 (0.81 to 1.36)	
1 week or less	1.67 (1.34 to 2.09)	1.19 (0.89 to 1.59)	
1–6 weeks	1.68 (1.36 to 2.07)	1.25 (0.96 to 1.65)	
Never	2.74 (2.29 to 3.27)	1.49 (1.19 to 1.87)	
**Group 3: social and community networks**			
Number of children in family			
One child	1	1	7.8 (6)
Two or three children	1.44 (1.27 to 1.63)	1.38 (1.15 to 1.66)	
Four or more children	3.71 (3.04 to 4.54)	2.67 (1.94 to 3.68)	
**Group 4: living and working conditions**			
Maternal education			
Degree plus	1	1	13.6 (3)
Diploma	1.3 (0.93 to 1.81)	0.81 (0.53 to 1.24)	
A levels	1.66 (1.22 to 2.25)	1.02 (0.68 to 1.55)	
GCSE A-C	3.02 (2.34 to 3.90)	1.3 (0.89 to 1.88)	
GCSE D-G	5.55 (4.21 to 7.30)	1.54 (1.02 to 2.34)	
None	9.62 (7.61 to 12.16)	1.68 (1.15 to 2.43)	
Workforce status			
Both parents in work	1	1	6.9 (7)
One parent in work	1.79 (1.49 to 2.14)	0.82 (0.67 to 1.00)	
Neither parent in work	5.39 (4.36 to 6.67)	1.21 (0.87 to 1.68)	
Housing tenure			
Owner occupied	1	1	5.7 (8)
Private rented	2.68 (2.16 to 3.33)	1.21 (0.87 to 1.67)	
Social housing	3.89 (3.34 to 4.53)	1.45 (1.16 to 1.81)	
Other	2.65 (2.10 to 3.35)	0.9 (0.62 to 1.30)	
**Group 5: socioeconomic and wider factors**			
Social class			
Managerial and professional	1	1	17.4 (1)
Intermediate	1.5 (1.19 to 1.89)	1.06 (0.77 to 1.45)	
Small employers and own account	2.11 (1.44 to 3.08)	1.41 (0.87 to 2.28)	
Lower supervisory and technical	3.72 (2.76 to 5.00)	1.65 (1.09 to 2.50)	
Semiroutine and routine	4.99 (4.13 to 6.01)	1.97 (1.46 to 2.66)	
Never worked and long-term unemployed	12.07 (9.48 to 15.37)	2.49 (1.69 to 3.66)	
Annual income			
£33 000+	1	1	12.0 (4)
£22 000–£33 000	1.71 (1.31 to 2.25)	1.31 (0.96 to 1.79)	
£11 000–£22 000	3.97 (3.12 to 5.07)	1.64 (1.22 to 2.22)	
£0–£11 000	7.7 (6.10 to 9.72)	2.26 (1.60 to 3.19)	

Dominance analysis showed that social class was the most important predictor (weighting=17.6), followed by ethnic group (weighting=14.7) and maternal education (weighting=13.8) (table 2). Analysis of the predictor weightings suggests that social factors (average weighting 11.3, SD 4.9) are stronger predictors of school readiness than demographic and lifestyle factors (average weighting 5.5, SD 4.9). IDI was used to test the relative performance of models with all (1-13) variables, with variables added in according to their rank from the dominance analysis (online supplementary file 3). These analyses informed the specification of model 2, which comprised the top six predictors: social class, child’s ethnic group, maternal education, income band, sex and number of children (see online supplementary file 4 for model 2 results).

10.1136/bmjopen-2018-024851.supp3 Supplementary data

10.1136/bmjopen-2018-024851.supp4 Supplementary data

**Figure 2:**
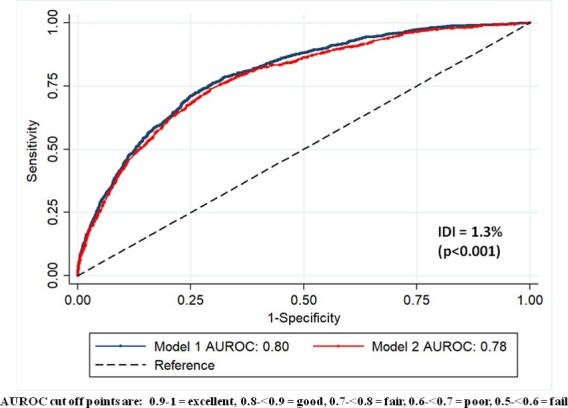
ROC curves for models 1 (13 predictors) and 2 (6 predictors), showing AUROC and IDI. AUROC, area under an ROC curve; IDI, integrated discrimination improvement; ROC, receiver operator characteristic.

The AUROC was 0.80 (95% CI 0.78 to 0.81) for model 1 (n=9487), which indicates a ‘good’ level of discrimination.^19^ The AUROC for model 2 (n=11 146) was 0.78 (95% CI 0.77 to 0.79). Internal validation using bootstrap optimism correction suggests that the model would have good discriminatory power in an independent sample (adjusted AUROC model 1=0.79, model 2=0.76). The Pearson χ^2^ tests were both non-significant indicating adequate calibration (model 1, p=0.07, model 2, p=0.13).^45^ IDI showed there was a small but significant difference in performance, with model 1 resulting in a 1.3% (p≤0.001) improvement in discrimination (figure 2).

**Figure 3:**
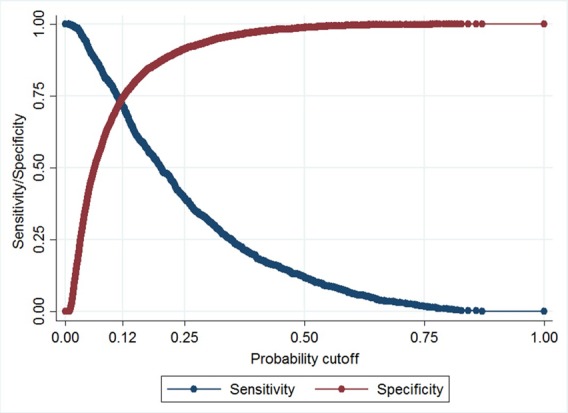
Maximised probability cut-off of sensitivity and specificity of model 1.

Sensitivity and specificity were plotted against probability cut-offs to select the optimal cut-off point to assess the PRM’s classification (model 1, cut-off=0.12; model 2, cut-off=0.14) (figure 3). For model 1, at this cut-off point sensitivity was 72% (95% CI 69.0% to 74.3%) and specificity was 74% (95% CI 73.5% to 75.3%). Sensitivity of model 2 was similar—72% (95% CI 69.9% to 74.5%). Specificity was lower—71% (95% CI 69.6% to 71.4%), so this model would generate more false positive results than the model 1, but performance was still in the acceptable range. At a probability cut-off of 12%, 31% of the screened population tested would be identified as being ‘at risk’ of poor school readiness using model 1.

A sensitivity analysis using an alternative outcome measure (British Ability Scales, BAS), showed that the BSRA measure led to improved discrimination (AUROC=0.79 (95% CI 0.78 to 0.81) for BAS; AUROC=0.80 (95% CI 0.78 to 0.81) for BSRA, p=0.002). See online supplementary file 1 for further details.

## Discussion

### Findings

This study developed a PRM for school readiness at age 3 years using perinatal and early childhood data from the MCS. Model 1 with 13 variables had good discrimination (AUROC=0.80) and classification (sensitivity=72%, specificity=74% at a maximised cut-off). Dominance analysis found the most important variables in predicting school readiness related to socioeconomic conditions (social class, maternal education, family income) and ethnicity. A parsimonious model performed similarly well (AUROC=0.78), suggesting it is possible to predict school readiness at age three fairly well using just six variables from the perinatal period and early infancy.

### Comparison with previous studies

The value added of this study is that it is the first UK study to show that school readiness can be predicted with good discrimination with a small number of variables collected in infancy. The predictors of school readiness identified here corroborate previous findings. Male sex, maternal education, income, family composition, parental employment, housing and breastfeeding have been identified as significant risk factors of delayed ECD in other studies.^4 14 15 17 18 26^ Social factors were the most important predictors, corresponding with current thinking on the social determinants of cognitive development.^6 46^

The model reported here has good predictive strength, and compares favourably to similar PRMs, which with one exception,^17^ achieved only fair or poor discrimination.^14 15 18 47^ Chittleborough *et al* used the ALSPAC UK birth cohort to test the predictive validity of 2 models for ECD.^14^ They used a different outcome measure (School entry assessment aged 4–5) and used six predictors in their model, which appear to be chosen a priori, rather than by a statistical routine. They found that maternal age alone failed to predict ECD (AUROC~0.5), and a model with six predictors achieved only poor discrimination (AUROC=0.67). Camargo-Figuera *et al* used IQ as a measure of ECD and developed a PRM with 12 predictors using the Brazilian Pelotas birth cohort; their model had good discrimination (AUROC=0.8) and calibration, with sensitivity and specificity of 72% and 74%, respectively.^17^ We believe the use of a representative cohort for model development, stepwise regression to select predictor variables and dominance analysis to specify a simplified model contributed to the good performance of this PRM.

### Strengths and limitations

A strength of this study was the use of a representative and contemporary UK cohort study as the data source. This offered a wide range of predictor variables and a large sample size which minimised the likelihood of overfitting. The cohort design also ensured correct temporal ordering and blinding with respect to the predictors. A theoretical model informed the PRM and statistical selection was used to specify variables. Multiple imputation was used to assess the impact of missing data. Bootstrapping showed good internal validity.^48^

There are some limitations of this study to be considered. The main outcome, the BSRA, while validated as a measure of school readiness, was developed in the USA and is not routinely used in the UK.^23^ The BSRA measures a small set of pre-academic skills and as such is a limited measure of child development, which can be defined as including broader behavioural and social skills. However, an analysis of MCS data linked to teacher reports showed that Bracken scores are strongly associated with the broader Early Years Foundation Stage (EYFS) measure of school readiness used in English schools.^4^ The outcome variable was dichotomised to allow ROC curve analysis. We acknowledge the limitations of dichotomising school readiness ethically, conceptually (eg, children develop at different rates) and statistically (ie, loss of information).^49 50^ Longitudinal studies are subject to attrition and non-response which can introduce attrition bias, the use of survey weights partially adjust for this, but it was not possible to use these when calculating the AUROC. Sensitivity analysis using multiple imputation showed the effect of missing data was negligible, similar to other PRMs.^14 15^ Most of the predictor variables were based on maternal self-report which may be subject to recall bias, and external validation was not conducted. The predictor variables identified may not be causally associated with school readiness and there are other predictors which may be associated with the outcome which were not included in this model, for example, the home learning environment (which was not assessed at 9 months in the MCS) and childcare in infancy.^51^

### Policy implications

The existing literature, and these findings, indicate that a PRM could plausibly be used to identify a group of children at high risk of poor ECD who may benefit from early intervention. If implemented as part of a ‘proportionate universalism’ approach,^6^ PRMs could mitigate socioeconomic inequalities by providing early years settings with a mechanism for directing their resources to those children at highest risk of poor cognitive development. With new child and maternity datasets now being collected electronically in England, it may be possible to apply a PRM at population level through the use of linked administrative datasets as has been done in Australia.^15^

Poor cognitive development is associated with a range of negative health and social outcomes and contributes to inequalities in society,^3 5 6^ so this is of public health importance. Chittleborough *et al* showed that even a model with poor discrimination has benefits over just using young maternal age to direct resources.^14^ Similarly, McKean *et al* established that their PRM was better than existing clinical tools used to identify higher-risk children for early intervention.^47^

The practical implications of using such a PRM as a screening tool should be considered. The model reported here would identify 31% of children screened as being ‘at risk’ of delayed school readiness. An exemplar English Local Authority with a total population of 230 000, and 3000 children aged under 1 year would identify 900 ‘at risk’ children per year if the PRM was applied to this cohort. This percentage equates with national data; in 2015/2016, 31% of children in England were not school ready when tested at age 4–5.^11^ However, the overall accuracy of the model is 74%, so over 200 children would be incorrectly classified. PRMs raise ethical issues; labelling very young children as being at risk of poor development could be stigmatising for families, particularly when social factors are the strongest predictors as in this analysis. PRMs would generate false positives (and false negatives), which could cause unnecessary distress and use of resources.

Use of PRMs to identify children at risk of developmental delay should include support and counselling for families, as well as timely access to appropriate interventions. Nelson *et al* 18 comment that Early Intervention services would be overwhelmed by the level of demand generated by such PRMs.^18^ A criterion for screening programmes is that interventions should be available, it is thus important to further consider the implications of using a PRM to assess ECD in the context of available resources. Investment in early intervention would be required, which would have opportunity costs for services locally. Further research is needed to test the external validity of this PRM for example in another cohort or with linked administrative datasets such as the EYFS data from English schools. Alternative modelling approaches which do not require a dichotomous outcome could also be tested. Findings from such models could offer more nuanced predictions on school readiness.

## Conclusion

This study has identified a set of predictive risk factors from the perinatal period and early infancy that can predict school readiness at age 3 with a good level of accuracy. Poor cognitive development is socially patterned, evident from a very young age and leads to persistent disadvantage throughout life. It is possible that PRMs could be used to identify high risk children and target appropriate interventions and resources to improve their developmental trajectories, and to reduce social inequalities early in the life course.

## Supplementary Material

Reviewer comments

Author's manuscript

## References

[1] National Research Council (US) and Institute of Medicine (US) Committee on Integrating the Science of Early Childhood Development. From Neurons to Neighborhoods: The Science of Early Childhood Development. Washington (DC): National Academies Press (US), 2000. 25077268

[2] Black MM, Walker SP, Fernald LCH, et al Early childhood development coming of age: science through the life course. Lancet 2017;389 10.1016/S0140-6736(16)31389-7 PMC5884058 27717614

[3] Heckman JJ Skill formation and the economics of investing in disadvantaged children. Science 2006;312:1900–2. 10.1126/science.1128898 16809525

[4] Hobcraft JN, Kiernan KE Predictive factors from age 3 and infancy for poor child outcomes at age 5 relating to children’s development, behaviour and health: evidence from the Millennium Cohort Study. York: University of York, 2010.

[5] Caspi A, Houts RM, Belsky DW, et al Childhood forecasting of a small segment of the population with large economic burden. Nat Hum Behav 2016;1:0005 10.1038/s41562-016-0005 28706997 PMC5505663

[6] Marmot M, Allen J, Goldblatt P, et al Fair society, healthy lives: strategic review of health inequalities in England post 2010: Marmot Review Team, 2010.

[7] Public Health England. Improving school readiness: creating a better start for London. London, 2015.

[8] Britto PR School Readiness - A conceptual framework. New York, NY: UNICEF, 2012.

[9] Marmot M, Friel S, Bell R, et al Closing the gap in a generation: health equity through action on the social determinants of health. The Lancet 2008;372:1661–9. 10.1016/S0140-6736(08)61690-6 18994664

[10] Abreu L, Roberts N Children’s early years development and school readiness. 2016 https://researchbriefings.parliament.uk/ResearchBriefing/Summary/CDP-2016-0141 (accessed 30 Jan 2019).

[11] Public Health England. Public Health Profiles. 2017 https://fingertips.phe.org.uk/profile-group/child-health (accessed 13 Jun 2017).

[12] GOV.UK. Early years foundation stage profile results: 2017 to 2018. https://www.gov.uk/government/statistics/early-years-foundation-stage-profile-results-2017-to-2018 (accessed 22 Jan 2019).

[13] FPH, FNP. Family Nurse Partnership. Faculty of Public Health 2010, 2015.

[14] Chittleborough CR, Lawlor DA, Lynch JW Young maternal age and poor child development: predictive validity from a birth cohort. Pediatrics 2011;127:e1436–e1444. 10.1542/peds.2010-3222 21536608

[15] Chittleborough CR, Searle AK, Smithers LG, et al How well can poor child development be predicted from early life characteristics?: A whole-of-population data linkage study. Early Child Res Q 2016;35:19–30.

[16] Brownell MD, Ekuma O, Nickel NC, et al A population-based analysis of factors that predict early language and cognitive development. Early Child Res Q 2016;35:6–18. 10.1016/j.ecresq.2015.10.004

[17] Camargo-Figuera FA, Barros AJ, Santos IS, et al Early life determinants of low IQ at age 6 in children from the 2004 Pelotas Birth Cohort: a predictive approach. BMC Pediatr 2014;14:308 10.1186/s12887-014-0308-1 25510879 PMC4272809

[18] Nelson BB, Dudovitz RN, Coker TR, et al Predictors of Poor School Readiness in Children Without Developmental Delay at Age 2. Pediatrics 2016;138:e20154477 10.1542/peds.2015-4477 27432845 PMC4960729

[19] Pepe MS, Janes H, Longton G, et al Limitations of the odds ratio in gauging the performance of a diagnostic, prognostic, or screening marker. Am J Epidemiol 2004;159:882–90. 10.1093/aje/kwh101 15105181

[20] Hansen K Millennium Cohort Study First, Second, Third and Fourth Surveys A Guide to the Datasets. 7th edn London: Centre for Longitudinal Studies, 2012.

[21] Connelly R, Platt L Cohort profile: UK Millennium Cohort Study (MCS). Int J Epidemiol 2014;43:1719–25. 10.1093/ije/dyu001 24550246

[22] Bracken B Bracken Basic Concept Scale–Revised. San Antonio, TX: The Psychological Corporation, 1998.

[23] Panter JE, Bracken BA Validity of the Bracken School Readiness Assessment for predicting first grade readiness. Psychol Sch 2009;46:397–409. 10.1002/pits.20385

[24] Connelly R Millennium Cohort Study Data Note 2013/1: Interpreting Test Scores. London: Centre for Longitudinal Studies, Institute of Education, 2013.

[25] Steyerberg E Clinical prediction models: a practical approach to development, validation, and updating: Springer Science & Business Media, 2008.

[26] Kiernan KE, Mensah FK Maternal indicators in pregnancy and children’s infancy that signal future outcomes for children’s development, behaviour and health: evidence from the Millennium Cohort Study. York: University of York, 2010.

[27] Kiernan KE, Huerta MC Economic deprivation, maternal depression, parenting and children’s cognitive and emotional development in early childhood. Br J Sociol 2008;59:783–806. 10.1111/j.1468-4446.2008.00219.x 19035922

[28] Shenkin SD, Starr JM, Deary IJ Birth weight and cognitive ability in childhood: a systematic review. Psychol Bull 2004;130:989–1013. 10.1037/0033-2909.130.6.989 15535745

[29] Jefferis BJ, Power C, Hertzman C Birth weight, childhood socioeconomic environment, and cognitive development in the 1958 British birth cohort study. BMJ 2002;325:305 10.1136/bmj.325.7359.305 12169505 PMC117769

[30] Kramer MS, Aboud F, Mironova E, et al Breastfeeding and child cognitive development: new evidence from a large randomized trial. Arch Gen Psychiatry 2008;65:578–84. 10.1001/archpsyc.65.5.578 18458209

[31] Walker SP, Wachs TD, Grantham-McGregor S, et al Inequality in early childhood: risk and protective factors for early child development. Lancet 2011;378:1325–38. 10.1016/S0140-6736(11)60555-2 21944375

[32] Murray GK, Jones PB, Kuh D, et al Infant developmental milestones and subsequent cognitive function. Ann Neurol 2007;62:128–36. 10.1002/ana.21120 17487877 PMC3465788

[33] Kelly Y, Sacker A, Gray R, et al Light drinking in pregnancy, a risk for behavioural problems and cognitive deficits at 3 years of age? Int J Epidemiol 2009;38:129–40. 10.1093/ije/dyn230 18974425

[34] Dahlgren G, Whitehead M Policies and strategies to promote social equity in health. Stockh Inst Future Stud 1991.

[35] Condon JT, Corkindale CJ The assessment of parent-to-infant attachment: Development of a self-report questionnaire instrument. J Reprod Infant Psychol 1998;16:57–76. 10.1080/02646839808404558

[36] Ketende SC, Jones EM User Guide to Analysing MCS Data Using STATA: Centre for Longitudinal Studies, Institute of Education, 2011.

[37] Peduzzi P, Concato J, Kemper E, et al A simulation study of the number of events per variable in logistic regression analysis. J Clin Epidemiol 1996;49:1373–9. 10.1016/S0895-4356(96)00236-3 8970487

[38] Courvoisier DS, Combescure C, Agoritsas T, et al Performance of logistic regression modeling: beyond the number of events per variable, the role of data structure. J Clin Epidemiol 2011;64:993–1000. 10.1016/j.jclinepi.2010.11.012 21411281

[39] Azen R, Traxel N Using Dominance Analysis to Determine Predictor Importance in Logistic Regression. Journal of Educational and Behavioral Statistics 2009;34:319–47. 10.3102/1076998609332754

[40] Pencina MJ, D’Agostino RB, Pencina KM, et al Interpreting incremental value of markers added to risk prediction models. Am J Epidemiol 2012;176:473–81. 10.1093/aje/kws207 22875755 PMC3530349

[41] Windmeijer FAG The asymptotic distribution of the sum of weighted squared residuals in binary choice models. Stat Neerl 1990;44:69–78. 10.1111/j.1467-9574.1990.tb01527.x

[42] Austin PC, Steyerberg EW Events per variable (EPV) and the relative performance of different strategies for estimating the out-of-sample validity of logistic regression models. Stat Methods Med Res 2017;26:796–808. 10.1177/0962280214558972 25411322 PMC5394463

[43] White IR, Royston P, Wood AM Multiple imputation using chained equations: Issues and guidance for practice. Stat Med 2011;30:377–99. 10.1002/sim.4067 21225900

[44] Centre for Longitudinal Studies. MCS Ethical Review and Consent. London: Institute of Education, 2014.

[45] Hosmer DW, Hjort NL Goodness-of-fit processes for logistic regression: simulation results. Stat Med 2002;21:2723–38. 10.1002/sim.1200 12228887

[46] Wilkinson RG, Marmot M Social Determinants of Health: The Solid Facts: World Health Organization, 2003.

[47] McKean C, Law J, Mensah F, et al Predicting Meaningful Differences in School-Entry Language Skills from Child and Family Factors Measured at 12 months of Age. Int J Early Child 2016;48:329–51. 10.1007/s13158-016-0174-0

[48] Steyerberg EW, Harrell FE, Borsboom GJJM, et al Internal validation of predictive models. J Clin Epidemiol 2001;54:774–81. 10.1016/S0895-4356(01)00341-9 11470385

[49] Senn S Disappointing dichotomies. Pharm Stat 2003;2:239–40. 10.1002/pst.90

[50] Altman DG, Royston P The cost of dichotomising continuous variables. BMJ 2006;332:1080.1 10.1136/bmj.332.7549.1080 16675816 PMC1458573

[51] Côté SM, Doyle O, Petitclerc A, et al Child care in infancy and cognitive performance until middle childhood in the millennium cohort study. Child Dev 2013;84:1191–208. 10.1111/cdev.12049 23331073

