# Supplementary material for: Development of a predictive risk model for school readiness at age 3 years using the UK Millennium Cohort Study
Source: BMJ Open. 2019 Jun 17;9(6):e024851. doi: 10.1136/bmjopen-2018-024851 (PMC6596936; doi:10.1136/bmjopen-2018-024851)
Supplement: Supplementary data [file bmjopen-2018-024851supp001.pdf]

SUPPLEMENTARY FILE 1

Robustness tests were carried out in which the final model was tested with an alternative outcome measure for early cognitive development (British Ability Scales), different coding of variables and the addition of another predictor variable (child care type at age 9 months).

1. Using BAS as an alternative outcome variable
   An alternative measure of early cognitive development contained in the MSC are the British Ability Scales (BAS), measured at age 3. BAS scores were dichotomised to 1 SD below the mean as cut off for 'fail'. There is a moderate positive correlation between BAS and BSRA scores (r=0.5722, p<0.0001). The table below compares performance of the models; there is a small but statistically significant improvement in discrimination using BSRA as an outcome measure compared to BAS.

Outcome variable	N	AUROC (95% CI)
BSRA	9487	0.80 (0.78,0.81)
BAS	9487	0.79 (0.77,0.80)

Ho: area(xb1) = area(xb6); chi2(1) = 9.20, Prob>chi2 = 0.002

2. Robustness tests of the BSRA outcome measure
   The BSRA cut off used in the main analysis was a mean standardised composite score <85, which is 1 standard deviation below the mean. The standardisation sample was from a US population. As the BSRA has not been validated in the UK, we tested the model using dichotomised percentile ranks instead of MSCS as the outcome variable (cut off point 1 SD below mean).

   There was no significant different in model performance (AUROC=0.80 for both models, p=0.43). There is evidence to suggest that within the Millennium Cohort Study percentile scores can be misleading in indicating the difference between the performance of cohort members because they are on an ordinal, rather than interval, scale. An outcome based on MSCS was therefore retained.

3. Coding of predictor variables
   As a sensitivity analysis the coding of 4 predictor variables was altered: maternal age (from categorical to continuous), developmental scores (from categorical to continuous) and ethnicity (from categorical to binary). The impact of this on final model performance is shown below:

Description	n	AUROC	Comparative AUROC (n=9310)
Final model	9487	0.80	0.79 (0.77,0.81)
Developmental score (continuous)	9487	0.80	0.80 (0.78,0.81)
Maternal age (continuous)	9310	0.79	0.79 (0.78,0.81)
Ethnicity (binary)	9487	0.79	0.79 (0.78,0.80)

Ho: area(xb1) = area(xb2) = area(xb3) = area(xb4); chi2(3) = 9.98; Prob>chi2 = 0.02

In summary, there were small but statistically significant differences between the models. The only change which improved model discrimination was using continuous development

scores, so this was incorporated into the final model. There is a U-shaped relationship between school readiness and maternal age, so there was a clear rationale for including this as a categorical predictor.

4. Testing the impact of an additional predictor
   There are other measures in the MCS which could have been used as predictors in this analysis. We have done a sensitivity analysis adding childcare type at 9 months to the final model. This reduces the overall discrimination of the model (AUROC = 0.77 vs 0.80), however this could be due to missing data as the child care variable is less complete. There is a statistically significant association with school readiness and child care type in the multivariable model, with children in formal child care settings more likely to be school ready than those being looked after by parents (OR = 1.76, p=0.02)

The Stata Do file for all analyses is available at:
https://www.dropbox.com/s/zxsl4cl87imydp0/SchoolreadinessPRM.do?dl=0
