# Supplementary material for: Development of a predictive risk model for school readiness at age 3 years using the UK Millennium Cohort Study
Source: BMJ Open. 2019 Jun 17;9(6):e024851. doi: 10.1136/bmjopen-2018-024851 (PMC6596936; doi:10.1136/bmjopen-2018-024851)
Supplement: Supplementary data [file bmjopen-2018-024851supp002.pdf]

SUPPLEMENTARY FILE 2

Table 1 - Adjusted associations for the predictor variables in model 1 (13 predictors) using multiple imputed data (n=11,879)

Predictor	Adjusted OR (95% CI)	Weighting (rank)
**GROUP 1 - DEMOGRAPHIC & INDIVIDUAL FACTORS**		
**Gender**		8.5 (5)
Female	1	
Male	1.86 (1.62,2.14)	
**Ethnicity**		15.7 (3)
White	1	
Mixed	1.04 (0.62,1.75)	
Indian	2.68 (1.85,3.89)	
Pakistani and Bangladeshi	3.85 (2.94,5.04)	
Black or Black British	2.31 (1.43,3.72)	
Other ethnic group	3.95 (2.30,6.77)	
**Mother's age at birth of first child**		1.5 (12)
30-39	1	
40+	1.05 (0.67,1.64)	
20-29	1.22 (0.99,1.51)	
14-19	1.22 (0.93,1.59)	
**Birth weight (<2500grams)**		1.2 (13)
Normal/high	1	
Low birthweight	1.52 (1.18,1.97)	
**Maternal Mental Health (Diagnosed depression/anxiety)**		1.5 (11)
No	1	
Yes	1.15 (0.98,1.34)	
**Child developmental milestones**		
Developmental score	1.10 (1.07,1.13)	2.8 (10)
**GROUP 2 - LIFESTYLE FACTORS**		
**Duration of breastfeeding**		3.6 (9)
6 months or more	1	
6 weeks - 6 months	1.17 (0.92,1.48)	
One week or less	1.15 (0.90,1.48)	
1 - 6 weeks	1.22 (0.96,1.57)	
Never	1.58 (1.29,1.95)	
**GROUP 3 - SOCIAL & COMMUNITY NETWORKS**		
**Number of children in family**		7.1 (6)
One child	1	
Two or three children	1.40 (1.19,1.63)	
Four or more children	2.48 (1.94,3.16)	
**GROUP 4 - LIVING & WORKING CONDITIONS**		
**Maternal education**		16.7 (2)
Degree plus	1	

Diploma	0.88 (0.61,1.26)	
A levels	1.13 (0.80,1.59)	
GCSE A-C	1.34 (1.01,1.78)	
GCSE D-G	1.72 (1.23,2.39)	
None	1.74 (1.28,2.38)	
**Workforce status**		
Both parents in work	1	
One parent in work	0.94 (0.78,1.12)	6.5 (7)
Neither parent in work	1.21 (0.93,1.57)	
**Housing tenure**		
Owner occupied	1	
Private rented	1.18 (0.90,1.54)	5.5 (8)
Social housing	1.43 (1.18,1.72)	
Other	0.96 (0.69,1.35)	
**GROUP 5 - SOCIOECONOMIC AND WIDER FACTORS**		
**Social class**		
Managerial & professional	1	
Intermediate	0.98 (0.75,1.29)	
Small employers & own account	1.32 (0.87,2.00)	17.6 (1)
Lower supervisory & technical	1.50 (1.06,2.13)	
Semi-routine & routine	1.77 (1.38,2.27)	
Never worked & long-term unemployed	2.19 (1.53,3.15)	
**Annual income**		
£33,000+	1	
£22,000-£33,000	1.33 (1.02,1.72)	11.9 (4)
£11,000-£22,000	1.67 (1.30,2.14)	
£0-£11,000	2.14 (1.60,2.87)	
**ROC Analysis**	**AUROC = 0.79 (95% CI 0.78,0.80)**	
