# Supplementary material for: Development of a predictive risk model for school readiness at age 3 years using the UK Millennium Cohort Study
Source: BMJ Open. 2019 Jun 17;9(6):e024851. doi: 10.1136/bmjopen-2018-024851 (PMC6596936; doi:10.1136/bmjopen-2018-024851)
Supplement: Supplementary data [file bmjopen-2018-024851supp003.pdf]

SUPPLEMENTARY FILE 3

Integrated discrimination improvement (IDI) analysis was run using Stata function 'idi', which compares the discrimination ability between two logistic regression prediction models. In the first stage of this analysis, the IDI of a PRM with just the strongest predictor variable (social class) was compared to a model with all 13 predictors. Adding the additional 12 predictors lead to a 7.3% increase in IDI. In each subsequent analysis, an additional predictor variable was added according to the ranking of variables from the dominance analysis (Table 1).

Predictor	Weighting	Rank
Social Class	17.38	1
Ethnic group	14.66	2
Maternal education	13.55	3
Income band	12	4
Gender	9.54	5
Number of children	7.84	6
Parent's employment	6.9	7
Housing type	5.65	8
Child development	3.9	9
Breastfeeding	3.9	10
Mother's age at birth of first child	2.87	11
Low birth weight	1.42	12
Mental health	0.38	13

Table 1 - Results of the dominance analysis for model 1

The full results of integrated discrimination improvement (IDI) analysis are shown in Table 2.

Variables included	IDI (%)	p	1-IDI
1	7.3%	<0.00001	92.7%
2	5.3%	<0.00001	94.7%
3	3.8%	<0.00001	96.2%
4	3.5%	<0.00001	96.5%
5	2.3%	<0.00001	97.7%
6	1.3%	<0.00001	98.7%
7	1.0%	<0.00001	99.0%
8	0.9%	<0.00001	99.1%
9	0.6%	0.00001	99.4%
10	0.6%	0.00001	99.4%
11	0.2%	0.01402	99.8%
12	0.0%	0.52356	100.0%

Table 2 - Results of integrated discrimination improvement analysis for 12 models

A 6-predictor model was chosen as this offered the optimal balance between parsimony and discrimination.
