# Supplementary material for: Development of a predictive risk model for school readiness at age 3 years using the UK Millennium Cohort Study
Source: BMJ Open. 2019 Jun 17;9(6):e024851. doi: 10.1136/bmjopen-2018-024851 (PMC6596936; doi:10.1136/bmjopen-2018-024851)
Supplement: Supplementary data [file bmjopen-2018-024851supp004.pdf]

SUPPLEMENTARY FILE 4

Table 1 - Adjusted associations for the predictor variables in model 2 (6 predictors) using complete cases (n=11,146) and multiple imputed data (n=11,879). The weightings and rank are from dominance analysis of the complete case sample.

Predictors	Adjusted OR (95% CI) - complete case	Adjusted OR (95% CI) - multiple imputation	Weighting (rank)
**GROUP 1 - DEMOGRAPHIC & INDIVIDUAL FACTORS**			
**Gender**			
Female	1	1	9.9 (5)
Male	1.99 (1.72,2.31)	1.93 (1.68,2.22)	
**Ethnicity**			
White	1	1	13.7 (4)
Mixed	1.2 (0.77,1.88)	1.26 (0.83,1.90)	
Indian	1.64 (1.09,2.47)	1.72 (1.14,2.59)	
Pakistani and Bangladeshi	2.67 (2.10,3.41)	2.71 (2.11,3.47)	
Black or Black British	2.32 (1.52,3.54)	2.69 (1.80,4.02)	
Other ethnic group	1.98 (1.10,3.58)	2.06 (1.27,3.32)	
**GROUP 3 - SOCIAL & COMMUNITY NETWORKS**			
**Number of children in family**			
One child	1	1	9.5 (6)
Two or three children	1.48 (1.27,1.73)	1.45 (1.25,1.69)	
Four or more children	2.89 (2.23,3.75)	2.62 (2.03,3.38)	
**GROUP 4 - LIVING & WORKING CONDITIONS**			
**Maternal education**			
Degree plus	1	1	20.5 (3)
Diploma	0.87 (0.58,1.29)	0.88 (0.60,1.28)	
A levels	1.05 (0.72,1.53)	1.06 (0.74,1.52)	
GCSE A-C	1.43 (1.02,1.99)	1.55 (1.14,2.12)	
GCSE D-G	1.78 (1.23,2.58)	2.14 (1.51,3.03)	
None	2.01 (1.44,2.81)	2.42 (1.77,3.30)	
**GROUP 5 - SOCIOECONOMIC AND WIDER FACTORS**			
**Social class**			
Managerial & professional	1	1	26.0 (1)
Intermediate	1.17 (0.88,1.55)	1.14 (0.86,1.51)	
Small employers & own account	1.44 (0.91,2.28)	1.52 (0.99,2.33)	
Lower supervisory & technical	2.01 (1.42,2.86)	1.92 (1.37,2.68)	
Semi-routine & routine	2.41 (1.86,3.12)	2.16 (1.68,2.78)	
Never worked & long-term unemployed	3.34 (2.41,4.63)	2.95 (2.14,4.07)	
**Annual income**			
£33,000+	1	1	20.6 (2)
£22,000-£33,000	1.33 (0.97,1.81)	2.65 (2.01,3.50)	
£11,000-£22,000	1.88 (1.42,2.50)	1.75 (1.32,2.31)	
£0-£11,000	2.98 (2.26,3.92)	1.29 (0.95,1.75)	

**ROC Analysis**	**AUROC = 0.78** (95% CI 0.77 - 0.79) n=11,146	**AUROC = 0.78** (95% CI 0.77 - 0.79) n=11,879
