# Supplementary material for: Development of a predictive risk model for school readiness at age 3 years using the UK Millennium Cohort Study
Source: BMJ Open. 2019 Jun 17;9(6):e024851. doi: 10.1136/bmjopen-2018-024851 (PMC6596936; doi:10.1136/bmjopen-2018-024851)
Supplement: Author's manuscript [file bmjopen-2018-024851.draft_revisions.pdf]

BMJ Open

BMJ Open is committed to open peer review. As part of this commitment we make the peer review history of every article we publish publicly available.

When an article is published we post the peer reviewers' comments and the authors' responses online. We also post the versions of the paper that were used during peer review. These are the versions that the peer review comments apply to.

The versions of the paper that follow are the versions that were submitted during the peer review process. They are not the versions of record or the final published versions. They should not be cited or distributed as the published version of this manuscript.

BMJ Open is an open access journal and the full, final, typeset and author-corrected version of record of the manuscript is available on our site with no access controls, subscription charges or pay-per-view fees (http://bmjopen.bmj.com).

If you have any questions on BMJ Open's open peer review process please email info.bmjopen@bmj.com

BMJ Open

Predictive Risk Model for School Readiness at age 3 years using the UK Millennium Cohort Study

Journal:	*BMJ Open*
Manuscript ID	bmjopen-2018-024851
Article Type:	Research
Date Submitted by the Author:	17-Jun-2018

Complete List of Authors:	Camacho, Christine; University of Liverpool Department of Public Health and Policy,<br>Straatmann, Viviane; University of Liverpool Department of Public Health and Policy<br>Day, Jennie; University of Liverpool Department of Public Health and Policy<br>Taylor-Robinson, David; University of Liverpool, Department of Public Health and Policy

Keywords:	PUBLIC HEALTH, EPIDEMIOLOGY, SOCIAL MEDICINE

SCHOLARONE™
Manuscripts

For peer review only

Predictive Risk Model for School Readiness at age 3 years using the UK Millennium Cohort Study

Miss CL Camacho*[1], Dr VS Straatmann[1], Dr JC Day[1], Prof DC Taylor-Robinson[1]

Affiliations: [1]Public Health and Policy, University of Liverpool, Liverpool, UK

Address correspondence to: Christine Camacho, Department of Public Health and Policy, University of Liverpool, Liverpool, United Kingdom, L69 3GB, [c.camacho@nhs.net], +44 07973395585

For peer review only

For peer review only - http://bmjopen.bmj.com/site/about/guidelines.xhtml

ABSTRACT

Objectives

The aim of this study was to develop a predictive risk model (PRM) for school readiness measured at age 3 years using perinatal and early infancy data.

Design and Participants

This paper describes the development of a predictive risk model. Predictors were identified from the UK Millennium Cohort Study (MCS) wave 1 data, collected when participants were 9 months old. The outcome was school readiness at age 3 years, measured by the Bracken School Readiness Assessment. Stepwise selection and dominance analysis were used to specify 2 models. The models were compared by the area under the receiver operating characteristic curve (AUROC) and integrated discrimination improvement (IDI).

Results

Data were available for 9,487 complete cases. At age 3, 11.7% (95% CI 11.0-12.3%) of children were not school ready. The variables identified were: parents' Socio-Economic Classification, child's ethnicity, maternal education, income band, sex, household number of children, mother's age, low birth weight, mother's mental health, infant developmental milestones, breastfeeding, parents' employment, housing type. A parsimonious model included the first six listed variables (model 2). The AUROC for model 1 was 0.80 (95% CI 0.78-0.81) and 0.78 (95% CI 0.77-0.79) for model 2. Model 1 resulted in a small improvement in discrimination (IDI=1.3%, p<0.001).

Conclusions

Perinatal and infant risk factors predicted school readiness at age 3 with good discrimination. Social determinants were strong predictors of school readiness. This study demonstrates that school readiness can be predicted by six attributes collected around the time of birth.

For peer review only - http://bmjopen.bmj.com/site/about/guidelines.xhtml

Strengths and limitations of this study

- Use of a large, representative, and contemporary cohort study offered a wide range of predictor variables which minimised the likelihood of overfitting.
- Multiple imputation and bootstrapping were used to evaluate the impact of missing data and internal validity, respectively.
- The main outcome measure, the Bracken School Readiness Assessment, was developed in the US, and is not routinely used in the UK.
- This model was not externally validated, which would have given further indication of generalisability.

INTRODUCTION

Early childhood is critical time for lifelong physical, social, emotional and cognitive development. A wide range of factors are associated with early cognitive development (ECD)[1]. Interventions in the first three years of life improve the trajectory of ECD[2] and deliver the greatest return on investment[3], yet it is unclear how best to identify children at most risk of delayed ECD, to enable appropriate targeting of interventions.

Cognitive development measures in children are good indicators of later educational achievement, predict health and social care needs in adults[4,5], and are associated with long term health outcomes[6]. There has been a growing policy interest in school readiness as a measure of ECD[7], and school readiness is a key public health indicator in children in the UK. Good school readiness lays a platform for future learning, employment and health[8,9]. In 2016, 31% of children in England were deemed not school ready at the end of their reception year (aged 4-5 years)[10].

Predictive risk models (PRMs) are well-established in many clinical disciplines to identify groups or individuals at risk of poor outcomes but there have been few attempts to predict ECD from early childhood characteristics[11–15]. Using PRMs in this context could facilitate targeted early intervention as part of a proportionate universalism approach. The aim of this study was to develop a PRM for school readiness measured at age 3 years using perinatal and early infancy data from the UK Millennium Cohort Study (MCS).

METHODS

Data Source

The PRM was developed and validated using MCS data. The MCS is a nationally representative birth cohort study which recruited 18,550 children born from September 2000 to January 2002, followed up in ongoing data collection waves[16]. The sample was clustered at the level of electoral ward and stratified to allow over representation of children living in deprived areas and areas with high concentrations of ethnic minorities[17]. Survey weightings were used to correct for attrition and non-response[18]. Data were collected from the main responder (usually mothers) by trained interviewers in participants' homes using a

For peer review only - http://bmjopen.bmj.com/site/about/guidelines.xhtml

For peer review only

combination of interviews and self-completed questions. All singleton children in the first (aged 9 months) and second (aged 3 years) waves of the MCS with completed data for the outcome and predictors were eligible for inclusion (n=9,487).

Outcome

School readiness was measured at age 3 using the Bracken School Readiness Assessment (BSRA) which consists of 6 subtests relating to colours, letters, numbers/counting, sizes, comparisons and shapes[19]. The BSRA and its predecessors have demonstrated good reliability[20] and validity against other measures and teacher assessments[21].

The BSRA raw scores were summed and adjusted for age to provide a standardised composite score[19]. Scores were grouped into 5 categories based on the mean standardised score: very advanced (131-149), advanced (116-130), average (85-115), delayed (70-84) and very delayed (56-69)[22]. BSRA scores were recoded to a binary variable of either school ready ≥85 very advanced/advanced/average) or not school ready (<85; delayed/very delayed)[23].

Predictors

29 variables which were identified from previous research to predict cognitive development and were included in the MCS[1,2,4,6,24–31]. The selected predictor variables were grouped according to the Dahlgren and Whitehead theoretical model[32] of social determinants of health as depicted in Figure 1.

<<Figure 1 here>>

Group 1 – Demographic and Individual factors

Demographic characteristics included child sex, maternal ethnicity, child weight, pre-term birth, mother's age, home language, maternal mental health and child development categorised as shown in Box 1.

Box 1 – Coding of Group 1 demographic and individual factors

Categorisation of Demographic and Individual factors
Child sex – 'female' and 'male'
Maternal ethnicity – 'white', 'mixed', 'Indian', 'Pakistani and Bangladeshi', 'Black' and 'other'
Child weight at birth – low (<2.5kg) or normal/high (≥2.5kg)
Preterm birth – gestation period less than 37 weeks
Mother's age in years at birth of first child – grouped into 4 categories (14-19, 20-29, 30-39, 40+ years)
Home language – 'English only', 'English and another language', 'another language only'
Mental health (1) – Sad or low for >2 weeks since baby, coded as 'yes' or 'no'
Mental health (2) – Diagnosis of depression or serious anxiety, coded as 'yes' or 'no'
Mental health (3) – 9-item modified version of the Rutter Malaise Inventory[39], coded as 'low' or (0-3) 'high' (4-9) scores[27].
Child development – 8 items from Denver Developmental Screening Test and 5 items from MacArthur Communicative Development Inventory, coded as 'above average' (13-17), 'average' (18-19) and 'below average' (20-36).

Group 2 – Lifestyle Factors

For peer review only - http://bmjopen.bmj.com/site/about/guidelines.xhtml

For peer review only

Self-reported maternal smoking was coded as 'never smoked', 'smoked before pregnancy' and 'smoked during pregnancy'. Maternal alcohol consumption during pregnancy were categorised as 'never or very infrequent', 'occasional', 'regularly' and 'most or everyday'. Breastfeeding duration was grouped as 'never', 'one week or less', '1 – 6 weeks', '6 weeks – 6 months' and 'over 6 months'.

Group 3 – Social and Community Factors
The number of children in household was coded as '1', '2-3' or '4+', and being the eldest or only child was recorded as 'yes' or 'no'. The number of parents or carers was either '1' or '2'. Mothers were asked how much time they had spent time in care before the age of 17, this was recoded as 'yes' or 'no' to indicate if they had ever been in care.

Group 4 – Living and Working Conditions
Maternal education was categorised into six groups 'degree plus (higher degree and first degree qualifications)', 'diploma (in higher education)', 'A-levels', 'GCSE grades A–C', 'GCSE grades D–G' and 'none of these qualifications'. Parent's employment status was classified as either 'both', 'one' or 'neither' parents in work. Housing tenure was coded as 'owner occupied', 'private rented', 'social housing' and 'other'. The response to the question, "How common is pollution, grime or other environmental problems?" was recorded as 'common', 'not common' and 'not at all'. Presentation for first antenatal visit was recorded as late if after 12 weeks. Maternal attachment was measured using a 6-item Condon Maternal Attachment Questionnaire[33] grouped as 'low (10-21), 'average' (22-23) and 'high (24-27) .

Group 5 – Socioeconomic and Wider Factors
The National Statistics Socio-Economic Classification (NS-SEC) was used to categorise mothers as: 'managerial & professional', 'intermediate', 'small employers & own account', 'lower supervisory & technical', 'semi-routine & routine', 'never worked & long-term unemployed'. Net household income was reported by identification of the correct band on a show card and grouped into 4 quartile bands[24]: '£0-£11,000', '£11,000-£22,000', '£22,000-£33,000' and '£33,000+'. Poverty was defined as an equivalised household income 60% below the median before housing costs according to the Organisation for Economic Co-operation and Development Household Equivalence Scale. Families reported receipt of any means-tested benefits, including Jobseekers Allowance, Income Support, Working Families Tax Credit or Disabled Persons Tax Credit. Indices of Multiple Deprivation (IMD) from 2004 were used as an indicator of area level deprivation. IMD scores were divided into quintiles, with 1 the most deprived quintile, and 5 the least deprived.

Statistical analyses

Analyses were conducted using Stata v14.2 (StataCorp LP, 2017). Survey weights were applied to take account of clustering, stratification and oversampling in the survey design, and attrition between survey waves[34]. A calculation based on the number of events per variable (EPV) was used to determine sample size for the PRM. The EPV for this study is 68, which exceeds the EPV of 10 suggested to minimise overfitting[35], so the sample is sufficiently large to test 29 predictor variables.

For peer review only - http://bmjopen.bmj.com/site/about/guidelines.xhtml

Descriptive analysis of each predictor and school readiness was carried out to ascertain the prevalence of each predictor in the sample. Univariable logistic regression analyses calculating odds ratios (ORs) and 95% confidence intervals (95% CI) were carried out to assess the unadjusted association of each variable with the outcome.

A multivariable logistic regression model including all 29 variables was reduced using automated forward and backwards stepwise selection (using a cut off p-value of 0.1). The predictors included in the resulting model (model 1) were checked for collinearity. Dominance analysis (repeated regression analyses on subsets of variables) was used to produce a ranking and weighting for each predictor in model 1[36]. These rankings were used to specify a more parsimonious model (model 2) containing the top 6 predictors, selected to maximise parsimony and performance. The integrated discrimination improvement (IDI) was also calculated to assess difference in performance between models as the percentage change in individuals being correctly assigned by the model[37].

The area under the ROC curve (AUROC) and its 95% CI was used to measure discriminatory power of the models. Classification, including sensitivity and specificity, was assessed at the maximised probability cut off point where the sensitivity and specificity curves intersected. Calibration of the model was assessed using the Pearson Chi-squared test[38]. Bootstrapping was used for internal validation of the model using 1000 iterations. An optimised AUROC, which takes account of overfitting, was calculated as the difference between baseline model performance and performance across the bootstrap samples[39].

A complete case approach was used for the primary analysis. As a sensitivity analysis, multiple imputation by chained equation was performed to impute missing data (imputed sample, n=13,650). Variables from the first sweep and the outcome variable were used to shape the imputation of the missing data (maternal education, child's sex, mother's age at birth of first child and school readiness at age 3). Twenty imputed datasets were generated, and Rubin's rules were used to calculate results across the imputed datasets[40]. Ethical approval for each wave of the MCS was granted by NHS Multicentre Research Ethics Committees[41]. No further ethical approval was required for this secondary analysis of MCS data.

Patient and public involvement

There was no direct patient or public involvement in this analysis. However the MCS has an ongoing programme of participant and public engagement.

RESULTS

There were 15,381 singleton children surveyed in MCS2, of which 13,650 had an outcome recorded for school readiness. Of these children 70% (n=9,487) had complete data for the outcomes and all the predictor variables. The characteristics of the imputed sample were similar to the complete case sample (Table 1); results are reported for complete cases (see Supplementary file 1 for imputed sample results).

For peer review only - http://bmjopen.bmj.com/site/about/guidelines.xhtml

Table 1 - Description of perinatal, sociodemographic and economic characteristics by school ready of sample and imputed sample

	Complete Cases (n=9,487)		Imputed Data (n=13,650)	
**Is Child School Ready?**	**Yes (%)**	**No (%)**	**Yes (%)**	**No (%)**
All	88.3	11.7	85.5	14.5
**GROUP 1 - DEMOGRAPHIC & INDIVIDUAL FACTORS**				
**Gender**				
Female	91.6	8.4	89.4	10.6
Male	85.1	14.9	82.6	17.4
**Ethnicity**				
White	90.4	9.6	88.6	11.4
Mixed	91.1	8.9	84.7	15.3
Indian	79.3	20.7	78.1	21.9
Pakistani and Bangladeshi	55.7	44.3	56.3	43.7
Black or Black British	79.8	20.2	68.0	32.0
Other ethnic group	73.6	26.4	74.3	25.7
**Mother's age at birth of first child**				
14-19	78.0	22.0	76.4	23.6
20-29	87.9	12.1	86.1	13.9
30-39	95.0	5.0	94.4	5.6
40+	76.9	23.1	76.0	24.0
**Birth weight (<2500grams)**				
normal/high	88.8	11.2	86.1	13.9
low birthweight	80.2	19.8	77.7	22.3
**Maternal Mental Health (Diagnosed depression/anxiety)**				
No	89.0	11.0	86.0	14.0
Yes	86.0	14.0	84.4	15.6
**Child developmental milestones**				
Above average	90.0	10.0	87.5	12.5
Average	89.2	10.8	86.8	13.2
Below average	86.6	13.4	83.4	16.6
**GROUP 2 - LIFESTYLE FACTORS**				
**Duration of breastfeeding**				
6 months or more	92.5	7.5	90.5	9.5
6 weeks - 6 months	89.8	10.2	87.8	12.2
1 - 6 weeks	88.8	11.2	85.9	14.1
one week or less	88.8	11.2	86.4	13.6
Never	82.6	17.4	80.0	20.0
**GROUP 3 - SOCIAL & COMMUNITY NETWORKS**				
**Number of children in family**				
One child	92.0	8.0	89.1	10.9
Two or three children	87.7	12.3	85.0	15.0
Four or more children	71.7	28.3	70.2	29.8
**Maternal education**				
Degree plus	95.6	4.4	95.1	4.9

For peer review only - http://bmjopen.bmj.com/site/about/guidelines.xhtml

Diploma	94.6	5.4	93.9	6.1
A levels	92.7	7.3	92.0	8.0
GCSE A-C	88.5	11.5	87.4	12.6
GCSE D-G	81.0	19.0	79.1	20.9
None	71.3	28.7	69.2	30.8
**GROUP 4 - LIVING & WORKING CONDITIONS**				
**Workforce status**				
Both parents in work	92.6	7.4	91.6	8.4
One parent in work	85.8	14.2	83.4	16.6
Neither parent in work	68.5	31.5	70.1	29.9
**GROUP 5 - SOCIOECONOMIC AND WIDER FACTORS**				
**Housing tenure**				
Owner occupied	91.9	8.1	90.7	9.3
Private rented	83.8	16.2	80.5	19.5
Social housing	75.8	24.2	74.8	25.2
Other	83.4	16.6	81.0	19.0
**Social class**				
managerial & professional	95.5	4.5	94.6	5.4
intermediate	93.1	6.9	92.1	7.9
small employers & own account	91.3	8.7	89.1	10.9
lower supervisory & technical	87.2	12.8	84.0	16.0
semi-routine & routine	81.9	18.1	80.0	20.0
never worked & long-term unemployed	60.2	39.8	62.1	37.9
**Annual income**				
£33,000+	95.7	4.3	94.9	5.1
£22,000-£33,000	92.5	7.5	91.7	8.3
£11,000-£22,000	85.0	15.0	83.9	16.1
£0-£11,000	73.8	26.2	74.1	25.9

11.7% (95%CI 11.0-12.3%) of children aged 3 years were classified as not being school ready, but this varied significantly by the parents' ethnicity, maternal education and social class (Table 1). All 29 predictor variables were significantly associated with school readiness in univariable logistic regression analysis ($p<0.1$), so none were excluded at this stage.

The stepwise method reduced the final multivariable logistic regression model to 13 predictors: child's sex and ethnicity, mother's age at birth of first child, birthweight, maternal mental health, child development milestones, duration of breastfeeding, number of children in family, maternal education, parents' workforce status, housing tenure, social class and annual family income. In the adjusted analysis, Pakistani and Bangladeshi children were 4 times more likely to not be school ready than white children (OR 4.19 95% CI 3.14-5.58). The full results are shown in Table 2. There was no evidence of collinearity.

Table 2 - Unadjusted and adjusted associations and dominance analysis for the predictor variables in model 1 (13 predictors)

For peer review only - http://bmjopen.bmj.com/site/about/guidelines.xhtml

Predictors	Unadjusted OR (95% CI)	Adjusted OR (95% CI)	Weighting (rank)
**GROUP 1 - DEMOGRAPHIC & INDIVIDUAL FACTORS**			
**Gender**			
Female	1	1	9.8 (5)
Male	1.76 (1.54,2.01)	2.05 (1.73,2.41)	
**Ethnicity**			
White	1	1	14.7 (2)
Mixed	1.4 (0.96,2.04)	1.39 (0.77,2.53)	
Indian	1.85 (1.23,2.77)	2.54 (1.64,3.96)	
Pakistani and Bangladeshi	5.94 (4.82,7.32)	4.19 (3.14,5.58)	
Black or Black British	4.06 (2.90,5.69)	1.99 (1.09,3.63)	
Other ethnic group	2.33 (1.38,3.93)	2.92 (1.57,5.43)	
**Mother's age at birth of first child**			
30-39	1	1	2.9 (10)
40+	2.83 (2.29,3.49)	1.04 (0.67,1.61)	
20-29	5.57 (4.20,7.37)	1.26 (0.96,1.64)	
14-19	6.02 (4.84,7.48)	1.29 (0.93,1.79)	
**Birth weight (<2500grams)**			
Normal/high	1	1	1.6 (12)
Low birthweight	1.7 (1.34,2.16)	1.39 (1.02,1.90)	
**Maternal Mental Health (Diagnosed depression/anxiety)**			
No	1	1	0.4 (13)
Yes	1.33 (1.16,1.53)	1.29 (1.08,1.54)	
**Child developmental milestones**			
Above average	1	1	2.4 (11)
Average	1.04 (0.89,1.22)	1.38 (1.11,1.72)	
Below average	1.4 (1.20,1.64)	1.81 (1.46,2.23)	
**GROUP 2 - LIFESTYLE FACTORS**			
**Duration of breastfeeding**			
6 months or more	1	1	4.0 (9)
6 weeks - 6 months	1.25 (1.02,1.53)	1.04 (0.80,1.35)	
One week or less	1.67 (1.34,2.09)	1.19 (0.89,1.60)	
1 - 6 weeks	1.68 (1.36,2.07)	1.25 (0.95,1.63)	
Never	2.74 (2.29,3.27)	1.49 (1.19,1.87)	
**GROUP 3 - SOCIAL & COMMUNITY NETWORKS**			
**Number of children in family**			
One child	1	1	8.1 (6)
Two or three children	1.44 (1.27,1.63)	1.4 (1.17,1.69)	
Four or more children	3.71 (3.04,4.54)	2.74 (1.99,3.76)	
**GROUP 4 - LIVING & WORKING CONDITIONS**			
**Maternal education**			
Degree plus	1	1	13.8 (3)
Diploma	1.3 (0.93,1.81)	0.8 (0.52,1.22)	
A levels	1.66 (1.22,2.25)	1.02 (0.67,1.53)	

For peer review only - http://bmjopen.bmj.com/site/about/guidelines.xhtml

GCSE A-C	3.02 (2.34,3.90)	1.29 (0.88,1.87)	
GCSE D-G	5.55 (4.21,7.30)	1.54 (1.02,2.34)	
None	9.62 (7.61,12.16)	1.68 (1.15,2.45)	
**Workforce status**			
Both parents in work	1	1	
One parent in work	1.79 (1.49,2.14)	0.81 (0.66,1.00)	7.0 (7)
Neither parent in work	5.39 (4.36,6.67)	1.2 (0.86,1.67)	
**Housing tenure**			
Owner occupied	1	1	
Private rented	2.68 (2.16,3.33)	1.2 (0.87,1.66)	5.7 (8)
Social housing	3.89 (3.34,4.53)	1.46 (1.17,1.82)	
Other	2.65 (2.10,3.35)	0.89 (0.62,1.29)	
**GROUP 5 - SOCIOECONOMIC AND WIDER FACTORS**			
**Social class**			
Managerial & professional	1	1	
Intermediate	1.5 (1.19,1.89)	1.05 (0.77,1.45)	
Small employers & own account	2.11 (1.44,3.08)	1.42 (0.87,2.32)	17.6 (1)
Lower supervisory & technical	3.72 (2.76,5.00)	1.67 (1.11,2.52)	
Semi-routine & routine	4.99 (4.13,6.01)	1.97 (1.46,2.67)	
Never worked & long-term unemployed	12.07 (9.48,15.37)	2.47 (1.68,3.63)	
**Annual income**			
£33,000+	1	1	
£22,000-£33,000	1.71 (1.31,2.25)	1.3 (0.94,1.78)	12.2 (4)
£11,000-£22,000	3.97 (3.12,5.07)	1.65 (1.22,2.24)	
£0-£11,000	7.7 (6.10,9.72)	2.26 (1.59,3.20)	

Dominance analysis showed that social class was the most important predictor (weighting=17.6), followed by ethnic group (weighting=14.7) and maternal education (weighting=13.8) (Table 2). Analysis of the predictor weightings suggests that social factors (average weighting 11.3, SD 4.9) are stronger predictors of school readiness than demographic and lifestyle factors (average weighting 5.5, SD 4.9).

The AUROC was 0.80 (95% CI 0.78-0.81) for model 1 (n=9,487), which indicates a "good" level of discrimination[42]. The AUROC for model 2 (n=11,146) was 0.78 (95% CI 0.77-0.79). Internal validation using bootstrap optimism suggests that the model would have good discriminatory power in an independent sample (adjusted AUROC 0.79). The Pearson Chi-squared tests were both non-significant indicating adequate calibration (model 1, p=0.07, model 2, p=0.13)[43]. IDI showed there was a small but significant difference in performance, with model 1 resulting in a 1.3% (p=<0.001) improvement in discrimination (Figure 2). IDI was also used to test the relative performance of models with all (1-13) variables, with variables added in according to their rank from the dominance analysis. These analyses informed the choice of a top 6-predictor model (social class, child's ethnic group, maternal education, income band, sex and number of children) (Supplementary material 2).

<<Figure 2 here>>

For peer review only - http://bmjopen.bmj.com/site/about/guidelines.xhtml

Sensitivity and specificity were plotted against probability cut-offs to select the optimal cut off point to assess the PRM's classification (model 1, cut-off=0.12; model 2, cut-off=0.14) (Figure 3**Error! Reference source not found.**). For model 1, at this cut-off point sensitivity was 72% (95% CI 69.0%-74.3%) and specificity was 74% (95% CI 73.5%-75.3%). Sensitivity of model 2 was similar - 72% (95% CI 69.9%-74.5%). Specificity was lower - 71% (95% CI 69.6%-71.4%), so this model would generate more false positive results than the model 1, but performance was still in the acceptable range. At a probability cut-off of 12%, 31% of the screened population tested would be identified as being at high risk of poor school readiness using model 1.

<<Figure 3 here>>

For peer review only

DISCUSSION

Findings

This study developed a PRM for school readiness at age 3 years using perinatal and early childhood data from the MCS. Model 1 with 13 variables had good discrimination (AUROC=0.80) and classification (sensitivity=72%, specificity= 74% at a maximised cut off). Dominance analysis found the most important variables in predicting school readiness related to socioeconomic conditions (social class, maternal education, family income) and ethnicity. A parsimonious model performed similarly well (AUROC=0.78), suggesting it is possible to predict school readiness at age 3 using just six variables from the perinatal period and early infancy.

Comparison with previous studies

The predictors of school readiness identified here corroborate previous findings. Male sex, maternal education, income, family composition, parental employment, housing and breastfeeding have been identified as significant risk factors of ECD in other studies[4,11,12,14,15,24]. Social factors were the most important predictors, corresponding with current thinking on the social determinants of cognitive development[6,44].

A few recent studies have used PRM and ROC curves to analyse the association between perinatal and early childhood predictors with cognitive development, but this is the first UK study to develop a PRM with a good level of predictive discrimination for early cognitive development (ECD)[11,12,14,15,45]. The model reported here has good predictive strength, and compares favourably to similar PRMs, which with one exception[14], achieved only fair or poor discrimination[11,12,15,45]. Chittleborough et al used the ALSPAC UK birth cohort to test the predictive validity of 2 models for ECD[11]. They found that maternal age alone failed to predict ECD (AUROC~0.5), and a model with 6 predictors achieved only poor discrimination (AUROC=0.67). Camargo-Figuera et al used IQ as a measure of ECD and developed a PRM with 12 predictors using the Brazilian Pelotas birth cohort; their model had good discrimination (AUROC=0.8) and calibration, with sensitivity and specificity of 72% and 74% respectively[14].

Strengths and Limitations

For peer review only - http://bmjopen.bmj.com/site/about/guidelines.xhtml

A strength of this study was the use of representative and contemporary UK cohort study as the data source, this offered a wide range of predictor variables and a large sample size which minimised the likelihood of overfitting. The cohort design also ensured correct temporal ordering and blinding with respect to the predictors. A theoretical model informed the PRM and statistical selection was used to specify variables. Multiple imputation was used to assess the impact of missing data. Bootstrapping showed good internal validity suggesting the model would be generalisable to another population[39].

There are some limitations of this study to be considered. The main outcome, the BSRA, whilst validated as a measure of school readiness, was developed in the US and is not routinely used in the UK[21]. Many variables were dichotomised or grouped, which may be less sensitive than continuous measures. Longitudinal studies are subject to attrition and non-response which can introduce attrition bias, the use of survey weights partially adjust for this, but it was not possible to use these when calculating the AUROC. Sensitivity analysis using multiple imputation showed the effect of missing data was negligible, similar to other PRMs[11,12]. Most of the predictor variables were based on maternal self-report which may be subject to recall bias, and external validation was not conducted.

Policy Implications

The existing literature, and these findings, indicate that a PRM could plausibly be used to identify a group of children at high risk of poor ECD who may benefit from early intervention. If implemented as part of a "proportionate universalism" approach[6], PRMs could mitigate socioeconomic inequalities by providing early years settings with a mechanism for directing their resources to those children at highest risk of poor cognitive development. With new child and maternity datasets now being collected electronically in England, it may be possible to apply a PRM at population level through the use of linked administrative datasets as has been done in Australia[12].

Poor cognitive development is associated with a range of negative health and social outcomes and contributes to inequalities in society[3,5,6], so this is of public health importance. Chittleborough et al showed that even a model with poor discrimination has benefits over just using young maternal age to direct resources[11]. Similarly, McKean et al established that their PRM was better than existing clinical tools used to identify higher-risk children for early intervention[45].

The practical implications of using such a PRM as a screening tool should be considered. The model reported here would identify 31% of children at high risk of not being school ready. An average English Local Authority with a population of 230,000 would therefore have 900 'at risk' children per year. This percentage equates with national data; in 2015/16, 31% of children in England were not school ready when tested at age 4-5[10]. However, Nelson et al (2016) comment that Early Intervention services would be overwhelmed by the level of demand generated by such PRMs[15]. A criterion for screening programmes is that interventions should be available, it is thus important to further consider the implications of using a PRM to assess ECD in the context of available resources.

For peer review only - http://bmjopen.bmj.com/site/about/guidelines.xhtml

Further research is needed to test the external validity of predictive risk models for ECD for example in another cohort or with linked administrative datasets. PRMs raise ethical issues, labelling very young children as being high risk of poor development could be stigmatising for families. PRMs would generate false positives (and false negatives), which could cause unnecessary distress. Use of PRMs to identify children at risk of developmental delay should include support and counselling for families, as well as timely access to appropriate interventions. Investment in early intervention would be required, which would have opportunity costs for services locally.

CONCLUSION

This study has identified a set of predictive risk factors from the perinatal period and early infancy that can predict school readiness at age 3 with a good level of accuracy. Poor cognitive development is socially patterned, evident from a very young age and leads to persistent disadvantage throughout life. It is possible that PRMs could be used to identify high risk children and target appropriate interventions and resources to improve their developmental trajectories, and to reduce social inequalities early in the life course.

STATEMENTS

Funding

This research received no specific grant from any funding agency in the public, commercial or not for profit sectors. DTR is funded by the MRC on a Clinician Scientist Fellowship (MR/P008577/1).

Competing Interests

We confirm that authors have no conflicts of interest to disclose.

Contributors

CLC, JCD and DTR planned the study. CLC and VSS conducted the analysis under the supervision of DTR. CLC led the drafting of the manuscript. All authors contributed to data interpretation, manuscript drafting and revisions and agreed the submitted version of the manuscript.

Data Sharing

The Millennium Cohort Study dataset is available from the UK Data Service.

ACKNOWLEDGEMENTS

We would like to acknowledge all the families and researchers who are part of the UK Millennium Cohort Study, without whom this research would not have been possible.

For peer review only - http://bmjopen.bmj.com/site/about/guidelines.xhtml

REFERENCES

1 National Research Council (US) and Institute of Medicine (US) Committee on Integrating the Science of Early Childhood Development. *From Neurons to Neighborhoods: The Science of Early Childhood Development*. Washington (DC): : National Academies Press (US) 2000. http://www.ncbi.nlm.nih.gov/books/NBK225557/

2 Black MM, Walker SP, Fernald LC, *et al.* Early childhood development coming of age: science through the life course. *The Lancet* 2016.

3 Heckman JJ. Skill formation and the economics of investing in disadvantaged children. *Science* 2006;**312**:1900–2. doi:312/5782/1900 [pii]

4 Hobcraft JN, Kiernan KE. Predictive factors from age 3 and infancy for poor child outcomes at age 5 relating to children's development, behaviour and health: evidence from the Millennium Cohort Study. York: : University of York 2010. http://www.york.ac.uk/media/spsw/documents/research-and-publications/HobcraftKiernan2010PredictiveFactorsChildrensDevelopmentMillenniumCohort.pdf (accessed 28 May 2017).

5 Caspi A, Houts RM, Belsky DW, *et al.* Childhood forecasting of a small segment of the population with large economic burden. *Nat Hum Behav* 2016;**1**:0005.

6 Marmot M, Allen J, Goldblatt P, *et al.* Fair society, healthy lives: strategic review of health inequalities in England post 2010. Marmot Review Team 2010.

7 Public Health England. Improving school readiness: creating a better start for London. London: 2015.

8 Pia Rebello Britto. School Readiness - A conceptual framework. New York, NY: : UNICEF 2012.

9 Marmot M, Friel S, Bell R, *et al.* Closing the gap in a generation: health equity through action on the social determinants of health. *The Lancet* 2008;**372**:1661–9. doi:10.1016/S0140-6736(08)61690-6

10 Public Health England. Public Health Profiles. 2017.https://fingertips.phe.org.uk/profile-group/child-health (accessed 13 Jun 2017).

11 Chittleborough CR, Lawlor DA, Lynch JW. Young Maternal Age and Poor Child Development: Predictive Validity From a Birth Cohort. *Pediatrics* 2011;**127**:e1436–44. doi:10.1542/peds.2010-3222

12 Chittleborough CR, Searle AK, Smithers LG, *et al.* How well can poor child development be predicted from early life characteristics?: A whole-of-population data linkage study. *Early Child Res Q* 2016;**35**:19–30.

13 Brownell MD, Ekuma O, Nickel NC, *et al.* A population-based analysis of factors that predict early language and cognitive development. *Early Child Res Q* 2016;**35**:6–18.

14 Camargo-Figuera FA, Barros AJ, Santos IS, *et al.* Early life determinants of low IQ at age 6 in children from the 2004 Pelotas Birth Cohort: a predictive approach. *BMC Pediatr* 2014;**14**:308. doi:10.1186/s12887-014-0308-1

For peer review only - http://bmjopen.bmj.com/site/about/guidelines.xhtml

15 Nelson BB, Dudovitz RN, Coker TR, *et al.* Predictors of Poor School Readiness in Children Without Developmental Delay at Age 2. *Pediatrics* 2016;**138**:e20154477. doi:10.1542/peds.2015-4477

16 Connelly R, Platt L. Cohort Profile: UK Millennium Cohort Study (MCS). *Int J Epidemiol* 2014;**43**:1719–25. doi:10.1093/ije/dyu001

17 Kirstine Hansen. Millennium Cohort Study First, Second, Third and Fourth Surveys. A Guide to the Datasets (Seventh Edition). London: : Centre for Longitudinal Studies 2012.

18 Ian Plewis. The Millennium Cohort Study: Technical Report on Sampling. London: : Institute of Education 2007.

19 Kirstine Henson. Millennium Cohort Study. First, Second, Third and Fourth Surveys. A Guide to the Datasets. Centre for Longitudinal Studies 2012. http://www.cls.ioe.ac.uk/shared/get-file.ashx?id=598&itemtype=document

20 Bracken B. Bracken Basic Concept Scale–Revised. San Antonio, TX: : The Psychological Corporation 1998.

21 Panter JE, Bracken BA. Validity of the Bracken School Readiness Assessment for predicting first grade readiness. *Psychol Sch* 2009;**46**:397–409.

22 Connelly R. Millennium Cohort Study Data Note 2013/1: Interpreting Test Scores. London: : Centre for Longitudinal Studies, Institute of Education 2013. http://www.cls.ioe.ac.uk/ (accessed 4 Mar 2018).

23 Steyerberg E. *Clinical prediction models: a practical approach to development, validation, and updating*. Springer Science & Business Media 2008. https://books.google.co.uk/books?hl=en&lr=&id=kHGK58cLsMIC&oi=fnd&pg=PR2&dq=Clinical+Prediction+Models+A+Practical+Approach+to+Development,+Validation,+and+Updating&ots=TNSaD1dEhk&sig=YhVMJY6PfLiylv_T1qMJYLMvLiI

24 Kiernan KE, Mensah FK. Maternal indicators in pregnancy and children's infancy that signal future outcomes for children's development, behaviour and health: evidence from the Millennium Cohort Study. York: : University of York 2010.

25 Kiernan KE, Huerta MC. Economic deprivation, maternal depression, parenting and children's cognitive and emotional development in early childhood. *Br J Sociol* 2008;**59**:783–806.

26 Shenkin SD, Starr JM, Deary IJ. Birth weight and cognitive ability in childhood: a systematic review. *Psychol Bull* 2004;**130**:989.

27 Jefferis BJMH, Power C, Hertzman C. Birth weight, childhood socioeconomic environment, and cognitive development in the 1958 British birth cohort study. *BMJ* 2002;**325**:305. doi:10.1136/bmj.325.7359.305

28 Kramer MS, Aboud F, Mironova E, *et al.* Breastfeeding and Child Cognitive Development: New Evidence From a Large Randomized Trial. *Arch Gen Psychiatry* 2008;**65**:578–84. doi:10.1001/archpsyc.65.5.578

29 Walker SP, Wachs TD, Grantham-McGregor S, *et al.* Inequality in early childhood: risk and protective factors for early child development. *The Lancet* 2011;**378**:1325–38.

For peer review only - http://bmjopen.bmj.com/site/about/guidelines.xhtml

30 Murray GK, Jones PB, Kuh D, *et al.* Infant developmental milestones and subsequent cognitive function. *Ann Neurol* 2007;**62**:128–36. doi:10.1002/ana.21120

31 Kelly Y, Sacker A, Gray R, *et al.* Light drinking in pregnancy, a risk for behavioural problems and cognitive deficits at 3 years of age? *Int J Epidemiol* 2009;**38**:129–40. doi:10.1093/ije/dyn230

32 Dahlgren G, Whitehead M. Policies and strategies to promote social equity in health. *Stockh Inst Future Stud* 1991.

33 Condon JT, Corkindale CJ. The assessment of parent-to-infant attachment: development of a self-report questionnaire instrument. *J Reprod Infant Psychol* 1998;**16**:57–76.

34 Sosthenes C. Ketende, Elizabeth M. Jones. User Guide to Analysing MCS Data Using STATA. Centre for Longitudinal Studies, Institute of Education 2011.

35 Peduzzi P, Concato J, Feinstein AR, *et al.* Importance of events per independent variable in proportional hazards regression analysis II. Accuracy and precision of regression estimates. *J Clin Epidemiol* 1995;**48**:1503–1510.

36 Azen R, Traxel N. Using Dominance Analysis to Determine Predictor Importance in Logistic Regression. *J Educ Behav Stat* 2009;**34**:319–47. doi:10.3102/1076998609332754

37 Pencina MJ, D'Agostino RB, Pencina KM, *et al.* Interpreting Incremental Value of Markers Added to Risk Prediction Models. *Am J Epidemiol* 2012;**176**:473–81. doi:10.1093/aje/kws207

38 Windmeijer FAG. The asymptotic distribution of the sum of weighted squared residuals in binary choice models. *Stat Neerlandica* 1990;**44**:69–78.

39 Steyerberg EW, Harrell FE, Borsboom GJJM, *et al.* Internal validation of predictive models. *J Clin Epidemiol* 2001;**54**:774–81. doi:10.1016/S0895-4356(01)00341-9

40 White IR, Royston P, Wood AM. Multiple imputation using chained equations: Issues and guidance for practice. *Stat Med* 2011;**30**:377–99. doi:10.1002/sim.4067

41 Centre for Longitudinal Studies. MCS Ethical Review and Consent. London: : Institute of Education 2014. http://www.cls.ioe.ac.uk/shared/get-file.ashx?id=1601&itemtype=document

42 Pepe MS, Janes H, Longton G, *et al.* Limitations of the Odds Ratio in Gauging the Performance of a Diagnostic, Prognostic, or Screening Marker. *Am J Epidemiol* 2004;**159**:882–90. doi:10.1093/aje/kwh101

43 Hosmer DW, Hjort NL. Goodness-of-fit processes for logistic regression: simulation results. *Stat Med* 2002;**21**:2723–2738.

44 Wilkinson RG, Marmot M. *Social Determinants of Health: The Solid Facts*. World Health Organization 2003.

45 McKean C, Law J, Mensah F, *et al.* Predicting Meaningful Differences in School-Entry Language Skills from Child and Family Factors Measured at 12 months of Age. *Int J Early Child* 2016;**48**:329–51. doi:10.1007/s13158-016-0174-0

For peer review only - http://bmjopen.bmj.com/site/about/guidelines.xhtml

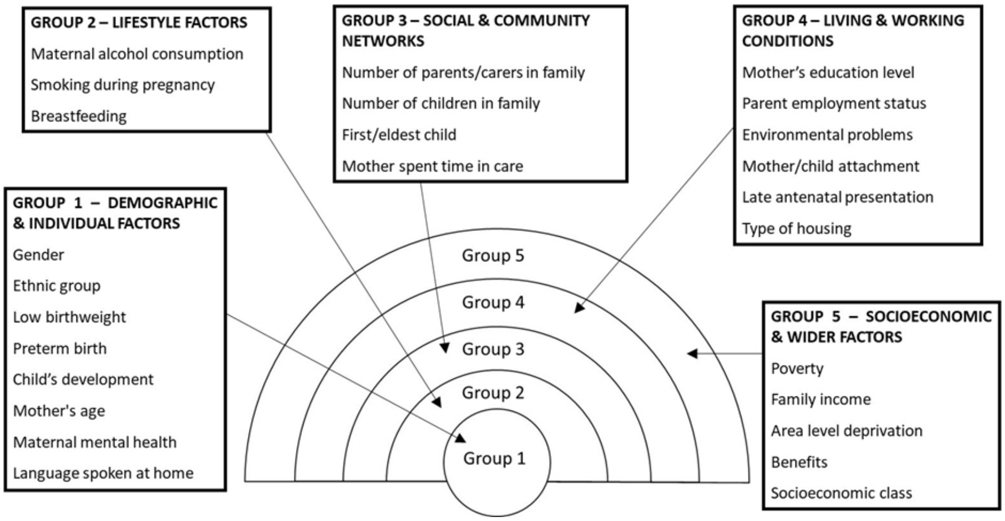

Figure 1 - Rainbow Model showing determinants of school readiness (adapted from Dahlgren and Whitehead, 1991)

99x50mm (240 x 240 DPI)

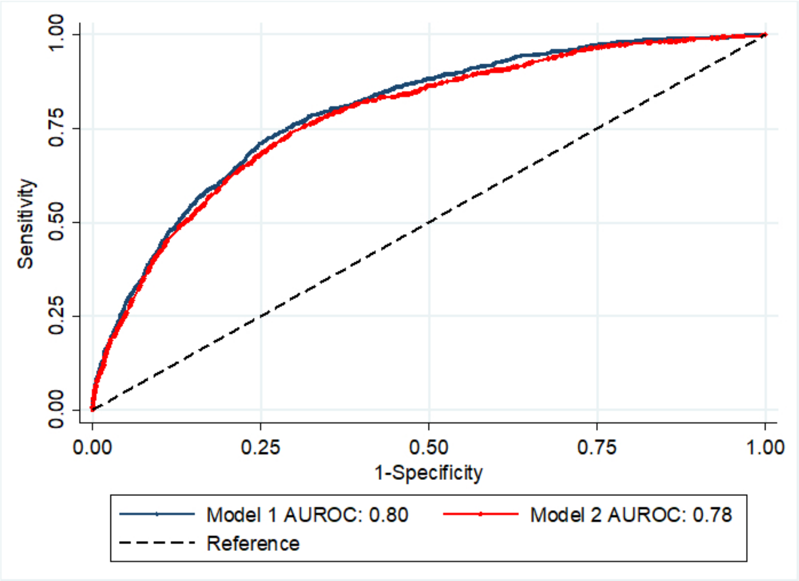

AUROC cut off points are: 0.9-1 = excellent, 0.8-<0.9 = good, 0.7-<0.8 = fair, 0.6-<0.7 = poor, 0.5-<0.6 = fail

Figure 2 - ROC curves for models 1 (13 predictors) and 2 (6 predictors), showing AUROC and IDI

92x59mm (240 x 240 DPI)

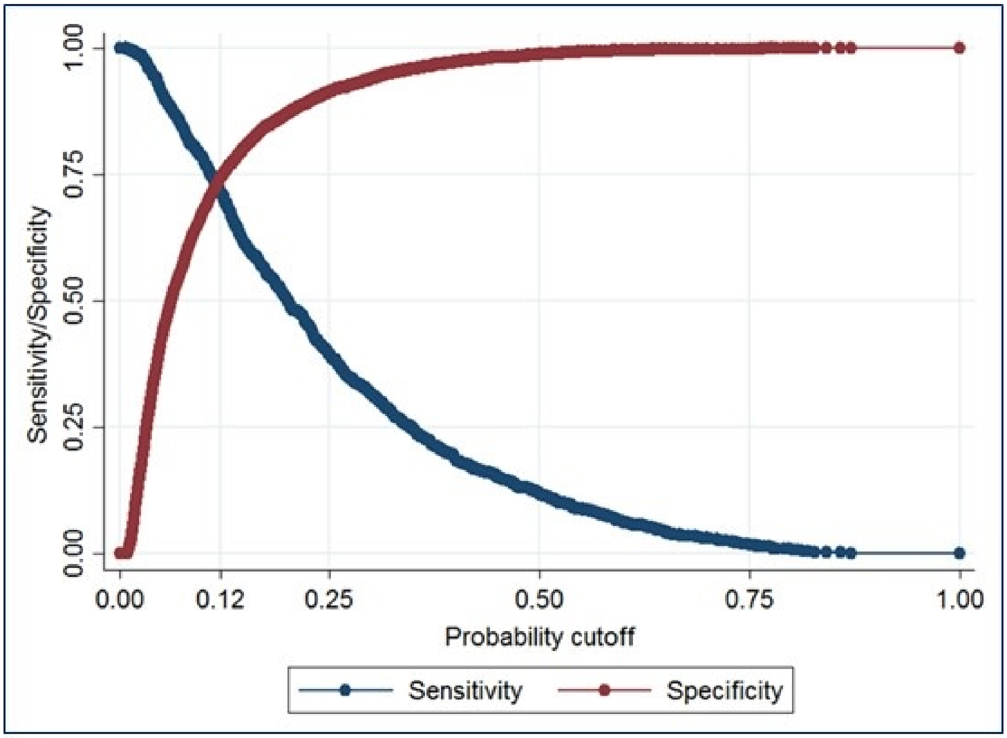

Figure 3 - Maximised probability cut off of sensitivity and specificity of model 1

58x43mm (240 x 240 DPI)

SUPPLEMENTARY FILE 1

Table 1 - Adjusted associations for the predictor variables in model 1 (13 predictors) using multiple imputed data (n=11,879)

Predictor	Adjusted OR (95% CI)
**GROUP 1 - DEMOGRAPHIC & INDIVIDUAL FACTORS**	
**Gender**	
Male	1
Female	0.47 (0.41-0.54)
**Ethnicity**	
White	1
Mixed	1.04 (0.62-1.75)
Indian	2.68 (1.85-3.89)
Pakistani and Bangladeshi	3.85 (2.94-5.04)
Black or Black British	2.31 (1.43-3.72)
Other ethnic group	3.95 (2.30-6.77)
**Mother's age at birth of first child**	
30-39	1
40+	1.05 (0.67-1.64)
20-29	1.22 (0.99-1.51)
14-19	1.22 (0.93-1.59)
**Birth weight (<2500grams)**	
Normal/high	1
Low birthweight	1.52 (1.18-1.97)
**Maternal Mental Health (Diagnosed depression/anxiety)**	
No	1
Yes	1.15 (0.98-1.34)
**Child developmental milestones**	
Above average	1
Average	1.29 (1.07-1.57)
Below average	1.60 (1.33-1.92)
**GROUP 2 - LIFESTYLE FACTORS**	
**Duration of breastfeeding**	
6 months or more	1
6 weeks - 6 months	1.17 (0.92-1.48)
One week or less	1.15 (0.90-1.48)
1 - 6 weeks	1.22 (0.96-1.57)
Never	1.58 (1.29-1.95)
**GROUP 3 - SOCIAL & COMMUNITY NETWORKS**	
**Number of children in family**	
One child	1
Two or three children	1.40 (1.19-1.63)
Four or more children	2.48 (1.94-3.16)
**GROUP 4 - LIVING & WORKING CONDITIONS**	

For peer review only

**Maternal education**	
Degree plus	1
Diploma	0.88 (0.61-1.26)
A-levels	1.13 (0.80-1.59)
GCSE A-C	1.34 (1.01-1.78)
GCSE D-G	1.72 (1.23-2.39)
None	1.74 (1.28-2.38)
**Workforce status**	
Both parents in work	1
One parent in work	0.94 (0.78-1.12)
Neither parent in work	1.21 (0.93-1.57)
**Housing tenure**	
Owner occupied	1
Private rented	1.18 (0.90-1.54)
Social housing	1.43 (1.18-1.72)
Other	0.96 (0.69-1.35)
**GROUP 5 - SOCIOECONOMIC AND WIDER FACTORS**	
**Social class**	
Managerial & professional	1
Intermediate	0.98 (0.75-1.29)
Small employers & own account	1.32 (0.87-2.00)
Lower supervisory & technical	1.50 (1.06-2.13)
Semi-routine & routine	1.77 (1.38-2.27)
Never worked & long-term unemployed	2.19 (1.53-3.15)
**Annual income**	
£33,000+	1
£22,000-£33,000	1.33 (1.02-1.72)
£11,000-£22,000	1.67 (1.30-2.14)
£0-£11,000	2.14 (1.60-2.87)
**ROC Analysis**	
**AUROC = 0.79 (95% CI 0.78 - 0.80)**	

For peer review only

SUPPLEMENTARY FILE 2

Results of integrated discrimination improvement (IDI) analysis, variables added according to their rank from the dominance analysis.

Variables included	IDI (%)	p	1-IDI
1	7.3%	<0.00001	92.7%
2	5.3%	<0.00001	94.7%
3	3.8%	<0.00001	96.2%
4	3.5%	<0.00001	96.5%
5	2.3%	<0.00001	97.7%
6	1.3%	<0.00001	98.7%
7	1.0%	<0.00001	99.0%
8	0.9%	<0.00001	99.1%
9	0.6%	0.00001	99.4%
10	0.6%	0.00001	99.4%
11	0.2%	0.01402	99.8%
12	0.0%	0.52356	100.0%

For peer review only

TRIPOD Checklist: Prediction Model Development

Section/Topic	Item	Checklist Item	Page
**Title and abstract**			
Title	1	Identify the study as developing and/or validating a multivariable prediction model, the target population, and the outcome to be predicted.	1
Abstract	2	Provide a summary of objectives, study design, setting, participants, sample size, predictors, outcome, statistical analysis, results, and conclusions.	2
**Introduction**			
Background and objectives	3a	Explain the medical context (including whether diagnostic or prognostic) and rationale for developing or validating the multivariable prediction model, including references to existing models.	3
	3b	Specify the objectives, including whether the study describes the development or validation of the model or both.	3
**Methods**			
Source of data	4a	Describe the study design or source of data (e.g., randomized trial, cohort, or registry data), separately for the development and validation data sets, if applicable.	3
	4b	Specify the key study dates, including start of accrual; end of accrual; and, if applicable, end of follow-up.	3
Participants	5a	Specify key elements of the study setting (e.g., primary care, secondary care, general population) including number and location of centres.	3-4
	5b	Describe eligibility criteria for participants.	4
	5c	Give details of treatments received, if relevant.	
Outcome	6a	Clearly define the outcome that is predicted by the prediction model, including how and when assessed.	4
	6b	Report any actions to blind assessment of the outcome to be predicted.	
Predictors	7a	Clearly define all predictors used in developing or validating the multivariable prediction model, including how and when they were measured.	4-5
	7b	Report any actions to blind assessment of predictors for the outcome and other predictors.	temporal ordering in cohort
Sample size	8	Explain how the study size was arrived at.	5
Missing data	9	Describe how missing data were handled (e.g., complete-case analysis, single imputation, multiple imputation) with details of any imputation method.	6
Statistical analysis methods	10a	Describe how predictors were handled in the analyses.	5-6
	10b	Specify type of model, all model-building procedures (including any predictor selection), and method for internal validation.	6
	10d	Specify all measures used to assess model performance and, if relevant, to compare multiple models.	6
Risk groups	11	Provide details on how risk groups were created, if done.	
**Results**			
Participants	13a	Describe the flow of participants through the study, including the number of participants with and without the outcome and, if applicable, a summary of the follow-up time. A diagram may be helpful.	6
	13b	Describe the characteristics of the participants (basic demographics, clinical features, available predictors), including the number of participants with missing data for predictors and outcome.	6-7
Model development	14a	Specify the number of participants and outcome events in each analysis.	10
	14b	If done, report the unadjusted association between each candidate predictor and outcome.	8-10
Model specification	15a	Present the full prediction model to allow predictions for individuals (i.e., all regression coefficients, and model intercept or baseline survival at a given time point).	
	15b	Explain how to the use the prediction model.	
Model performance	16	Report performance measures (with CIs) for the prediction model.	10
**Discussion**			
Limitations	18	Discuss any limitations of the study (such as nonrepresentative sample, few events per predictor, missing data).	11-12
Interpretation	19b	Give an overall interpretation of the results, considering objectives, limitations, and results from similar studies, and other relevant evidence.	11
Implications	20	Discuss the potential clinical use of the model and implications for future research.	11-13
**Other information**			
Supplementary information	21	Provide information about the availability of supplementary resources, such as study protocol, Web calculator, and data sets.	
Funding	22	Give the source of funding and the role of the funders for the present study.	

We recommend using the TRIPOD Checklist in conjunction with the TRIPOD Explanation and Elaboration document.

Development of a Predictive Risk Model for School Readiness at age 3 years using the UK Millennium Cohort Study

Journal:	*BMJ Open*
Manuscript ID	bmjopen-2018-024851.R1
Article Type:	Research
Date Submitted by the Author:	01-Feb-2019
Complete List of Authors:	Camacho, Christine; University of Liverpool Department of Public Health and Policy,<br>Straatmann, Viviane; University of Liverpool Department of Public Health and Policy; Karolinska Institutet, Ageing Research<br>Day, Jennie; University of Liverpool Department of Public Health and Policy<br>Taylor-Robinson, David; University of Liverpool, Department of Public Health and Policy
**Primary Subject Heading**:	Public health
Secondary Subject Heading:	Public health, Epidemiology, Health policy
Keywords:	PUBLIC HEALTH, EPIDEMIOLOGY, SOCIAL MEDICINE

SCHOLARONE™
Manuscripts

Development of a Predictive Risk Model for School Readiness at age 3 years using the UK Millennium Cohort Study

Miss CL Camacho*[1], Dr VS Straatmann[1,2], Dr JC Day[1], Prof DC Taylor-Robinson[1]

Affiliations: [1]*Public Health and Policy, University of Liverpool, Liverpool, UK;* [2]*Aging Research, Karolinska Institute, Stockholm, Sweden*

Address correspondence to: Christine Camacho, Department of Public Health and Policy, University of Liverpool, Liverpool, United Kingdom, L69 3GB, [c.camacho@nhs.net], +44 07973395585

For peer review only

For peer review only - http://bmjopen.bmj.com/site/about/guidelines.xhtml

ABSTRACT

Objectives

The aim of this study was to develop a predictive risk model (PRM) for school readiness measured at age 3 years using perinatal and early infancy data.

Design and Participants

This paper describes the development of a predictive risk model. Predictors were identified from the UK Millennium Cohort Study (MCS) wave 1 data, collected when participants were 9 months old. The outcome was school readiness at age 3 years, measured by the Bracken School Readiness Assessment. Stepwise selection and dominance analysis were used to specify 2 models. The models were compared by the area under the receiver operating characteristic curve (AUROC) and integrated discrimination improvement (IDI).

Results

Data were available for 9,487 complete cases. At age 3, 11.7% (95% CI 11.0-12.3%) of children were not school ready. The variables identified were: parents' Socio-Economic Classification, child's ethnicity, maternal education, income band, sex, household number of children, mother's age, low birth weight, mother's mental health, infant developmental milestones, breastfeeding, parents' employment, housing type. A parsimonious model included the first six listed variables (model 2). The AUROC for model 1 was 0.80 (95% CI 0.78-0.81) and 0.78 (95% CI 0.77-0.79) for model 2. Model 1 resulted in a small improvement in discrimination (IDI=1.3%, p<0.001).

Conclusions

Perinatal and infant risk factors predicted school readiness at age 3 with good discrimination. Social determinants were strong predictors of school readiness. This study demonstrates that school readiness can be predicted by six attributes collected around the time of birth.

For peer review only

For peer review only - http://bmjopen.bmj.com/site/about/guidelines.xhtml

Strengths and limitations of this study

- Use of a large, representative, and contemporary cohort study to demonstrate the feasibility of predicting school readiness from data collected in infancy.
- Multiple imputation and bootstrapping were used to evaluate the impact of missing data and internal validity, respectively.
- The main outcome measure, the Bracken School Readiness Assessment, was developed in the US, and is not routinely used in the UK.
- This model was not externally validated, which would have given an indication of generalisability.

INTRODUCTION

Early childhood is a critical time for lifelong physical, social, emotional and cognitive development. A wide range of factors are associated with early cognitive development (ECD)[1]. Interventions in the first three years of life can improve the trajectory of ECD[2] and deliver the greatest return on investment[3], yet it is unclear how best to identify children at most risk of delayed ECD, to enable appropriate targeting of interventions.

Cognitive development measures in children are good indicators of later educational achievement, predict health and social care needs in adults[4,5], and are associated with long term health outcomes[6]. There has been a growing policy interest in school readiness as a measure of ECD[7], and school readiness is a key public health indicator in children in the UK. Good school readiness lays a platform for future learning, employment and health[8,9].

School readiness is currently a major focus in England [10] and national metrics are collected to capture changes over time. In 2017, 29% of children in England were deemed not school ready at the end of their reception year (aged 4-5 years)[11]. There was nearly a 20% point gap in performance between the most (62% school ready) and the least (80%) deprived deciles of Index of Multiple Deprivation [12]. In UK policy there has been a focus on demographic factors e.g. maternal age, in targeting early interventions for children[13]. This study will explore the importance of different variables in predicting school readiness.

Previous research has identified a wide range of variables associated with early cognitive development. Predictive risk models (PRMs) are well-established in many clinical disciplines and have more recently been applied to child development. Using PRMs in this context could facilitate targeted early intervention as part of a proportionate universalism approach, which requires universal action with the scale and intensity of interventions proportionate to the level of need[6]. Most models thus far have shown fair or poor discrimination and there have been very few studies in the UK [14–18]. The aim of this study was to develop, for the first time, a PRM for school readiness measured at age 3 years using perinatal and early infancy data from the UK Millennium Cohort Study (MCS).

METHODS

For peer review only - http://bmjopen.bmj.com/site/about/guidelines.xhtml

Overview

Data from the MCS were used to explore the relationship between the outcome, school readiness, and 29 predictor variables using logistic regression analysis. Following univariable analysis to test for unadjusted associations, automated stepwise regression analyses were used to select variables for inclusion in the PRM. Dominance analysis was used to rank and weight included predictors, and integrated discrimination improvement (IDI) was calculated to assess the difference in performance between models. A receiver operator characteristic (ROC) curve was used to evaluate how well the model discriminated school readiness. The area under an ROC curve (AUROC) gives a measure of how well the regression model predicts school readiness at age 3. Traditionally accepted AUROC cut off points are: 0.9-1 = excellent, 0.8-<0.9 = good, 0.7-<0.8 = fair, 0.6-<0.7 = poor, 0.5-<0.6 = fail[19]. Multiple imputation was used to assess the impact of missing data in the sample.

Data Source

The PRM was developed and validated using MCS data. The MCS is a nationally representative birth cohort study which recruited 18,550 children born from September 2000 to January 2002, followed up in ongoing data collection waves. The sampling frame was government child benefit records, which had almost universal coverage at the time of sampling. The sample was clustered at the level of electoral ward and stratified to allow over representation of children living in deprived areas and areas with high concentrations of ethnic minorities[20]. Further information about the MCS sample is available in the cohort profile[21]. Data were collected from the main responder (usually mothers) by trained interviewers in participants' homes using a combination of interviews and self-completed questions. All singleton children in the first (aged 9 months) and second (aged 3 years) waves of the MCS with completed data for the outcome and predictors were eligible for inclusion (n=9,487).

Outcome

School readiness was measured using the Bracken School Readiness Assessment (BSRA) which consists of 6 subtests relating to colours, letters, numbers/counting, sizes, comparisons and shapes[22]. The assessment was carried out by interviewers during the second data collection wave when children were aged approximately 3 years old. The BSRA and its predecessors have demonstrated good reliability[23] and validity against other measures and teacher assessments[24].

The BSRA raw scores were summed and adjusted for age to provide a standardised composite score[22]. Scores were grouped according to cut-offs recommended by Bracken which reflected a 'normative classification' whereby children were categorised as very delayed, delayed, average, advanced or very advanced [25]. We used the same cut off score as Bracken (mean standardised composite score <85, 1 standard deviation below mean) but collapsed the categories of delayed or very delayed into a single category equivalent to not being school ready. We have dichotomised the outcome 'school readiness' in line with UK policy, and to allow the testing of a PRM using ROC analysis which requires a binary outcome [26].

For peer review only - http://bmjopen.bmj.com/site/about/guidelines.xhtml

Predictors

29 predictor variables were used, which were collected at age 9 months in the first wave of MCS data collection during which data relevant to pregnancy, birth and the perinatal period was captured retrospectively. These were identified from previous research to predict cognitive development and were included in the MCS[1,2,4,6,27–34]. The selected predictor variables were grouped according to the Dahlgren and Whitehead theoretical model[35] of social determinants of health as depicted in Figure 1. This model was chosen to provide a framework for categorising predictors to allow analysis of the determinants of early cognitive development.

<<Figure 1 here>>

Group 1 – Demographic and Individual factors

Demographic characteristics included child sex, maternal ethnicity, child weight, pre-term birth, mother's age, home language, maternal mental health and child development categorised as shown in Box 1.

Box 1 – Coding of Group 1 demographic and individual factors

Categorisation of Demographic and Individual factors
Child sex – 'female' and 'male'
Maternal ethnicity – 'white', 'mixed', 'Indian', 'Pakistani and Bangladeshi', 'Black' and 'other'
Child weight at birth – low (<2.5kg) or normal/high (≥2.5kg)
Preterm birth – gestation period less than 37 weeks
Mother's age in years at birth of first child – grouped into 4 categories (14-19, 20-29, 30-39, 40+ years)
Home language – 'English only', 'English and another language', 'another language only'
Mental health (1) – Sad or low for >2 weeks since baby, coded as 'yes' or 'no'
Mental health (2) – Diagnosis of depression or serious anxiety, coded as 'yes' or 'no'
Mental health (3) – 9-item modified version of the Rutter Malaise Inventory[39], coded as 'low' or (0-3) 'high' (4-9) scores[27].
Child development – 8 items from Denver Developmental Screening Test and 5 items from MacArthur Communicative Development Inventory, scored on a continuous scale from 13 (above average) to 36 (below average)

Group 2 – Lifestyle Factors

Self-reported maternal smoking was coded as 'never smoked', 'smoked before pregnancy' and 'smoked during pregnancy'. Maternal alcohol consumption during pregnancy were categorised as 'never or very infrequent', 'occasional', 'regularly' and 'most or everyday'. Breastfeeding duration was grouped as 'never', 'one week or less', '1 – 6 weeks', '6 weeks – 6 months' and 'over 6 months'.

Group 3 – Social and Community Factors

The number of children in household was coded as '1', '2-3' or '4+', and being the eldest or only child was recoded as 'yes' or 'no'. The number of parents or carers was either '1' or '2'. Mothers were asked how much time they had spent time in care before the age of 17, this was recoded as 'yes' or 'no' to indicate if they had ever been in care.

Group 4 – Living and Working Conditions

For peer review only - http://bmjopen.bmj.com/site/about/guidelines.xhtml

Maternal education was categorised into six groups ‘degree plus (higher degree and first degree qualifications)’, ‘diploma (in higher education)’, ‘A-levels’, ‘GCSE grades A–C’, ‘GCSE grades D–G’ and ‘none of these qualifications’. Parent’s employment status was classified as either ‘both’, ‘one’ or ‘neither’ parents in work[1]. Housing tenure was coded as ‘owner occupied’, ‘private rented’, ‘social housing’ and ‘other’. The response to the question, “How common is pollution, grime or other environmental problems?” was recoded as ‘common’, ‘not common’ and ‘not at all’. Presentation for first antenatal visit was recoded as late if after 12 weeks. Maternal attachment was measured using a 6-item Condon Maternal Attachment Questionnaire[36] grouped as ‘low (10-21), ‘average’ (22-23) and ‘high (24-27) .

Group 5 – Socioeconomic and Wider Factors
The National Statistics Socio-Economic Classification (NS-SEC) was used to code job details for main respondents (the majority of which were mothers) as: ‘managerial & professional’, ‘intermediate’, ‘small employers & own account’, ‘lower supervisory & technical’, ‘semi-routine & routine’, ‘never worked & long-term unemployed’. Net household income was reported by identification of the correct band on a show card and grouped into 4 quartile bands[27]: ‘£0-£11,000’, ‘£11,000-£22,000’, ‘£22,000-£33,000’ and ‘£33,000+’. Poverty was defined as an equivalised household income 60% below the median before housing costs according to the Organisation for Economic Co-operation and Development Household Equivalence Scale. Families reported receipt of any means-tested benefits, including Jobseekers Allowance, Income Support, Working Families Tax Credit or Disabled Persons Tax Credit. Indices of Multiple Deprivation (IMD) from 2004 were linked retrospectively to wave 1 data to give small area level deprivation measure. IMD scores were divided into quintiles, with 1 the most deprived quintile, and 5 the least deprived.

Statistical analyses
Analyses were conducted using Stata v14.2 (StataCorp LP, 2017). Survey weights were applied to take account of clustering, stratification and oversampling in the survey design, and attrition between survey waves, using the svyset command (pweight=BOVWT2) and svy prefix for regression modelling[37]. The number of events per variable (EPV) exceeds 35, the predictors were checked for collinearity, a large number of predictors were used and all were significantly associated with the outcome suggesting a robust logistic regression model with sufficient sample size [38,39].

Descriptive analysis of each predictor and school readiness was carried out to ascertain the prevalence of each predictor in the sample. Univariable logistic regression analyses calculating odds ratios (ORs) and 95% confidence intervals (95% CI) were carried out to assess the unadjusted association of each variable with the outcome.

A multivariable logistic regression model including all 29 variables was reduced using automated forward and backwards stepwise selection (using a cut off p-value of 0.1).. Dominance analysis (repeated regression analyses on subsets of variables) was used to produce

[1] Being on leave from work is classed as being in employment

For peer review only - http://bmjopen.bmj.com/site/about/guidelines.xhtml

a ranking and weighting for each predictor in model 1[40]. These rankings were used to specify a more parsimonious model (model 2) containing the top 6 predictors, selected to maximise parsimony and performance. The integrated discrimination improvement (IDI) using the complete case sample from model 1 was calculated to assess difference in performance between models as the percentage change in individuals being correctly assigned by the model[41].

The area under the ROC curve (AUROC) and its 95% CI was used to measure discriminatory power of the models. Classification, including sensitivity and specificity, was assessed at the maximised probability cut off point where the sensitivity and specificity curves intersected. Calibration of the model was assessed using the Pearson Chi-squared test[42]. Bootstrapping was used for internal validation; model performance was assessed using 1000 bootstrap samples, model optimism was averaged across all iterations to obtain an optimism estimate. An optimism-corrected AUROC, which takes account of overfitting, was calculated as the difference between unadjusted performance and the optimism estimate [43].

A complete case approach was used for the primary analysis. As a sensitivity analysis, multiple imputation by chained equation was performed to impute missing data (imputed sample, n=13,650). Variables from the first sweep and the outcome variable were used to shape the imputation of the missing data (maternal education, child's sex, mother's age at birth of first child and school readiness at age 3). Twenty imputed datasets were generated, and Rubin's rules were used to calculate results across the imputed datasets[44].

Robustness tests were carried out in which the final model was tested with an alternative outcome measure for early cognitive development (the British Ability Scales, also tested at age 3 in the MCS); different coding of outcome and predictor variables (e.g. maternal age as a continuous variable); and with the addition of another predictor variable (child care type at age 9 months). See supplementary file 1 for further details.

Ethics and Patient and public involvement

Ethical approval for each wave of the MCS was granted by NHS Multicentre Research Ethics Committees[45]. No further ethical approval was required for this secondary analysis of MCS data. There was no direct patient or public involvement in this analysis. However, the MCS has an ongoing programme of participant and public engagement.

RESULTS

There were 15,381 singleton children surveyed in MCS2, of which 13,650 had an outcome recorded for school readiness. Of these children 70% (n=9,487) had complete data for the outcomes and all the predictor variables. There were no significant differences in the characteristics of the imputed sample and the complete case sample (p value >0.05 for all chi-squared tests) (Table 1); results are reported for complete cases (see Supplementary file 2 for imputed sample results).

For peer review only - http://bmjopen.bmj.com/site/about/guidelines.xhtml

For peer review only

Table 1 - Description of perinatal, sociodemographic and economic characteristics by school ready of sample and imputed sample

	Complete Cases (n=9,487)		Imputed Data (n=13,650)	
**Is Child School Ready?**	**Yes (%)**	**No (%)**	**Yes (%)**	**No (%)**
All	88.3	11.7	85.5	14.5
**GROUP 1 - DEMOGRAPHIC & INDIVIDUAL FACTORS**				
**Gender**				
Female	91.6	8.4	89.4	10.6
Male	85.1	14.9	82.6	17.4
**Ethnicity**				
White	90.4	9.6	88.6	11.4
Mixed	91.1	8.9	84.7	15.3
Indian	79.3	20.7	78.1	21.9
Pakistani and Bangladeshi	55.7	44.3	56.3	43.7
Black or Black British	79.8	20.2	68	32
Other ethnic group	73.6	26.4	74.3	25.7
**Mother's age at birth of first child**				
14-19	78	22	76.4	23.6
20-29	87.9	12.1	86.1	13.9
30-39	95	5	94.4	5.6
40+	76.9	23.1	76	24
**Birth weight (<2500grams)**				
normal/high	88.8	11.2	86.1	13.9
low birthweight	80.2	19.8	77.7	22.3
**Maternal Mental Health (Diagnosed depression/anxiety)**				
No	89	11	86	14
Yes	86	14	84.4	15.6
**Child developmental milestones**				
Child development score (mean, 95%CI)	19.3 (19.2,19.3)	19.9 (19.7,20.1)	19.1 (19.0,19.1)	19.6 (19.4,19.7)
**GROUP 2 - LIFESTYLE FACTORS**				
**Duration of breastfeeding**				
6 months or more	92.5	7.5	90.5	9.5
6 weeks - 6 months	89.8	10.2	87.8	12.2
1 - 6 weeks	88.8	11.2	85.9	14.1
one week or less	88.8	11.2	86.4	13.6
Never	82.6	17.4	80	20
**GROUP 3 - SOCIAL & COMMUNITY NETWORKS**				
**Number of children in family**				
One child	92	8	89.1	10.9
Two or three children	87.7	12.3	85	15
Four or more children	71.7	28.3	70.2	29.8
**Maternal education**				
Degree plus	95.6	4.4	95.1	4.9
Diploma	94.6	5.4	93.9	6.1
A levels	92.7	7.3	92	8

For peer review only - http://bmjopen.bmj.com/site/about/guidelines.xhtml

GCSE A-C	88.5	11.5	87.4	12.6
GCSE D-G	81	19	79.1	20.9
None	71.3	28.7	69.2	30.8
**GROUP 4 - LIVING & WORKING CONDITIONS**				
**Workforce status**				
Both parents in work	92.6	7.4	91.6	8.4
One parent in work	85.8	14.2	83.4	16.6
Neither parent in work	68.5	31.5	70.1	29.9
**GROUP 5 - SOCIOECONOMIC AND WIDER FACTORS**				
**Housing tenure**				
Owner occupied	91.9	8.1	90.7	9.3
Private rented	83.8	16.2	80.5	19.5
Social housing	75.8	24.2	74.8	25.2
Other	83.4	16.6	81	19
**Social class**				
managerial & professional	95.5	4.5	94.6	5.4
intermediate	93.1	6.9	92.1	7.9
small employers & own account	91.3	8.7	89.1	10.9
lower supervisory & technical	87.2	12.8	84	16
semi-routine & routine	81.9	18.1	80	20
never worked & long-term unemployed	60.2	39.8	62.1	37.9
**Annual income**				
£33,000+	95.7	4.3	94.9	5.1
£22,000-£33,000	92.5	7.5	91.7	8.3
£11,000-£22,000	85	15	83.9	16.1
£0-£11,000	73.8	26.2	74.1	25.9

11.7% (95%CI 11.0-12.3%) of children aged 3 years were classified as not being school ready, but this varied significantly by the parents' ethnicity, maternal education and social class (Table 1). All 29 predictor variables were significantly associated with school readiness in univariable logistic regression analysis (p<0.1), so none were excluded at this stage.

The stepwise method reduced the final multivariable logistic regression model to 13 predictors: child's sex and ethnicity, mother's age at birth of first child, birthweight, maternal mental health, child development milestones, duration of breastfeeding, number of children in family, maternal education, parents' workforce status, housing tenure, social class and annual family income. In the adjusted analysis, Pakistani and Bangladeshi children were 4 times more likely to not be school ready than white children (OR 4.19 95% CI 3.14-5.58). The full results are shown in Table 2. There was no evidence of collinearity.

Table 2 - Unadjusted and adjusted associations and dominance analysis for the predictor variables in model 1 (13 predictors)

Predictors	Unadjusted OR (95% CI)	Adjusted OR (95% CI)	Weighting (rank)
**GROUP 1 - DEMOGRAPHIC & INDIVIDUAL FACTORS**			

For peer review only - http://bmjopen.bmj.com/site/about/guidelines.xhtml

For peer review only

**Gender**			
Female	1	1	9.5 (5)
Male	1.76 (1.54,2.01)	2.03 (1.72,2.39)	
**Ethnicity**			
White	1	1	14.7 (2)
Mixed	1.4 (0.96,2.04)	1.42 (0.78,2.58)	
Indian	1.85 (1.23,2.77)	2.58 (1.65,4.03)	
Pakistani and Bangladeshi	5.94 (4.82,7.32)	4.27 (3.20,5.69)	
Black or Black British	4.06 (2.90,5.69)	2.1 (1.13,3.88)	
Other ethnic group	2.33 (1.38,3.93)	2.92 (1.55,5.48)	
**Mother's age at birth of first child**			
30-39	1	1	2.9 (11)
40+	2.83 (2.29,3.49)	1.05 (0.68,1.63)	
20-29	5.57 (4.20,7.37)	1.28 (0.98,1.66)	
14-19	6.02 (4.84,7.48)	1.32 (0.95,1.83)	
**Birth weight (<2500grams)**			
Normal/high	1	1	1.4 (12)
Low birthweight	1.7 (1.34,2.16)	1.26 (0.92,1.72)	
**Maternal Mental Health (Diagnosed depression/anxiety)**			
No	1	1	0.4 (13)
Yes	1.33 (1.16,1.53)	1.28 (1.07,1.53)	
**Child developmental milestones**			
Developmental score	1.07 (1.05,1.10)	1.1 (1.07,1.14)	3.9 (11)
**GROUP 2 - LIFESTYLE FACTORS**			
**Duration of breastfeeding**			
6 months or more	1	1	3.9 (10)
6 weeks - 6 months	1.25 (1.02,1.53)	1.05 (0.81,1.36)	
One week or less	1.67 (1.34,2.09)	1.19 (0.89,1.59)	
1 - 6 weeks	1.68 (1.36,2.07)	1.25 (0.96,1.65)	
Never	2.74 (2.29,3.27)	1.49 (1.19,1.87)	
**GROUP 3 - SOCIAL & COMMUNITY NETWORKS**			
**Number of children in family**			
One child	1	1	7.8 (6)
Two or three children	1.44 (1.27,1.63)	1.38 (1.15,1.66)	
Four or more children	3.71 (3.04,4.54)	2.67 (1.94,3.68)	
**GROUP 4 - LIVING & WORKING CONDITIONS**			
**Maternal education**			
Degree plus	1	1	13.6 (3)
Diploma	1.3 (0.93,1.81)	0.81 (0.53,1.24)	
A levels	1.66 (1.22,2.25)	1.02 (0.68,1.55)	
GCSE A-C	3.02 (2.34,3.90)	1.3 (0.89,1.88)	
GCSE D-G	5.55 (4.21,7.30)	1.54 (1.02,2.34)	
None	9.62 (7.61,12.16)	1.68 (1.15,2.43)	
**Workforce status**			
Both parents in work	1	1	6.9 (7)
One parent in work	1.79 (1.49,2.14)	0.82 (0.67,1.00)	
Neither parent in work	5.39 (4.36,6.67)	1.21 (0.87,1.68)	

For peer review only - http://bmjopen.bmj.com/site/about/guidelines.xhtml

**Housing tenure**			
Owner occupied	1	1	
Private rented	2.68 (2.16,3.33)	1.21 (0.87,1.67)	5.7 (8)
Social housing	3.89 (3.34,4.53)	1.45 (1.16,1.81)	
Other	2.65 (2.10,3.35)	0.9 (0.62,1.30)	
**GROUP 5 - SOCIOECONOMIC AND WIDER FACTORS**			
**Social class**			
Managerial & professional	1	1	
Intermediate	1.5 (1.19,1.89)	1.06 (0.77,1.45)	
Small employers & own account	2.11 (1.44,3.08)	1.41 (0.87,2.28)	17.4 (1)
Lower supervisory & technical	3.72 (2.76,5.00)	1.65 (1.09,2.50)	
Semi-routine & routine	4.99 (4.13,6.01)	1.97 (1.46,2.66)	
Never worked & long-term unemployed	12.07 (9.48,15.37)	2.49 (1.69,3.66)	
**Annual income**			
£33,000+	1	1	
£22,000-£33,000	1.71 (1.31,2.25)	1.31 (0.96,1.79)	12.0 (4)
£11,000-£22,000	3.97 (3.12,5.07)	1.64 (1.22,2.22)	
£0-£11,000	7.7 (6.10,9.72)	2.26 (1.60,3.19)	

Dominance analysis showed that social class was the most important predictor (weighting=17.6), followed by ethnic group (weighting=14.7) and maternal education (weighting=13.8) (Table 2). Analysis of the predictor weightings suggests that social factors (average weighting 11.3, SD 4.9) are stronger predictors of school readiness than demographic and lifestyle factors (average weighting 5.5, SD 4.9). IDI was used to test the relative performance of models with all (1-13) variables, with variables added in according to their rank from the dominance analysis (Supplementary File 3). These analyses informed the specification of model 2, which was comprised of the top 6 predictors: social class, child's ethnic group, maternal education, income band, sex and number of children (see Supplementary File 4 for Model 2 results).

The AUROC was 0.80 (95% CI 0.78-0.81) for model 1 (n=9,487), which indicates a "good" level of discrimination[19]. The AUROC for model 2 (n=11,146) was 0.78 (95% CI 0.77-0.79). Internal validation using bootstrap optimism correction suggests that the model would have good discriminatory power in an independent sample (adjusted AUROC model 1 = 0.79, model 2=0.76). The Pearson Chi-squared tests were both non-significant indicating adequate calibration (model 1, p=0.07, model 2, p=0.13)[46]. IDI showed there was a small but significant difference in performance, with model 1 resulting in a 1.3% (p=<0.001) improvement in discrimination (Figure 2).

<<Figure 2 here>>

Sensitivity and specificity were plotted against probability cut-offs to select the optimal cut off point to assess the PRM's classification (model 1, cut-off=0.12; model 2, cut-off=0.14) (Figure 3**Error! Reference source not found.**). For model 1, at this cut-off point sensitivity was 72% (95% CI 69.0%-74.3%) and specificity was 74% (95% CI 73.5%-75.3%). Sensitivity of model 2 was similar - 72% (95% CI 69.9%-74.5%). Specificity was lower - 71% (95% CI 69.6%-

For peer review only - http://bmjopen.bmj.com/site/about/guidelines.xhtml

71.4%), so this model would generate more false positive results than the model 1, but performance was still in the acceptable range. At a probability cut-off of 12%, 31% of the screened population tested would be identified as being at high risk of poor school readiness using model 1.

<<Figure 3 here>>

A sensitivity analysis using an alternative outcome measure (British Ability Scales, BAS), showed that the BSRA measure led to improved discrimination (AUROC = 0.79 (95% CI 0.78-0.81) for BAS; AUROC = 0.80 (95% CI 0.78-0.81) for BSRA, p=0.002). See supplementary file 1 for further details.

For peer review only

DISCUSSION

Findings

This study developed a PRM for school readiness at age 3 years using perinatal and early childhood data from the MCS. Model 1 with 13 variables had good discrimination (AUROC=0.80) and classification (sensitivity=72%, specificity= 74% at a maximised cut off). Dominance analysis found the most important variables in predicting school readiness related to socioeconomic conditions (social class, maternal education, family income) and ethnicity. A parsimonious model performed similarly well (AUROC=0.78), suggesting it is possible to predict school readiness at age 3 using just six variables from the perinatal period and early infancy.

Comparison with previous studies

The value added of this study is that it is the first UK study to show that school readiness can be predicted with good discrimination with a small number of variables collected in infancy. The predictors of school readiness identified here corroborate previous findings. Male sex, maternal education, income, family composition, parental employment, housing and breastfeeding have been identified as significant risk factors of delayed ECD in other studies[4,14,15,17,18,27]. Social factors were the most important predictors, corresponding with current thinking on the social determinants of cognitive development[6,47].

The model reported here has good predictive strength, and compares favourably to similar PRMs, which with one exception[17], achieved only fair or poor discrimination[14,15,18,48]. Chittleborough et al used the ALSPAC UK birth cohort to test the predictive validity of 2 models for ECD[14]. They used a different outcome measure (School entry assessment aged 4-5) and used 6 predictors in their model, which appear to be chosen a priori, rather than by a statistical routine. They found that maternal age alone failed to predict ECD (AUROC~0.5), and a model with 6 predictors achieved only poor discrimination (AUROC=0.67). Camargo-Figuera et al used IQ as a measure of ECD and developed a PRM with 12 predictors using the Brazilian Pelotas birth cohort; their model had good discrimination (AUROC=0.8) and calibration, with sensitivity and specificity of 72% and 74% respectively[17]. We believe the use of a representative cohort for model development, stepwise regression to select predictor

For peer review only - http://bmjopen.bmj.com/site/about/guidelines.xhtml

variables and dominance analysis to specify a simplified model contributed to the good performance of this PRM.

Strengths and Limitations

A strength of this study was the use of a representative and contemporary UK cohort study as the data source. This offered a wide range of predictor variables and a large sample size which minimised the likelihood of overfitting. The cohort design also ensured correct temporal ordering and blinding with respect to the predictors. A theoretical model informed the PRM and statistical selection was used to specify variables. Multiple imputation was used to assess the impact of missing data. Bootstrapping showed good internal validity[49].

There are some limitations of this study to be considered. The main outcome, the BSRA, whilst validated as a measure of school readiness, was developed in the US and is not routinely used in the UK[24]. The BSRA measures a small set of pre-academic skills, but an analysis of MCS data linked to teacher reports showed that Bracken scores are strongly associated with the EYFS measure of school readiness used in English schools [4]. Many variables were dichotomised or grouped, which may be less sensitive than continuous measures. Longitudinal studies are subject to attrition and non-response which can introduce attrition bias, the use of survey weights partially adjust for this, but it was not possible to use these when calculating the AUROC. Sensitivity analysis using multiple imputation showed the effect of missing data was negligible, similar to other PRMs[14,15]. Most of the predictor variables were based on maternal self-report which may be subject to recall bias, and external validation was not conducted. The predictor variables identified may not be causally associated with school readiness and there are other predictors which may be associated with the outcome which were not included in this model e.g. childcare in infancy[50].

Policy Implications

The existing literature, and these findings, indicate that a PRM could plausibly be used to identify a group of children at high risk of poor ECD who may benefit from early intervention. If implemented as part of a "proportionate universalism" approach[6], PRMs could mitigate socioeconomic inequalities by providing early years settings with a mechanism for directing their resources to those children at highest risk of poor cognitive development. With new child and maternity datasets now being collected electronically in England, it may be possible to apply a PRM at population level through the use of linked administrative datasets as has been done in Australia[15].

Poor cognitive development is associated with a range of negative health and social outcomes and contributes to inequalities in society[3,5,6], so this is of public health importance. Chittleborough et al showed that even a model with poor discrimination has benefits over just using young maternal age to direct resources[14]. Similarly, McKean et al established that their PRM was better than existing clinical tools used to identify higher-risk children for early intervention[48].

The practical implications of using such a PRM as a screening tool should be considered. The model reported here would identify 31% of children screened as being 'at risk' of delayed

For peer review only - http://bmjopen.bmj.com/site/about/guidelines.xhtml

school readiness. An exemplar English Local Authority with a total population of 230,000, and 3000 children aged under 1 year would identify 900 ‘at risk’ children per year if the PRM was applied to this cohort. This percentage equates with national data; in 2015/16, 31% of children in England were not school ready when tested at age 4-5[11]. However, the overall accuracy of the model is 74%, so over 200 children would be incorrectly classified; this could lead to stigmatisation of families and unnecessary use of resources. Nelson et al (2016) comment that Early Intervention services would be overwhelmed by the level of demand generated by such PRMs[18]. A criterion for screening programmes is that interventions should be available, it is thus important to further consider the implications of using a PRM to assess ECD in the context of available resources.

Further research is needed to test the external validity of predictive risk models for ECD for example in another cohort or with linked administrative datasets. PRMs raise ethical issues; labelling very young children as being at risk of poor development could be stigmatising for families, particularly when social factors are the strongest predictors as in this analysis. PRMs would generate false positives (and false negatives), which could cause unnecessary distress. Use of PRMs to identify children at risk of developmental delay should include support and counselling for families, as well as timely access to appropriate interventions. Investment in early intervention would be required, which would have opportunity costs for services locally.

CONCLUSION

This study has identified a set of predictive risk factors from the perinatal period and early infancy that can predict school readiness at age 3 with a good level of accuracy. Poor cognitive development is socially patterned, evident from a very young age and leads to persistent disadvantage throughout life. It is possible that PRMs could be used to identify high risk children and target appropriate interventions and resources to improve their developmental trajectories, and to reduce social inequalities early in the life course.

STATEMENTS

Funding

This research received no specific grant from any funding agency in the public, commercial or not for profit sectors. DTR is funded by the MRC on a Clinician Scientist Fellowship (MR/P008577/1).

Competing Interests

We confirm that authors have no conflicts of interest to disclose.

Contributors

For peer review only - http://bmjopen.bmj.com/site/about/guidelines.xhtml

CLC, JCD and DTR planned the study. CLC and VSS conducted the analysis under the supervision of DTR. CLC led the drafting of the manuscript. All authors contributed to data interpretation, manuscript drafting and revisions and agreed the submitted version of the manuscript.

Data Sharing

The Millennium Cohort Study dataset is available from the UK Data Service.

ACKNOWLEDGEMENTS

We would like to acknowledge all the families and researchers who are part of the UK Millennium Cohort Study, without whom this research would not have been possible.

FIGURE CAPTIONS

Figure 1 - Rainbow Model showing determinants of school readiness (adapted from Dahlgren and Whitehead, 1991)

Figure 2 - ROC curves for models 1 (13 predictors) and 2 (6 predictors), showing AUROC and IDI

Figure 3 - Maximized probability cut off of sensitivity and specificity of model 1

For peer review only

For peer review only - http://bmjopen.bmj.com/site/about/guidelines.xhtml

REFERENCES

1 National Research Council (US) and Institute of Medicine (US) Committee on Integrating the Science of Early Childhood Development. *From Neurons to Neighborhoods: The Science of Early Childhood Development*. Washington (DC): : National Academies Press (US) 2000. http://www.ncbi.nlm.nih.gov/books/NBK225557/

2 Black MM, Walker SP, Fernald LC, *et al.* Early childhood development coming of age: science through the life course. *The Lancet* 2016.

3 Heckman JJ. Skill formation and the economics of investing in disadvantaged children. *Science* 2006;**312**:1900–2. doi:312/5782/1900 [pii]

4 Hobcraft JN, Kiernan KE. Predictive factors from age 3 and infancy for poor child outcomes at age 5 relating to children's development, behaviour and health: evidence from the Millennium Cohort Study. York: : University of York 2010. http://www.york.ac.uk/media/spsw/documents/research-and-publications/HobcraftKiernan2010PredictiveFactorsChildrensDevelopmentMillenniumCohort.pdf (accessed 28 May 2017).

5 Caspi A, Houts RM, Belsky DW, *et al.* Childhood forecasting of a small segment of the population with large economic burden. *Nat Hum Behav* 2016;**1**:0005.

6 Marmot M, Allen J, Goldblatt P, *et al.* Fair society, healthy lives: strategic review of health inequalities in England post 2010. Marmot Review Team 2010.

7 Public Health England. Improving school readiness: creating a better start for London. London: 2015.

8 Pia Rebello Britto. School Readiness - A conceptual framework. New York, NY: : UNICEF 2012.

9 Marmot M, Friel S, Bell R, *et al.* Closing the gap in a generation: health equity through action on the social determinants of health. *The Lancet* 2008;**372**:1661–9. doi:10.1016/S0140-6736(08)61690-6

10 Abreu L, Roberts N. Children's early years development and school readiness. 2016. https://researchbriefings.parliament.uk/ResearchBriefing/Summary/CDP-2016-0141 (accessed 30 Jan 2019).

11 Public Health England. Public Health Profiles. 2017.https://fingertips.phe.org.uk/profile-group/child-health (accessed 13 Jun 2017).

12 Early years foundation stage profile results: 2017 to 2018. GOV.UK. https://www.gov.uk/government/statistics/early-years-foundation-stage-profile-results-2017-to-2018 (accessed 22 Jan 2019).

13 FPH, FNP. Family Nurse Partnership. Faculty of Public Health 20102015.

For peer review only - http://bmjopen.bmj.com/site/about/guidelines.xhtml

14 Chittleborough CR, Lawlor DA, Lynch JW. Young Maternal Age and Poor Child Development: Predictive Validity From a Birth Cohort. *Pediatrics* 2011;**127**:e1436–44. doi:10.1542/peds.2010-3222

15 Chittleborough CR, Searle AK, Smithers LG, *et al.* How well can poor child development be predicted from early life characteristics?: A whole-of-population data linkage study. *Early Child Res Q* 2016;**35**:19–30.

16 Brownell MD, Ekuma O, Nickel NC, *et al.* A population-based analysis of factors that predict early language and cognitive development. *Early Child Res Q* 2016;**35**:6–18.

17 Camargo-Figuera FA, Barros AJ, Santos IS, *et al.* Early life determinants of low IQ at age 6 in children from the 2004 Pelotas Birth Cohort: a predictive approach. *BMC Pediatr* 2014;**14**:308. doi:10.1186/s12887-014-0308-1

18 Nelson BB, Dudovitz RN, Coker TR, *et al.* Predictors of Poor School Readiness in Children Without Developmental Delay at Age 2. *Pediatrics* 2016;**138**:e20154477. doi:10.1542/peds.2015-4477

19 Pepe MS, Janes H, Longton G, *et al.* Limitations of the Odds Ratio in Gauging the Performance of a Diagnostic, Prognostic, or Screening Marker. *Am J Epidemiol* 2004;**159**:882–90. doi:10.1093/aje/kwh101

20 Kirstine Hansen. Millennium Cohort Study First, Second, Third and Fourth Surveys. A Guide to the Datasets (Seventh Edition). London: : Centre for Longitudinal Studies 2012.

21 Connelly R, Platt L. Cohort Profile: UK Millennium Cohort Study (MCS). *Int J Epidemiol* 2014;**43**:1719–25. doi:10.1093/ije/dyu001

22 Kirstine Hansen. Millennium Cohort Study. First, Second, Third and Fourth Surveys. A Guide to the Datasets. Centre for Longitudinal Studies 2012. http://www.cls.ioe.ac.uk/shared/get-file.ashx?id=598&itemtype=document

23 Bracken B. Bracken Basic Concept Scale–Revised. San Antonio, TX: : The Psychological Corporation 1998.

24 Panter JE, Bracken BA. Validity of the Bracken School Readiness Assessment for predicting first grade readiness. *Psychol Sch* 2009;**46**:397–409.

25 Connelly R. Millennium Cohort Study Data Note 2013/1: Interpreting Test Scores. London: : Centre for Longitudinal Studies, Institute of Education 2013.

26 Steyerberg E. *Clinical prediction models: a practical approach to development, validation, and updating*. Springer Science & Business Media 2008.

27 Kiernan KE, Mensah FK. Maternal indicators in pregnancy and children's infancy that signal future outcomes for children's development, behaviour and health: evidence from the Millennium Cohort Study. York: : University of York 2010.

28 Kiernan KE, Huerta MC. Economic deprivation, maternal depression, parenting and children's cognitive and emotional development in early childhood. *Br J Sociol* 2008;**59**:783–806.

For peer review only - http://bmjopen.bmj.com/site/about/guidelines.xhtml

29 Shenkin SD, Starr JM, Deary IJ. Birth weight and cognitive ability in childhood: a systematic review. *Psychol Bull* 2004;**130**:989.

30 Jefferis BJMH, Power C, Hertzman C. Birth weight, childhood socioeconomic environment, and cognitive development in the 1958 British birth cohort study. *BMJ* 2002;**325**:305. doi:10.1136/bmj.325.7359.305

31 Kramer MS, Aboud F, Mironova E, *et al.* Breastfeeding and Child Cognitive Development: New Evidence From a Large Randomized Trial. *Arch Gen Psychiatry* 2008;**65**:578–84. doi:10.1001/archpsyc.65.5.578

32 Walker SP, Wachs TD, Grantham-McGregor S, *et al.* Inequality in early childhood: risk and protective factors for early child development. *The Lancet* 2011;**378**:1325–38.

33 Murray GK, Jones PB, Kuh D, *et al.* Infant developmental milestones and subsequent cognitive function. *Ann Neurol* 2007;**62**:128–36. doi:10.1002/ana.21120

34 Kelly Y, Sacker A, Gray R, *et al.* Light drinking in pregnancy, a risk for behavioural problems and cognitive deficits at 3 years of age? *Int J Epidemiol* 2009;**38**:129–40. doi:10.1093/ije/dyn230

35 Dahlgren G, Whitehead M. Policies and strategies to promote social equity in health. *Stockh Inst Future Stud* 1991.

36 Condon JT, Corkindale CJ. The assessment of parent-to-infant attachment: development of a self-report questionnaire instrument. *J Reprod Infant Psychol* 1998;**16**:57–76.

37 Sosthenes C. Ketende, Elizabeth M. Jones. User Guide to Analysing MCS Data Using STATA. Centre for Longitudinal Studies, Institute of Education 2011.

38 Peduzzi P, Concato J, Kemper E, *et al.* A simulation study of the number of events per variable in logistic regression analysis. *J Clin Epidemiol* 1996;**49**:1373–9. doi:10.1016/S0895-4356(96)00236-3

39 Courvoisier DS, Combescure C, Agoritsas T, *et al.* Performance of logistic regression modeling: beyond the number of events per variable, the role of data structure. *J Clin Epidemiol* 2011;**64**:993–1000.

40 Azen R, Traxel N. Using Dominance Analysis to Determine Predictor Importance in Logistic Regression. *J Educ Behav Stat* 2009;**34**:319–47. doi:10.3102/1076998609332754

41 Pencina MJ, D'Agostino RB, Pencina KM, *et al.* Interpreting Incremental Value of Markers Added to Risk Prediction Models. *Am J Epidemiol* 2012;**176**:473–81. doi:10.1093/aje/kws207

42 Windmeijer FAG. The asymptotic distribution of the sum of weighted squared residuals in binary choice models. *Stat Neerlandica* 1990;**44**:69–78.

43 Austin PC, Steyerberg EW. Events per variable (EPV) and the relative performance of different strategies for estimating the out-of-sample validity of logistic regression models. *Stat Methods Med Res* 2017;**26**:796–808.

For peer review only - http://bmjopen.bmj.com/site/about/guidelines.xhtml

44 White IR, Royston P, Wood AM. Multiple imputation using chained equations: Issues and guidance for practice. *Stat Med* 2011;**30**:377–99. doi:10.1002/sim.4067

45 Centre for Longitudinal Studies. MCS Ethical Review and Consent. London: : Institute of Education 2014. http://www.cls.ioe.ac.uk/shared/get-file.ashx?id=1601&itemtype=document

46 Hosmer DW, Hjort NL. Goodness-of-fit processes for logistic regression: simulation results. *Stat Med* 2002;**21**:2723–2738.

47 Wilkinson RG, Marmot M. *Social Determinants of Health: The Solid Facts*. World Health Organization 2003.

48 McKean C, Law J, Mensah F, *et al.* Predicting Meaningful Differences in School-Entry Language Skills from Child and Family Factors Measured at 12 months of Age. *Int J Early Child* 2016;**48**:329–51. doi:10.1007/s13158-016-0174-0

49 Steyerberg EW, Harrell FE, Borsboom GJJM, *et al.* Internal validation of predictive models. *J Clin Epidemiol* 2001;**54**:774–81. doi:10.1016/S0895-4356(01)00341-9

50 Côté SM, Doyle O, Petitclerc A, *et al.* Child Care in Infancy and Cognitive Performance Until Middle Childhood in the Millennium Cohort Study. *Child Dev* 2013;**84**:1191–208. doi:10.1111/cdev.12049

For peer review only

For peer review only - http://bmjopen.bmj.com/site/about/guidelines.xhtml

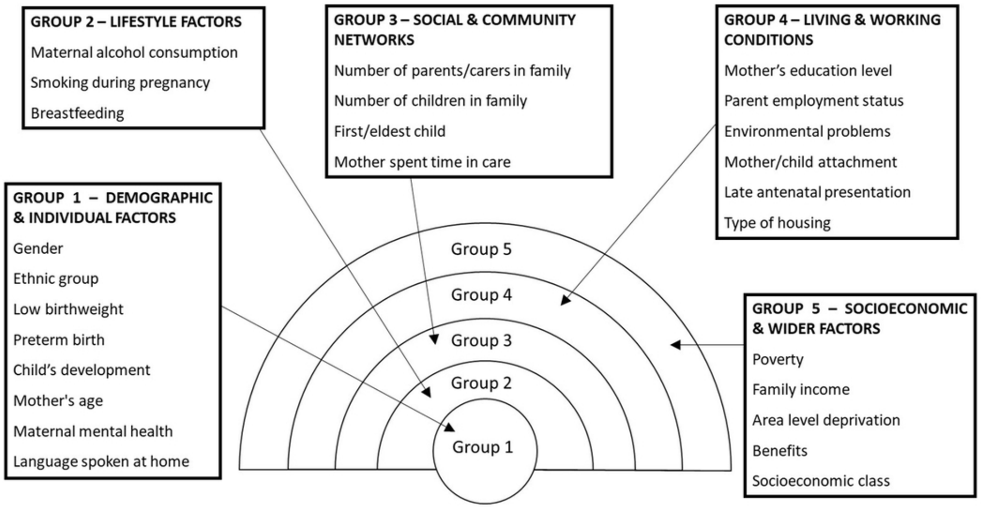

Figure 1 - Rainbow Model showing determinants of school readiness (adapted from Dahlgren and Whitehead, 1991)

90x50mm (300 x 300 DPI)

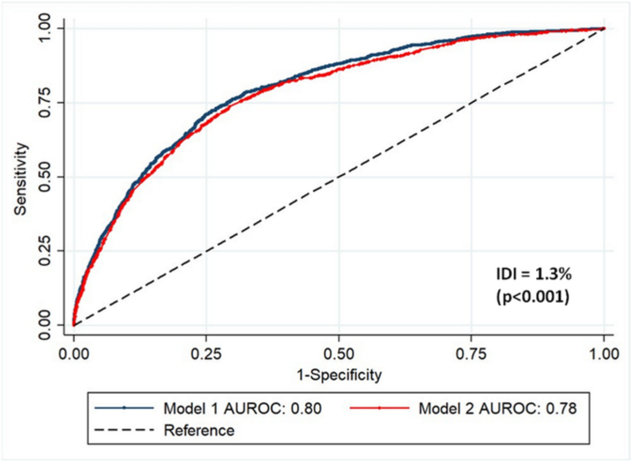

AUROC cut off points are: 0.9-1 = excellent, 0.8-<0.9 = good, 0.7-<0.8 = fair, 0.6-<0.7 = poor, 0.5-<0.6 = fail

Figure 2 - ROC curves for models 1 (13 predictors) and 2 (6 predictors), showing AUROC and IDI

90x50mm (300 x 300 DPI)

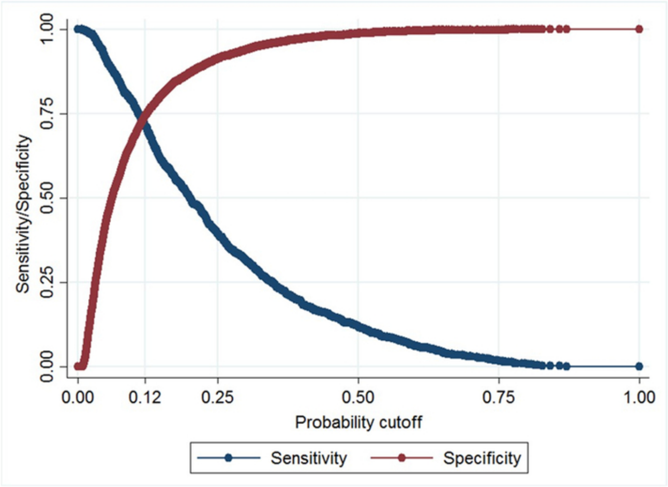

Figure 3 - Maximized probability cut off of sensitivity and specificity of model 1

90x50mm (300 x 300 DPI)

SUPPLEMENTARY FILE 1

Robustness tests were carried out in which the final model was tested with an alternative outcome measure for early cognitive development (British Ability Scales), different coding of variables and the addition of another predictor variable (child care type at age 9 months).

1. Using BAS as an alternative outcome variable

   An alternative measure of early cognitive development contained in the MSC are the British Ability Scales (BAS), measured at age 3. BAS scores were dichotomised to 1 SD below the mean as cut off for 'fail'. There is a moderate positive correlation between BAS and BSRA scores (r=0.5722, p<0.0001). The table below compares performance of the models; there is a small but statistically significant improvement in discrimination using BSRA as an outcome measure compared to BAS.

Outcome variable	N	AUROC (95% CI)
BSRA	9487	0.80 (0.78,0.81)
BAS	9487	0.79 (0.77,0.80)

Ho: area(xb1) = area(xb6); chi2(1) = 9.20, Prob>chi2 = 0.002

2. Robustness tests of the BSRA outcome measure

   The BSRA cut off used in the main analysis was a mean standardised composite score <85, which is 1 standard deviation below the mean. The standardisation sample was from a US population. As the BSRA has not been validated in the UK, we tested the model using dichotomised percentile ranks instead of MSCS as the outcome variable (cut off point 1 SD below mean).

   There was no significant different in model performance (AUROC=0.80 for both models, p=0.43). There is evidence to suggest that within the Millennium Cohort Study percentile scores can be misleading in indicating the difference between the performance of cohort members because they are on an ordinal, rather than interval, scale. An outcome based on MSCS was therefore retained.

3. Coding of predictor variables

   As a sensitivity analysis the coding of 4 predictor variables was altered: maternal age (from categorical to continuous), developmental scores (from categorical to continuous) and ethnicity (from categorical to binary). The impact of this on final model performance is shown below:

Description	n	AUROC	Comparative AUROC (n=9310)
Final model	9487	0.80	0.79 (0.77,0.81)
Developmental score (continuous)	9487	0.80	0.80 (0.78,0.81)
Maternal age (continuous)	9310	0.79	0.79 (0.78,0.81)
Ethnicity (binary)	9487	0.79	0.79 (0.78,0.80)

Ho: area(xb1) = area(xb2) = area(xb3) = area(xb4); chi2(3) = 9.98; Prob>chi2 = 0.02

In summary, there were small but statistically significant differences between the models. The only change which improved model discrimination was using continuous development

For peer review only

scores, so this was incorporated into the final model. There is a U-shaped relationship between school readiness and maternal age, so there was a clear rationale for including this as a categorical predictor.

4. Testing the impact of an additional predictor

There are other measures in the MCS which could have been used as predictors in this analysis. We have done a sensitivity analysis adding childcare type at 9 months to the final model. This reduces the overall discrimination of the model (AUROC = 0.77 vs 0.80), however this could be due to missing data as the child care variable is less complete. There is a statistically significant association with school readiness and child care type in the multivariable model, with children in formal child care settings more likely to be school ready than those being looked after by parents (OR = 1.76, p=0.02)

The Stata Do file for all analyses is available at: https://www.dropbox.com/s/zxsl4cl87imydp0/SchoolreadinessPRM.do?dl=0

For peer review only

SUPPLEMENTARY FILE 2

Table 1 - Adjusted associations for the predictor variables in model 1 (13 predictors) using multiple imputed data (n=11,879)

Predictor	Adjusted OR (95% CI)	Weighting (rank)
**GROUP 1 - DEMOGRAPHIC & INDIVIDUAL FACTORS**		
**Gender**		8.5 (5)
Female	1	
Male	1.86 (1.62,2.14)	
**Ethnicity**		15.7 (3)
White	1	
Mixed	1.04 (0.62,1.75)	
Indian	2.68 (1.85,3.89)	
Pakistani and Bangladeshi	3.85 (2.94,5.04)	
Black or Black British	2.31 (1.43,3.72)	
Other ethnic group	3.95 (2.30,6.77)	
**Mother's age at birth of first child**		1.5 (12)
30-39	1	
40+	1.05 (0.67,1.64)	
20-29	1.22 (0.99,1.51)	
14-19	1.22 (0.93,1.59)	
**Birth weight (<2500grams)**		1.2 (13)
Normal/high	1	
Low birthweight	1.52 (1.18,1.97)	
**Maternal Mental Health (Diagnosed depression/anxiety)**		1.5 (11)
No	1	
Yes	1.15 (0.98,1.34)	
**Child developmental milestones**		
Developmental score	1.10 (1.07,1.13)	2.8 (10)
**GROUP 2 - LIFESTYLE FACTORS**		
**Duration of breastfeeding**		3.6 (9)
6 months or more	1	
6 weeks - 6 months	1.17 (0.92,1.48)	
One week or less	1.15 (0.90,1.48)	
1 - 6 weeks	1.22 (0.96,1.57)	
Never	1.58 (1.29,1.95)	
**GROUP 3 - SOCIAL & COMMUNITY NETWORKS**		
**Number of children in family**		7.1 (6)
One child	1	
Two or three children	1.40 (1.19,1.63)	
Four or more children	2.48 (1.94,3.16)	
**GROUP 4 - LIVING & WORKING CONDITIONS**		
**Maternal education**		16.7 (2)
Degree plus	1	

Diploma	0.88 (0.61,1.26)	
A levels	1.13 (0.80,1.59)	
GCSE A-C	1.34 (1.01,1.78)	
GCSE D-G	1.72 (1.23,2.39)	
None	1.74 (1.28,2.38)	
**Workforce status**		
Both parents in work	1	6.5 (7)
One parent in work	0.94 (0.78,1.12)	
Neither parent in work	1.21 (0.93,1.57)	
**Housing tenure**		
Owner occupied	1	5.5 (8)
Private rented	1.18 (0.90,1.54)	
Social housing	1.43 (1.18,1.72)	
Other	0.96 (0.69,1.35)	
**GROUP 5 - SOCIOECONOMIC AND WIDER FACTORS**		
**Social class**		17.6 (1)
Managerial & professional	1	
Intermediate	0.98 (0.75,1.29)	
Small employers & own account	1.32 (0.87,2.00)	
Lower supervisory & technical	1.50 (1.06,2.13)	
Semi-routine & routine	1.77 (1.38,2.27)	
Never worked & long-term unemployed	2.19 (1.53,3.15)	
**Annual income**		11.9 (4)
£33,000+	1	
£22,000-£33,000	1.33 (1.02,1.72)	
£11,000-£22,000	1.67 (1.30,2.14)	
£0-£11,000	2.14 (1.60,2.87)	
**ROC Analysis**	**AUROC = 0.79 (95% CI 0.78,0.80)**	

For peer review only

SUPPLEMENTARY FILE 3

Integrated discrimination improvement (IDI) analysis was run using Stata function ‘idi’, which compares the discrimination ability between two logistic regression prediction models. In the first stage of this analysis, the IDI of a PRM with just the strongest predictor variable (social class) was compared to a model with all 13 predictors. Adding the additional 12 predictors lead to a 7.3% increase in IDI. In each subsequent analysis, an additional predictor variable was added according to the ranking of variables from the dominance analysis (Table 1).

Predictor	Weighting	Rank
Social Class	17.38	1
Ethnic group	14.66	2
Maternal education	13.55	3
Income band	12	4
Gender	9.54	5
Number of children	7.84	6
Parent's employment	6.9	7
Housing type	5.65	8
Child development	3.9	9
Breastfeeding	3.9	10
Mother's age at birth of first child	2.87	11
Low birth weight	1.42	12
Mental health	0.38	13

Table 1 - Results of the dominance analysis for model 1

The full results of integrated discrimination improvement (IDI) analysis are shown in Table 2.

Variables included	IDI (%)	p	1-IDI
1	7.3%	<0.00001	92.7%
2	5.3%	<0.00001	94.7%
3	3.8%	<0.00001	96.2%
4	3.5%	<0.00001	96.5%
5	2.3%	<0.00001	97.7%
6	1.3%	<0.00001	98.7%
7	1.0%	<0.00001	99.0%
8	0.9%	<0.00001	99.1%
9	0.6%	0.00001	99.4%
10	0.6%	0.00001	99.4%
11	0.2%	0.01402	99.8%
12	0.0%	0.52356	100.0%

Table 2 - Results of integrated discrimination improvement analysis for 12 models

A 6-predictor model was chosen as this offered the optimal balance between parsimony and discrimination.

For peer review only

SUPPLEMENTARY FILE 4

Table 1 - Adjusted associations for the predictor variables in model 2 (6 predictors) using complete cases (n=11,146) and multiple imputed data (n=11,879). The weightings and rank are from dominance analysis of the complete case sample.

Predictors	Adjusted OR (95% CI) - complete case	Adjusted OR (95% CI) - multiple imputation	Weighting (rank)
**GROUP 1 - DEMOGRAPHIC & INDIVIDUAL FACTORS**			
**Gender**			
Female	1	1	9.9 (5)
Male	1.99 (1.72,2.31)	1.93 (1.68,2.22)	
**Ethnicity**			
White	1	1	13.7 (4)
Mixed	1.2 (0.77,1.88)	1.26 (0.83,1.90)	
Indian	1.64 (1.09,2.47)	1.72 (1.14,2.59)	
Pakistani and Bangladeshi	2.67 (2.10,3.41)	2.71 (2.11,3.47)	
Black or Black British	2.32 (1.52,3.54)	2.69 (1.80,4.02)	
Other ethnic group	1.98 (1.10,3.58)	2.06 (1.27,3.32)	
**GROUP 3 - SOCIAL & COMMUNITY NETWORKS**			
**Number of children in family**			
One child	1	1	9.5 (6)
Two or three children	1.48 (1.27,1.73)	1.45 (1.25,1.69)	
Four or more children	2.89 (2.23,3.75)	2.62 (2.03,3.38)	
**GROUP 4 - LIVING & WORKING CONDITIONS**			
**Maternal education**			
Degree plus	1	1	20.5 (3)
Diploma	0.87 (0.58,1.29)	0.88 (0.60,1.28)	
A levels	1.05 (0.72,1.53)	1.06 (0.74,1.52)	
GCSE A-C	1.43 (1.02,1.99)	1.55 (1.14,2.12)	
GCSE D-G	1.78 (1.23,2.58)	2.14 (1.51,3.03)	
None	2.01 (1.44,2.81)	2.42 (1.77,3.30)	
**GROUP 5 - SOCIOECONOMIC AND WIDER FACTORS**			
**Social class**			
Managerial & professional	1	1	26.0 (1)
Intermediate	1.17 (0.88,1.55)	1.14 (0.86,1.51)	
Small employers & own account	1.44 (0.91,2.28)	1.52 (0.99,2.33)	
Lower supervisory & technical	2.01 (1.42,2.86)	1.92 (1.37,2.68)	
Semi-routine & routine	2.41 (1.86,3.12)	2.16 (1.68,2.78)	
Never worked & long-term unemployed	3.34 (2.41,4.63)	2.95 (2.14,4.07)	
**Annual income**			
£33,000+	1	1	20.6 (2)
£22,000-£33,000	1.33 (0.97,1.81)	2.65 (2.01,3.50)	
£11,000-£22,000	1.88 (1.42,2.50)	1.75 (1.32,2.31)	
£0-£11,000	2.98 (2.26,3.92)	1.29 (0.95,1.75)	

For peer review only

**ROC Analysis**	**AUROC = 0.78** (95% CI 0.77 - 0.79) n=11,146	**AUROC = 0.78** (95% CI 0.77 - 0.79) n=11,879

For peer review only

TRIPOD Checklist: Prediction Model Development

Section/Topic	Item	Checklist Item	Page
**Title and abstract**			
Title	1	Identify the study as developing and/or validating a multivariable prediction model, the target population, and the outcome to be predicted.	1
Abstract	2	Provide a summary of objectives, study design, setting, participants, sample size, predictors, outcome, statistical analysis, results, and conclusions.	2
**Introduction**			
Background and objectives	3a	Explain the medical context (including whether diagnostic or prognostic) and rationale for developing or validating the multivariable prediction model, including references to existing models.	3
	3b	Specify the objectives, including whether the study describes the development or validation of the model or both.	3
**Methods**			
Source of data	4a	Describe the study design or source of data (e.g., randomized trial, cohort, or registry data), separately for the development and validation data sets, if applicable.	3
	4b	Specify the key study dates, including start of accrual; end of accrual; and, if applicable, end of follow-up.	3
Participants	5a	Specify key elements of the study setting (e.g., primary care, secondary care, general population) including number and location of centres.	3-4
	5b	Describe eligibility criteria for participants.	4
	5c	Give details of treatments received, if relevant.	
Outcome	6a	Clearly define the outcome that is predicted by the prediction model, including how and when assessed.	4
	6b	Report any actions to blind assessment of the outcome to be predicted.	
Predictors	7a	Clearly define all predictors used in developing or validating the multivariable prediction model, including how and when they were measured.	4-5
	7b	Report any actions to blind assessment of predictors for the outcome and other predictors.	temporal ordering in cohort
Sample size	8	Explain how the study size was arrived at.	5
Missing data	9	Describe how missing data were handled (e.g., complete-case analysis, single imputation, multiple imputation) with details of any imputation method.	6
Statistical analysis methods	10a	Describe how predictors were handled in the analyses.	5-6
	10b	Specify type of model, all model-building procedures (including any predictor selection), and method for internal validation.	6
	10d	Specify all measures used to assess model performance and, if relevant, to compare multiple models.	6
Risk groups	11	Provide details on how risk groups were created, if done.	
**Results**			
Participants	13a	Describe the flow of participants through the study, including the number of participants with and without the outcome and, if applicable, a summary of the follow-up time. A diagram may be helpful.	6
	13b	Describe the characteristics of the participants (basic demographics, clinical features, available predictors), including the number of participants with missing data for predictors and outcome.	6-7
Model development	14a	Specify the number of participants and outcome events in each analysis.	10
	14b	If done, report the unadjusted association between each candidate predictor and outcome.	8-10
Model specification	15a	Present the full prediction model to allow predictions for individuals (i.e., all regression coefficients, and model intercept or baseline survival at a given time point).	
	15b	Explain how to the use the prediction model.	
Model performance	16	Report performance measures (with CIs) for the prediction model.	10
**Discussion**			
Limitations	18	Discuss any limitations of the study (such as nonrepresentative sample, few events per predictor, missing data).	11-12
Interpretation	19b	Give an overall interpretation of the results, considering objectives, limitations, and results from similar studies, and other relevant evidence.	11
Implications	20	Discuss the potential clinical use of the model and implications for future research.	11-13
**Other information**			
Supplementary information	21	Provide information about the availability of supplementary resources, such as study protocol, Web calculator, and data sets.	
Funding	22	Give the source of funding and the role of the funders for the present study.	

We recommend using the TRIPOD Checklist in conjunction with the TRIPOD Explanation and Elaboration document.

For peer review only - http://bmjopen.bmj.com/site/about/guidelines.xhtml

BMJ Open

Development of a Predictive Risk Model for School Readiness at age 3 years using the UK Millennium Cohort Study

Journal:	*BMJ Open*
Manuscript ID	bmjopen-2018-024851.R2
Article Type:	Research
Date Submitted by the Author:	29-Mar-2019
Complete List of Authors:	Camacho, Christine; University of Liverpool Department of Public Health and Policy,<br>Straatmann, Viviane; University of Liverpool Department of Public Health and Policy; Karolinska Institute, Aging Research Centre<br>Day, Jennie; University of Liverpool Department of Public Health and Policy<br>Taylor-Robinson, David; University of Liverpool, Department of Public Health and Policy
**Primary Subject Heading**:	Public health
Secondary Subject Heading:	Public health, Epidemiology, Health policy, Research methods
Keywords:	PUBLIC HEALTH, EPIDEMIOLOGY, SOCIAL MEDICINE

SCHOLARONE™
Manuscripts

Development of a Predictive Risk Model for School Readiness at age 3 years using the UK Millennium Cohort Study

Miss CL Camacho*[1], Dr VS Straatmann[1,2], Dr JC Day[1], Prof DC Taylor-Robinson[1]

Affiliations: [1]*Public Health and Policy, University of Liverpool, Liverpool, UK;* [2]*Aging Research Centre, Karolinska Institute, Stockholm, Sweden*

Address correspondence to: Christine Camacho, Department of Public Health and Policy, University of Liverpool, Liverpool, United Kingdom, L69 3GB, [c.camacho@nhs.net], +44 07973395585

For peer review only

For peer review only - http://bmjopen.bmj.com/site/about/guidelines.xhtml

ABSTRACT

Objectives

The aim of this study was to develop a predictive risk model (PRM) for school readiness measured at age 3 years using perinatal and early infancy data.

Design and Participants

This paper describes the development of a predictive risk model. Predictors were identified from the UK Millennium Cohort Study (MCS) wave 1 data, collected when participants were 9 months old. The outcome was school readiness at age 3 years, measured by the Bracken School Readiness Assessment. Stepwise selection and dominance analysis were used to specify 2 models. The models were compared by the area under the receiver operating characteristic curve (AUROC) and integrated discrimination improvement (IDI).

Results

Data were available for 9,487 complete cases. At age 3, 11.7% (95% CI 11.0-12.3%) of children were not school ready. The variables identified were: parents' Socio-Economic Classification, child's ethnicity, maternal education, income band, sex, household number of children, mother's age, low birth weight, mother's mental health, infant developmental milestones, breastfeeding, parents' employment, housing type. A parsimonious model included the first six listed variables (model 2). The AUROC for model 1 was 0.80 (95% CI 0.78-0.81) and 0.78 (95% CI 0.77-0.79) for model 2. Model 1 resulted in a small improvement in discrimination (IDI=1.3%, $p<0.001$).

Conclusions

Perinatal and infant risk factors predicted school readiness at age 3 with good discrimination. Social determinants were strong predictors of school readiness. This study demonstrates that school readiness can be predicted by six attributes collected around the time of birth.

For peer review only - http://bmjopen.bmj.com/site/about/guidelines.xhtml

Strengths and limitations of this study

- Use of a large, representative, and contemporary cohort study to demonstrate the feasibility of predicting school readiness from data collected in infancy.
- Multiple imputation and bootstrapping were used to evaluate the impact of missing data and internal validity, respectively.
- The main outcome measure, the Bracken School Readiness Assessment, was developed in the US, and is not routinely used in the UK.
- This model was not externally validated, which would have given an indication of generalisability.

INTRODUCTION

Early childhood is a critical time for lifelong physical, social, emotional and cognitive development. A wide range of factors are associated with early cognitive development (ECD)[1]. Interventions in the first three years of life can improve the trajectory of ECD[2] and deliver the greatest return on investment[3], yet it is unclear how best to identify children at most risk of delayed ECD, to enable appropriate targeting of interventions.

Cognitive development measures in children are good indicators of later educational achievement, predict health and social care needs in adults[4,5], and are associated with long term health outcomes[6]. There has been a growing policy interest in school readiness as a measure of ECD[7], and school readiness is a key public health indicator in children in the UK. Good school readiness lays a platform for future learning, employment and health[8,9].

School readiness is currently a major focus in England for policy makers, educators and the public health community [10] and national metrics are collected to capture changes over time. In 2017, 29% of children in England were deemed not school ready at the end of their reception year (aged 4-5 years)[11]. The percentage of children school ready was nearly 20% higher in the most affluent decile (80% school ready) compared to the most deprived decile (62% school ready) when areas were classified into deciles according to the Index for Multiple Deprivation [12]. In UK policy there has been a focus on demographic factors e.g. maternal age, in targeting early interventions for children[13]. This study will explore the importance of different variables in predicting school readiness.

Previous research has identified a wide range of variables associated with early cognitive development. Predictive risk models (PRMs) are well-established in many clinical disciplines and have more recently been applied to child development. Using PRMs in this context could facilitate targeted early intervention as part of a proportionate universalism approach, which requires universal action with the scale and intensity of interventions proportionate to the level of need[6]. Most models thus far have shown fair or poor discrimination and there have been very few studies in the UK [14–18]. The aim of this study was to develop, for the first time, a PRM for school readiness measured at age 3 years using perinatal and early infancy data from the UK Millennium Cohort Study (MCS).

For peer review only - http://bmjopen.bmj.com/site/about/guidelines.xhtml

METHODS

Overview

Data from the MCS were used to explore the relationship between the outcome, school readiness, and 29 predictor variables using logistic regression analysis. Following univariable analysis to test for unadjusted associations, automated stepwise regression analyses were used to select variables for inclusion in the PRM. Dominance analysis was used to rank and weight included predictors, and integrated discrimination improvement (IDI) was calculated to assess the difference in performance between models. A receiver operator characteristic (ROC) curve was used to evaluate how well the model discriminated school readiness. The area under an ROC curve (AUROC) gives a measure of how well the regression model predicts school readiness at age 3. Traditionally accepted AUROC cut off points are: 0.9-1 = excellent, 0.8-<0.9 = good, 0.7-<0.8 = fair, 0.6-<0.7 = poor, 0.5-<0.6 = fail[19]. Multiple imputation was used to assess the impact of missing data in the sample.

Data Source

The PRM was developed and validated using MCS data. The MCS is a nationally representative birth cohort study which recruited 18,550 children born from September 2000 to January 2002, followed up in ongoing data collection waves. The sampling frame was government child benefit records, which had almost universal coverage at the time of sampling. The sample was clustered at the level of electoral ward and stratified to allow over representation of children living in deprived areas and areas with high concentrations of ethnic minorities[20]. Further information about the MCS sample is available in the cohort profile[21]. Data were collected from the main responder (usually mothers) by trained interviewers in participants' homes using a combination of interviews and self-completed questions. All singleton children in the first (aged 9 months) and second (aged 3 years) waves of the MCS with completed data for the outcome and predictors were eligible for inclusion (n=9,487).

Outcome

School readiness was measured using the Bracken School Readiness Assessment (BSRA) which consists of 6 subtests relating to colours, letters, numbers/counting, sizes, comparisons and shapes[20]. The assessment was carried out by interviewers during the second data collection wave when children were aged approximately 3 years old. The BSRA and its predecessors have demonstrated good reliability[22] and validity against other measures and teacher assessments[23].

The BSRA raw scores were summed and adjusted for age to provide a standardised composite score[20]. Scores were grouped according to cut-offs recommended by Bracken which reflected a 'normative classification' whereby children were categorised as very delayed, delayed, average, advanced or very advanced [24]. We used the same cut off score as Bracken (mean standardised composite score <85, 1 standard deviation below mean) but collapsed the categories of delayed or very delayed into a single category equivalent to not being school

For peer review only - http://bmjopen.bmj.com/site/about/guidelines.xhtml

ready. We have dichotomised the outcome 'school readiness' in line with UK policy, and to allow the testing of a PRM using ROC analysis which requires a binary outcome [25].

Predictors

29 predictor variables were used, which were collected at age 9 months in the first wave of MCS data collection during which data relevant to pregnancy, birth and the perinatal period was captured retrospectively. These were identified from previous research to predict cognitive development and were included in the MCS[1,2,4,6,26–33]. The selected predictor variables were grouped according to the Dahlgren and Whitehead theoretical model[34] of social determinants of health as depicted in Figure 1. This model was chosen to provide a framework for categorising predictors to allow analysis of the determinants of early cognitive development.

<<Figure 1 here>>

Group 1 – Demographic and Individual factors

Demographic characteristics included child sex, maternal ethnicity, child weight, pre-term birth, mother's age, home language, maternal mental health and child development categorised as shown in Box 1.

Box 1 – Coding of Group 1 demographic and individual factors

Categorisation of Demographic and Individual factors
Child sex – 'female' and 'male'
Maternal ethnicity – 'white', 'mixed', 'Indian', 'Pakistani and Bangladeshi', 'Black' and 'other'
Child weight at birth – low (<2.5kg) or normal/high (≥2.5kg)
Preterm birth – gestation period less than 37 weeks
Mother's age in years at birth of first child – grouped into 4 categories (14-19, 20-29, 30-39, 40+ years)
Home language – 'English only', 'English and another language', 'another language only'
Mental health (1) – Sad or low for >2 weeks since baby, coded as 'yes' or 'no'
Mental health (2) – Diagnosis of depression or serious anxiety, coded as 'yes' or 'no'
Mental health (3) – 9-item modified version of the Rutter Malaise Inventory[39], coded as 'low' or (0-3) 'high' (4-9) scores[27].
Child development – 8 items from Denver Developmental Screening Test and 5 items from MacArthur Communicative Development Inventory, scored on a continuous scale from 13 (above average) to 36 (below average)

Group 2 – Lifestyle Factors

Self-reported maternal smoking was coded as 'never smoked', 'smoked before pregnancy' and 'smoked during pregnancy'. Maternal alcohol consumption during pregnancy were categorised as 'never or very infrequent', 'occasional', 'regularly' and 'most or everyday'. Breastfeeding duration was grouped as 'never', 'one week or less', '1 – 6 weeks', '6 weeks – 6 months' and 'over 6 months'.

Group 3 – Social and Community Networks

The number of children in household was coded as '1', '2-3' or '4+', and being the eldest or only child was recoded as 'yes' or 'no'. The number of parents or carers was either '1' or '2'.

For peer review only - http://bmjopen.bmj.com/site/about/guidelines.xhtml

Mothers were asked how much time they had spent time in care before the age of 17, this was recoded as 'yes' or 'no' to indicate if they had ever been in care.

Group 4 – Living and Working Conditions

Maternal education was categorised into six groups 'degree plus (higher degree and first degree qualifications)', 'diploma (in higher education)', 'A-levels', 'GCSE grades A–C', 'GCSE grades D–G' and 'none of these qualifications'. Parent's employment status was classified as either 'both', 'one' or 'neither' parents in work[1]. Housing tenure was coded as 'owner occupied', 'private rented', 'social housing' and 'other'. The response to the question, "How common is pollution, grime or other environmental problems?" was recoded as 'common', 'not common' and 'not at all'. Presentation for first antenatal visit was recoded as late if after 12 weeks. Maternal attachment was measured using a 6-item Condon Maternal Attachment Questionnaire[35] grouped as 'low (10-21), 'average' (22-23) and 'high (24-27) .

Group 5 – Socioeconomic and Wider Factors

The National Statistics Socio-Economic Classification (NS-SEC) was used to code job details for main respondents (the majority of which were mothers) as: 'managerial & professional', 'intermediate', 'small employers & own account', 'lower supervisory & technical', 'semi-routine & routine', 'never worked & long-term unemployed'. Net household income was reported by identification of the correct band on a show card and grouped into 4 quartile bands[26]: '£0-£11,000', '£11,000-£22,000', '£22,000-£33,000' and '£33,000+'. Poverty was defined as an equivalised household income 60% below the median before housing costs according to the Organisation for Economic Co-operation and Development Household Equivalence Scale. Families reported receipt of any means-tested benefits, including Jobseekers Allowance, Income Support, Working Families Tax Credit or Disabled Persons Tax Credit. Indices of Multiple Deprivation (IMD) from 2004 which had been retrospectively linked to wave 1 data were used to give small area level deprivation measures [20]. IMD scores were divided into quintiles, with 1 the most deprived quintile, and 5 the least deprived.

Statistical analyses

Analyses were conducted using Stata v14.2 (StataCorp LP, 2017). Survey weights were applied to take account of clustering, stratification and oversampling in the survey design, and attrition between survey waves, using the svyset command (pweight=BOVWT2) and svy prefix for regression modelling[36]. The number of events per variable (EPV) exceeds 35, the predictors were checked for collinearity, a large number of predictors were used and all were significantly associated with the outcome suggesting a robust logistic regression model with sufficient sample size [37,38].

Descriptive analysis of each predictor and school readiness was carried out to ascertain the prevalence of each predictor in the sample. Univariable logistic regression analyses calculating odds ratios (ORs) and 95% confidence intervals (95% CI) were carried out to assess the unadjusted association of each variable with the outcome.

---

[1] Being on leave from work is classed as being in employment

For peer review only - http://bmjopen.bmj.com/site/about/guidelines.xhtml

A multivariable logistic regression model including all 29 variables was reduced using automated forward and backwards stepwise selection (using a cut off p-value of 0.1). Dominance analysis (repeated regression analyses on subsets of variables) was used to produce a ranking and weighting for each predictor in model 1[39]. These rankings were used to specify a more parsimonious model (model 2) containing the top 6 predictors, selected to maximise parsimony and performance. The integrated discrimination improvement (IDI) using the complete case sample from model 1 was calculated to assess difference in performance between models as the percentage change in individuals being correctly assigned by the model[40].

The area under the ROC curve (AUROC) and its 95% CI was used to measure discriminatory power of the models. Classification, including sensitivity and specificity, was assessed at the maximised probability cut off point where the sensitivity and specificity curves intersected. Calibration of the model was assessed using the Pearson Chi-squared test[41]. Bootstrapping was used for internal validation of the final model, without repeating selection of predictors in each bootstrap sample. Model performance was assessed using 1000 bootstrap samples, model optimism was averaged across all iterations to obtain an optimism estimate. An optimism-corrected AUROC, which takes account of overfitting, was calculated by subtracting the optimism estimate from the uncorrected AUROC[42].

A complete case approach was used for the primary analysis. As a sensitivity analysis, multiple imputation by chained equation was performed to impute missing data using the 'mi impute chained' command in Stata. Three predictor variables from the first sweep (maternal education, child's sex, mother's age at birth of first child) and the outcome variable were to shape the imputation process (imputed sample, n=11,897). Twenty imputed datasets were generated, and Rubin's rules were used to calculate results across the imputed datasets[43].

Robustness tests were carried out in which the final model was tested with an alternative outcome measure for early cognitive development (the British Ability Scales, also tested at age 3 in the MCS); different coding of outcome and predictor variables (e.g. maternal age as a continuous variable); and with the addition of another predictor variable (child care type at age 9 months). See supplementary file 1 for further details.

Ethics and Patient and public involvement

Ethical approval for each wave of the MCS was granted by NHS Multicentre Research Ethics Committees[44]. No further ethical approval was required for this secondary analysis of MCS data. There was no direct patient or public involvement in this analysis. However, the MCS has an ongoing programme of participant and public engagement.

RESULTS

There were 15,381 singleton children surveyed in MCS2, of which 13,650 had an outcome recorded for school readiness. Of these children 70% (n=9,487) had complete data for the outcomes and all the predictor variables. There were no significant differences in the

For peer review only - http://bmjopen.bmj.com/site/about/guidelines.xhtml

characteristics of the imputed sample and the complete case sample (p value >0.05 for all chi-squared tests) (Table 1); results are reported for complete cases (see Supplementary file 2 for imputed sample results).

Table 1 - Description of perinatal, sociodemographic and economic characteristics by school ready of sample and imputed sample

	Complete Cases (n=9,487)		Imputed Data (n=11,897)	
**Is Child School Ready?**	**Yes (%)**	**No (%)**	**Yes (%)**	**No (%)**
All	88.3	11.7	85.5	14.5
**GROUP 1 - DEMOGRAPHIC & INDIVIDUAL FACTORS**				
**Gender**				
Female	91.6	8.4	89.4	10.6
Male	85.1	14.9	82.6	17.4
**Ethnicity**				
White	90.4	9.6	88.6	11.4
Mixed	91.1	8.9	84.7	15.3
Indian	79.3	20.7	78.1	21.9
Pakistani and Bangladeshi	55.7	44.3	56.3	43.7
Black or Black British	79.8	20.2	68	32
Other ethnic group	73.6	26.4	74.3	25.7
**Mother's age at birth of first child**				
14-19	78	22	76.4	23.6
20-29	87.9	12.1	86.1	13.9
30-39	95	5	94.4	5.6
40+	76.9	23.1	76	24
**Birth weight (<2500grams)**				
normal/high	88.8	11.2	86.1	13.9
low birthweight	80.2	19.8	77.7	22.3
**Maternal Mental Health (Diagnosed depression/anxiety)**				
No	89	11	86	14
Yes	86	14	84.4	15.6
**Child developmental milestones**				
Child development score (mean, 95%CI)	19.3 (19.2,19.3)	19.9 (19.7,20.1)	19.1 (19.0,19.1)	19.6 (19.4,19.7)
**GROUP 2 - LIFESTYLE FACTORS**				
**Duration of breastfeeding**				
6 months or more	92.5	7.5	90.5	9.5
6 weeks - 6 months	89.8	10.2	87.8	12.2
1 - 6 weeks	88.8	11.2	85.9	14.1
one week or less	88.8	11.2	86.4	13.6
Never	82.6	17.4	80	20
**GROUP 3 - SOCIAL & COMMUNITY NETWORKS**				
**Number of children in family**				
One child	92	8	89.1	10.9
Two or three children	87.7	12.3	85	15
Four or more children	71.7	28.3	70.2	29.8

For peer review only - http://bmjopen.bmj.com/site/about/guidelines.xhtml

**GROUP 4 - LIVING & WORKING CONDITIONS**				
**Maternal education**				
Degree plus	95.6	4.4	95.1	4.9
Diploma	94.6	5.4	93.9	6.1
A levels	92.7	7.3	92	8
GCSE A-C	88.5	11.5	87.4	12.6
GCSE D-G	81	19	79.1	20.9
None	71.3	28.7	69.2	30.8
**Workforce status**				
Both parents in work	92.6	7.4	91.6	8.4
One parent in work	85.8	14.2	83.4	16.6
Neither parent in work	68.5	31.5	70.1	29.9
**Housing tenure**				
Owner occupied	91.9	8.1	90.7	9.3
Private rented	83.8	16.2	80.5	19.5
Social housing	75.8	24.2	74.8	25.2
Other	83.4	16.6	81	19
**GROUP 5 - SOCIOECONOMIC AND WIDER FACTORS**				
**Social class**				
managerial & professional	95.5	4.5	94.6	5.4
intermediate	93.1	6.9	92.1	7.9
small employers & own account	91.3	8.7	89.1	10.9
lower supervisory & technical	87.2	12.8	84	16
semi-routine & routine	81.9	18.1	80	20
never worked & long-term unemployed	60.2	39.8	62.1	37.9
**Annual income**				
£33,000+	95.7	4.3	94.9	5.1
£22,000-£33,000	92.5	7.5	91.7	8.3
£11,000-£22,000	85	15	83.9	16.1
£0-£11,000	73.8	26.2	74.1	25.9

11.7% (95%CI 11.0-12.3%) of children aged 3 years were classified as not being school ready, but this varied significantly by the parents' ethnicity, maternal education and social class (Table 1). All 29 predictor variables were significantly associated with school readiness in univariable logistic regression analysis (p<0.1), so none were excluded at this stage.

The stepwise method reduced the final multivariable logistic regression model to 13 predictors: child's sex and ethnicity, mother's age at birth of first child, birthweight, maternal mental health, child development milestones, duration of breastfeeding, number of children in family, maternal education, parents' workforce status, housing tenure, social class and annual family income. In the adjusted analysis, Pakistani and Bangladeshi children were 4 times more likely to not be school ready than white children (OR 4.19 95% CI 3.14-5.58). The full results are shown in Table 2. There was no evidence of collinearity.

Table 2 - Unadjusted and adjusted associations and dominance analysis for the predictor variables in model 1 (13 predictors)

For peer review only - http://bmjopen.bmj.com/site/about/guidelines.xhtml

Predictors	Unadjusted OR (95% CI)	Adjusted OR (95% CI)	Weighting (rank)
**GROUP 1 - DEMOGRAPHIC & INDIVIDUAL FACTORS**			
**Gender**			
Female	1	1	9.5 (5)
Male	1.76 (1.54,2.01)	2.03 (1.72,2.39)	
**Ethnicity**			
White	1	1	14.7 (2)
Mixed	1.4 (0.96,2.04)	1.42 (0.78,2.58)	
Indian	1.85 (1.23,2.77)	2.58 (1.65,4.03)	
Pakistani and Bangladeshi	5.94 (4.82,7.32)	4.27 (3.20,5.69)	
Black or Black British	4.06 (2.90,5.69)	2.1 (1.13,3.88)	
Other ethnic group	2.33 (1.38,3.93)	2.92 (1.55,5.48)	
**Mother's age at birth of first child**			
30-39	1	1	2.9 (11)
40+	2.83 (2.29,3.49)	1.05 (0.68,1.63)	
20-29	5.57 (4.20,7.37)	1.28 (0.98,1.66)	
14-19	6.02 (4.84,7.48)	1.32 (0.95,1.83)	
**Birth weight (<2500grams)**			
Normal/high	1	1	1.4 (12)
Low birthweight	1.7 (1.34,2.16)	1.26 (0.92,1.72)	
**Maternal Mental Health (Diagnosed depression/anxiety)**			
No	1	1	0.4 (13)
Yes	1.33 (1.16,1.53)	1.28 (1.07,1.53)	
**Child developmental milestones**			
Developmental score	1.07 (1.05,1.10)	1.1 (1.07,1.14)	3.9 (11)
**GROUP 2 - LIFESTYLE FACTORS**			
**Duration of breastfeeding**			
6 months or more	1	1	3.9 (10)
6 weeks - 6 months	1.25 (1.02,1.53)	1.05 (0.81,1.36)	
One week or less	1.67 (1.34,2.09)	1.19 (0.89,1.59)	
1 - 6 weeks	1.68 (1.36,2.07)	1.25 (0.96,1.65)	
Never	2.74 (2.29,3.27)	1.49 (1.19,1.87)	
**GROUP 3 - SOCIAL & COMMUNITY NETWORKS**			
**Number of children in family**			
One child	1	1	7.8 (6)
Two or three children	1.44 (1.27,1.63)	1.38 (1.15,1.66)	
Four or more children	3.71 (3.04,4.54)	2.67 (1.94,3.68)	
**GROUP 4 - LIVING & WORKING CONDITIONS**			
**Maternal education**			
Degree plus	1	1	13.6 (3)
Diploma	1.3 (0.93,1.81)	0.81 (0.53,1.24)	
A levels	1.66 (1.22,2.25)	1.02 (0.68,1.55)	
GCSE A-C	3.02 (2.34,3.90)	1.3 (0.89,1.88)	
GCSE D-G	5.55 (4.21,7.30)	1.54 (1.02,2.34)	
None	9.62 (7.61,12.16)	1.68 (1.15,2.43)	
**Workforce status**			

For peer review only - http://bmjopen.bmj.com/site/about/guidelines.xhtml

Both parents in work	1	1	
One parent in work	1.79 (1.49,2.14)	0.82 (0.67,1.00)	6.9 (7)
Neither parent in work	5.39 (4.36,6.67)	1.21 (0.87,1.68)	
**Housing tenure**			
Owner occupied	1	1	
Private rented	2.68 (2.16,3.33)	1.21 (0.87,1.67)	
Social housing	3.89 (3.34,4.53)	1.45 (1.16,1.81)	5.7 (8)
Other	2.65 (2.10,3.35)	0.9 (0.62,1.30)	
**GROUP 5 - SOCIOECONOMIC AND WIDER FACTORS**			
**Social class**			
Managerial & professional	1	1	
Intermediate	1.5 (1.19,1.89)	1.06 (0.77,1.45)	
Small employers & own account	2.11 (1.44,3.08)	1.41 (0.87,2.28)	
Lower supervisory & technical	3.72 (2.76,5.00)	1.65 (1.09,2.50)	17.4 (1)
Semi-routine & routine	4.99 (4.13,6.01)	1.97 (1.46,2.66)	
Never worked & long-term unemployed	12.07 (9.48,15.37)	2.49 (1.69,3.66)	
**Annual income**			
£33,000+	1	1	
£22,000-£33,000	1.71 (1.31,2.25)	1.31 (0.96,1.79)	
£11,000-£22,000	3.97 (3.12,5.07)	1.64 (1.22,2.22)	12.0 (4)
£0-£11,000	7.7 (6.10,9.72)	2.26 (1.60,3.19)	

Dominance analysis showed that social class was the most important predictor (weighting=17.6), followed by ethnic group (weighting=14.7) and maternal education (weighting=13.8) (Table 2). Analysis of the predictor weightings suggests that social factors (average weighting 11.3, SD 4.9) are stronger predictors of school readiness than demographic and lifestyle factors (average weighting 5.5, SD 4.9). IDI was used to test the relative performance of models with all (1-13) variables, with variables added in according to their rank from the dominance analysis (Supplementary File 3). These analyses informed the specification of model 2, which was comprised of the top 6 predictors: social class, child's ethnic group, maternal education, income band, sex and number of children (see Supplementary File 4 for Model 2 results).

The AUROC was 0.80 (95% CI 0.78-0.81) for model 1 (n=9,487), which indicates a "good" level of discrimination[19]. The AUROC for model 2 (n=11,146) was 0.78 (95% CI 0.77-0.79). Internal validation using bootstrap optimism correction suggests that the model would have good discriminatory power in an independent sample (adjusted AUROC model 1 = 0.79, model 2=0.76). The Pearson Chi-squared tests were both non-significant indicating adequate calibration (model 1, p=0.07, model 2, p=0.13)[45]. IDI showed there was a small but significant difference in performance, with model 1 resulting in a 1.3% (p=<0.001) improvement in discrimination (Figure 2).

<<Figure 2 here>>

Sensitivity and specificity were plotted against probability cut-offs to select the optimal cut off point to assess the PRM's classification (model 1, cut-off=0.12; model 2, cut-off=0.14) (Figure

For peer review only - http://bmjopen.bmj.com/site/about/guidelines.xhtml

For peer review only

3**Error! Reference source not found.**). For model 1, at this cut-off point sensitivity was 72% (95% CI 69.0%-74.3%) and specificity was 74% (95% CI 73.5%-75.3%). Sensitivity of model 2 was similar - 72% (95% CI 69.9%-74.5%). Specificity was lower - 71% (95% CI 69.6%-71.4%), so this model would generate more false positive results than the model 1, but performance was still in the acceptable range. At a probability cut-off of 12%, 31% of the screened population tested would be identified as being 'at risk' of poor school readiness using model 1.

<<Figure 3 here>>

A sensitivity analysis using an alternative outcome measure (British Ability Scales, BAS), showed that the BSRA measure led to improved discrimination (AUROC = 0.79 (95% CI 0.78-0.81) for BAS; AUROC = 0.80 (95% CI 0.78-0.81) for BSRA, p=0.002). See supplementary file 1 for further details.

DISCUSSION

Findings

This study developed a PRM for school readiness at age 3 years using perinatal and early childhood data from the MCS. Model 1 with 13 variables had good discrimination (AUROC=0.80) and classification (sensitivity=72%, specificity= 74% at a maximised cut off). Dominance analysis found the most important variables in predicting school readiness related to socioeconomic conditions (social class, maternal education, family income) and ethnicity. A parsimonious model performed similarly well (AUROC=0.78), suggesting it is possible to predict school readiness at age 3 fairly well using just six variables from the perinatal period and early infancy.

Comparison with previous studies

The value added of this study is that it is the first UK study to show that school readiness can be predicted with good discrimination with a small number of variables collected in infancy. The predictors of school readiness identified here corroborate previous findings. Male sex, maternal education, income, family composition, parental employment, housing and breastfeeding have been identified as significant risk factors of delayed ECD in other studies[4,14,15,17,18,26]. Social factors were the most important predictors, corresponding with current thinking on the social determinants of cognitive development[6,46].

The model reported here has good predictive strength, and compares favourably to similar PRMs, which with one exception[17], achieved only fair or poor discrimination[14,15,18,47]. Chittleborough et al used the ALSPAC UK birth cohort to test the predictive validity of 2 models for ECD[14]. They used a different outcome measure (School entry assessment aged 4-5) and used 6 predictors in their model, which appear to be chosen a priori, rather than by a statistical routine. They found that maternal age alone failed to predict ECD (AUROC~0.5), and a model with 6 predictors achieved only poor discrimination (AUROC=0.67). Camargo-Figuera et al used IQ as a measure of ECD and developed a PRM with 12 predictors using the Brazilian Pelotas birth cohort; their model had good discrimination (AUROC=0.8) and

For peer review only - http://bmjopen.bmj.com/site/about/guidelines.xhtml

calibration, with sensitivity and specificity of 72% and 74% respectively[17]. We believe the use of a representative cohort for model development, stepwise regression to select predictor variables and dominance analysis to specify a simplified model contributed to the good performance of this PRM.

Strengths and Limitations

A strength of this study was the use of a representative and contemporary UK cohort study as the data source. This offered a wide range of predictor variables and a large sample size which minimised the likelihood of overfitting. The cohort design also ensured correct temporal ordering and blinding with respect to the predictors. A theoretical model informed the PRM and statistical selection was used to specify variables. Multiple imputation was used to assess the impact of missing data. Bootstrapping showed good internal validity[48].

There are some limitations of this study to be considered. The main outcome, the BSRA, whilst validated as a measure of school readiness, was developed in the US and is not routinely used in the UK[23]. The BSRA measures a small set of pre-academic skills and as such is a limited measure of child development, which can be defined as including broader behavioural and social skills. However, an analysis of MCS data linked to teacher reports showed that Bracken scores are strongly associated with the broader EYFS measure of school readiness used in English schools[4]. The outcome variable was dichotomised to allow ROC curve analysis. We acknowledge the limitations of dichotomising school readiness ethically, conceptually (e.g. children develop at different rates) and statistically (i.e. loss of information) [49,50]. Longitudinal studies are subject to attrition and non-response which can introduce attrition bias, the use of survey weights partially adjust for this, but it was not possible to use these when calculating the AUROC. Sensitivity analysis using multiple imputation showed the effect of missing data was negligible, similar to other PRMs[14,15]. Most of the predictor variables were based on maternal self-report which may be subject to recall bias, and external validation was not conducted. The predictor variables identified may not be causally associated with school readiness and there are other predictors which may be associated with the outcome which were not included in this model e.g. the home learning environment (which was not assessed at 9 months in the MCS) and childcare in infancy[51].

Policy Implications

The existing literature, and these findings, indicate that a PRM could plausibly be used to identify a group of children at high risk of poor ECD who may benefit from early intervention. If implemented as part of a "proportionate universalism" approach[6], PRMs could mitigate socioeconomic inequalities by providing early years settings with a mechanism for directing their resources to those children at highest risk of poor cognitive development. With new child and maternity datasets now being collected electronically in England, it may be possible to apply a PRM at population level through the use of linked administrative datasets as has been done in Australia[15].

Poor cognitive development is associated with a range of negative health and social outcomes and contributes to inequalities in society[3,5,6], so this is of public health importance.

For peer review only - http://bmjopen.bmj.com/site/about/guidelines.xhtml

Chittleborough et al showed that even a model with poor discrimination has benefits over just using young maternal age to direct resources[14]. Similarly, McKean et al established that their PRM was better than existing clinical tools used to identify higher-risk children for early intervention[47].

The practical implications of using such a PRM as a screening tool should be considered. The model reported here would identify 31% of children screened as being 'at risk' of delayed school readiness. An exemplar English Local Authority with a total population of 230,000, and 3000 children aged under 1 year would identify 900 'at risk' children per year if the PRM was applied to this cohort. This percentage equates with national data; in 2015/16, 31% of children in England were not school ready when tested at age 4-5[11]. However, the overall accuracy of the model is 74%, so over 200 children would be incorrectly classified. PRMs raise ethical issues; labelling very young children as being at risk of poor development could be stigmatising for families, particularly when social factors are the strongest predictors as in this analysis. PRMs would generate false positives (and false negatives), which could cause unnecessary distress and use of resources.

Use of PRMs to identify children at risk of developmental delay should include support and counselling for families, as well as timely access to appropriate interventions. Nelson et al (2016) comment that Early Intervention services would be overwhelmed by the level of demand generated by such PRMs[18]. A criterion for screening programmes is that interventions should be available, it is thus important to further consider the implications of using a PRM to assess ECD in the context of available resources. Investment in early intervention would be required, which would have opportunity costs for services locally. Further research is needed to test the external validity of this PRM for example in another cohort or with linked administrative datasets such as the EYFS data from English schools. Alternative modelling approaches which do not require a dichotomous outcome could also be tested. Findings from such models could offer more nuanced predictions on school readiness.

CONCLUSION

This study has identified a set of predictive risk factors from the perinatal period and early infancy that can predict school readiness at age 3 with a good level of accuracy. Poor cognitive development is socially patterned, evident from a very young age and leads to persistent disadvantage throughout life. It is possible that PRMs could be used to identify high risk children and target appropriate interventions and resources to improve their developmental trajectories, and to reduce social inequalities early in the life course.

STATEMENTS

Funding

This work was supported by the UK Public Health Research Consortium (PHRC). The PHRC is funded by the Department of Health and Social Care Policy Research Programme. The views expressed in this paper are those of the authors and do not necessarily reflect those of the Department of Health and

For peer review only - http://bmjopen.bmj.com/site/about/guidelines.xhtml

Social Care. Information about the wider programme of the PHRC is available from http://phrc.lshtm.ac.uk/. DTR is funded by the MRC on a Clinician Scientist Fellowship (MR/P008577/1).

Competing Interests

We confirm that authors have no conflicts of interest to disclose.

Contributors

CLC, JCD and DTR planned the study. CLC and VSS conducted the analysis under the supervision of DTR. CLC led the drafting of the manuscript. All authors contributed to data interpretation, manuscript drafting and revisions and agreed the submitted version of the manuscript.

Data Sharing

The Millennium Cohort Study dataset is available from the UK Data Service.

ACKNOWLEDGEMENTS

We would like to acknowledge all the families and researchers who are part of the UK Millennium Cohort Study, without whom this research would not have been possible.

FIGURE CAPTIONS

Figure 1 - Rainbow Model showing determinants of school readiness (adapted from Dahlgren and Whitehead, 1991)
Figure 2 - ROC curves for models 1 (13 predictors) and 2 (6 predictors), showing AUROC and IDI
Figure 3 - Maximized probability cut off of sensitivity and specificity of model 1

For peer review only

REFERENCES

1 National Research Council (US) and Institute of Medicine (US) Committee on Integrating the Science of Early Childhood Development. *From Neurons to Neighborhoods: The Science of Early Childhood Development*. Washington (DC): : National Academies Press (US) 2000. http://www.ncbi.nlm.nih.gov/books/NBK225557/

2 Black MM, Walker SP, Fernald LC, *et al.* Early childhood development coming of age: science through the life course. *The Lancet* 2016.

3 Heckman JJ. Skill formation and the economics of investing in disadvantaged children. *Science* 2006;**312**:1900–2. doi:312/5782/1900 [pii]

4 Hobcraft JN, Kiernan KE. Predictive factors from age 3 and infancy for poor child outcomes at age 5 relating to children's development, behaviour and health: evidence from the Millennium Cohort Study. York: : University of York 2010.

5 Caspi A, Houts RM, Belsky DW, *et al.* Childhood forecasting of a small segment of the population with large economic burden. *Nat Hum Behav* 2016;**1**:0005.

6 Marmot M, Allen J, Goldblatt P, *et al.* Fair society, healthy lives: strategic review of health inequalities in England post 2010. Marmot Review Team 2010.

7 Public Health England. Improving school readiness: creating a better start for London. London: 2015.

8 Pia Rebello Britto. School Readiness - A conceptual framework. New York, NY: UNICEF 2012.

9 Marmot M, Friel S, Bell R, *et al.* Closing the gap in a generation: health equity through action on the social determinants of health. *The Lancet* 2008;**372**:1661–9. doi:10.1016/S0140-6736(08)61690-6

10 Abreu L, Roberts N. Children's early years development and school readiness. 2016. https://researchbriefings.parliament.uk/ResearchBriefing/Summary/CDP-2016-0141 (accessed 30 Jan 2019).

11 Public Health England. Public Health Profiles. 2017.https://fingertips.phe.org.uk/profile-group/child-health (accessed 13 Jun 2017).

12 Early years foundation stage profile results: 2017 to 2018. GOV.UK. https://www.gov.uk/government/statistics/early-years-foundation-stage-profile-results-2017-to-2018 (accessed 22 Jan 2019).

13 FPH, FNP. Family Nurse Partnership. Faculty of Public Health 20102015.

14 Chittleborough CR, Lawlor DA, Lynch JW. Young Maternal Age and Poor Child Development: Predictive Validity From a Birth Cohort. *Pediatrics* 2011;**127**:e1436–44. doi:10.1542/peds.2010-3222

For peer review only - http://bmjopen.bmj.com/site/about/guidelines.xhtml

15 Chittleborough CR, Searle AK, Smithers LG, *et al.* How well can poor child development be predicted from early life characteristics?: A whole-of-population data linkage study. *Early Child Res Q* 2016;**35**:19–30.

16 Brownell MD, Ekuma O, Nickel NC, *et al.* A population-based analysis of factors that predict early language and cognitive development. *Early Child Res Q* 2016;**35**:6–18.

17 Camargo-Figuera FA, Barros AJ, Santos IS, *et al.* Early life determinants of low IQ at age 6 in children from the 2004 Pelotas Birth Cohort: a predictive approach. *BMC Pediatr* 2014;**14**:308. doi:10.1186/s12887-014-0308-1

18 Nelson BB, Dudovitz RN, Coker TR, *et al.* Predictors of Poor School Readiness in Children Without Developmental Delay at Age 2. *Pediatrics* 2016;**138**:e20154477. doi:10.1542/peds.2015-4477

19 Pepe MS, Janes H, Longton G, *et al.* Limitations of the Odds Ratio in Gauging the Performance of a Diagnostic, Prognostic, or Screening Marker. *Am J Epidemiol* 2004;**159**:882–90. doi:10.1093/aje/kwh101

20 Kirstine Hansen. Millennium Cohort Study First, Second, Third and Fourth Surveys. A Guide to the Datasets (Seventh Edition). London: : Centre for Longitudinal Studies 2012.

21 Connelly R, Platt L. Cohort Profile: UK Millennium Cohort Study (MCS). *Int J Epidemiol* 2014;**43**:1719–25. doi:10.1093/ije/dyu001

22 Bracken B. Bracken Basic Concept Scale–Revised. San Antonio, TX: : The Psychological Corporation 1998.

23 Panter JE, Bracken BA. Validity of the Bracken School Readiness Assessment for predicting first grade readiness. *Psychol Sch* 2009;**46**:397–409.

24 Connelly R. Millennium Cohort Study Data Note 2013/1: Interpreting Test Scores. London: : Centre for Longitudinal Studies, Institute of Education 2013.

25 Steyerberg E. *Clinical prediction models: a practical approach to development, validation, and updating*. Springer Science & Business Media 2008.

26 Kiernan KE, Mensah FK. Maternal indicators in pregnancy and children's infancy that signal future outcomes for children's development, behaviour and health: evidence from the Millennium Cohort Study. York: : University of York 2010.

27 Kiernan KE, Huerta MC. Economic deprivation, maternal depression, parenting and children's cognitive and emotional development in early childhood. *Br J Sociol* 2008;**59**:783–806.

28 Shenkin SD, Starr JM, Deary IJ. Birth weight and cognitive ability in childhood: a systematic review. *Psychol Bull* 2004;**130**:989.

29 Jefferis BJMH, Power C, Hertzman C. Birth weight, childhood socioeconomic environment, and cognitive development in the 1958 British birth cohort study. *BMJ* 2002;**325**:305. doi:10.1136/bmj.325.7359.305

For peer review only - http://bmjopen.bmj.com/site/about/guidelines.xhtml

30 Kramer MS, Aboud F, Mironova E, *et al.* Breastfeeding and Child Cognitive Development: New Evidence From a Large Randomized Trial. *Arch Gen Psychiatry* 2008;**65**:578–84. doi:10.1001/archpsyc.65.5.578

31 Walker SP, Wachs TD, Grantham-McGregor S, *et al.* Inequality in early childhood: risk and protective factors for early child development. *The Lancet* 2011;**378**:1325–38.

32 Murray GK, Jones PB, Kuh D, *et al.* Infant developmental milestones and subsequent cognitive function. *Ann Neurol* 2007;**62**:128–36. doi:10.1002/ana.21120

33 Kelly Y, Sacker A, Gray R, *et al.* Light drinking in pregnancy, a risk for behavioural problems and cognitive deficits at 3 years of age? *Int J Epidemiol* 2009;**38**:129–40. doi:10.1093/ije/dyn230

34 Dahlgren G, Whitehead M. Policies and strategies to promote social equity in health. *Stockh Inst Future Stud* 1991.

35 Condon JT, Corkindale CJ. The assessment of parent-to-infant attachment: development of a self-report questionnaire instrument. *J Reprod Infant Psychol* 1998;**16**:57–76.

36 Sosthenes C. Ketende, Elizabeth M. Jones. User Guide to Analysing MCS Data Using STATA. Centre for Longitudinal Studies, Institute of Education 2011.

37 Peduzzi P, Concato J, Kemper E, *et al.* A simulation study of the number of events per variable in logistic regression analysis. *J Clin Epidemiol* 1996;**49**:1373–9. doi:10.1016/S0895-4356(96)00236-3

38 Courvoisier DS, Combescure C, Agoritsas T, *et al.* Performance of logistic regression modeling: beyond the number of events per variable, the role of data structure. *J Clin Epidemiol* 2011;**64**:993–1000.

39 Azen R, Traxel N. Using Dominance Analysis to Determine Predictor Importance in Logistic Regression. *J Educ Behav Stat* 2009;**34**:319–47. doi:10.3102/1076998609332754

40 Pencina MJ, D'Agostino RB, Pencina KM, *et al.* Interpreting Incremental Value of Markers Added to Risk Prediction Models. *Am J Epidemiol* 2012;**176**:473–81. doi:10.1093/aje/kws207

41 Windmeijer FAG. The asymptotic distribution of the sum of weighted squared residuals in binary choice models. *Stat Neerlandica* 1990;**44**:69–78.

42 Austin PC, Steyerberg EW. Events per variable (EPV) and the relative performance of different strategies for estimating the out-of-sample validity of logistic regression models. *Stat Methods Med Res* 2017;**26**:796–808.

43 White IR, Royston P, Wood AM. Multiple imputation using chained equations: Issues and guidance for practice. *Stat Med* 2011;**30**:377–99. doi:10.1002/sim.4067

44 Centre for Longitudinal Studies. MCS Ethical Review and Consent. London: Institute of Education 2014.

For peer review only

For peer review only - http://bmjopen.bmj.com/site/about/guidelines.xhtml

45 Hosmer DW, Hjort NL. Goodness-of-fit processes for logistic regression: simulation results. *Stat Med* 2002;**21**:2723–2738.

46 Wilkinson RG, Marmot M. *Social Determinants of Health: The Solid Facts*. World Health Organization 2003.

47 McKean C, Law J, Mensah F, *et al.* Predicting Meaningful Differences in School-Entry Language Skills from Child and Family Factors Measured at 12 months of Age. *Int J Early Child* 2016;**48**:329–51. doi:10.1007/s13158-016-0174-0

48 Steyerberg EW, Harrell FE, Borsboom GJJM, *et al.* Internal validation of predictive models. *J Clin Epidemiol* 2001;**54**:774–81. doi:10.1016/S0895-4356(01)00341-9

49 Senn S. Disappointing dichotomies. *Pharm Stat J Appl Stat Pharm Ind* 2003;**2**:239–240.

50 Altman DG, Royston P. The cost of dichotomising continuous variables. *BMJ* 2006;**332**:1080. doi:10.1136/bmj.332.7549.1080

51 Côté SM, Doyle O, Petitclerc A, *et al.* Child Care in Infancy and Cognitive Performance Until Middle Childhood in the Millennium Cohort Study. *Child Dev* 2013;**84**:1191–208. doi:10.1111/cdev.12049

For peer review only

For peer review only - http://bmjopen.bmj.com/site/about/guidelines.xhtml

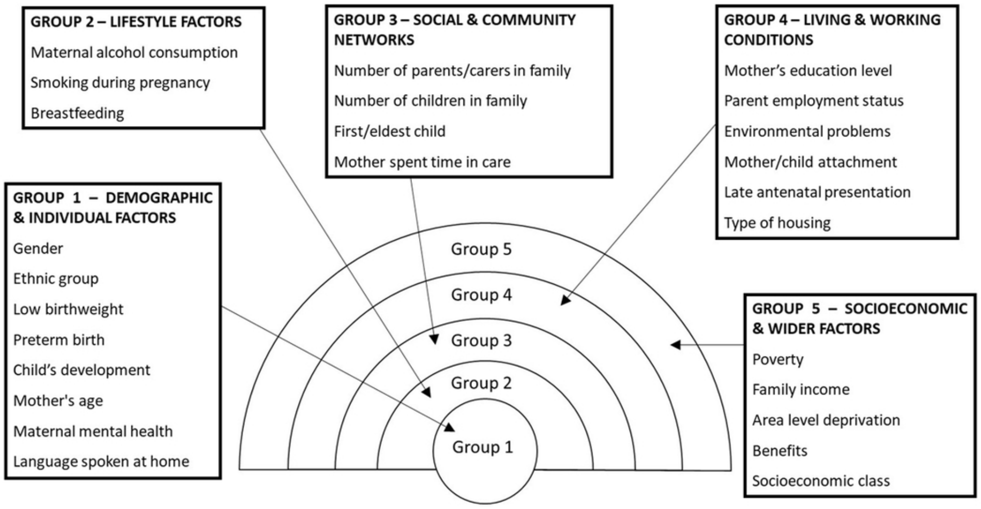

Figure 1 - Rainbow Model showing determinants of school readiness (adapted from Dahlgren and Whitehead, 1991)

90x50mm (300 x 300 DPI)

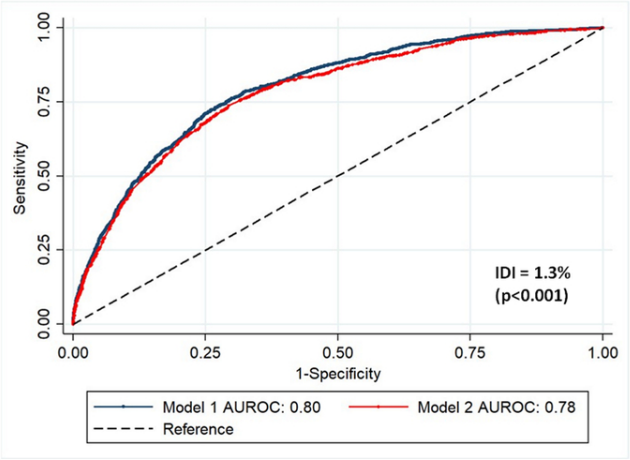

AUROC cut off points are: 0.9-1 = excellent, 0.8-<0.9 = good, 0.7-<0.8 = fair, 0.6-<0.7 = poor, 0.5-<0.6 = fail

Figure 2 - ROC curves for models 1 (13 predictors) and 2 (6 predictors), showing AUROC and IDI

90x50mm (300 x 300 DPI)

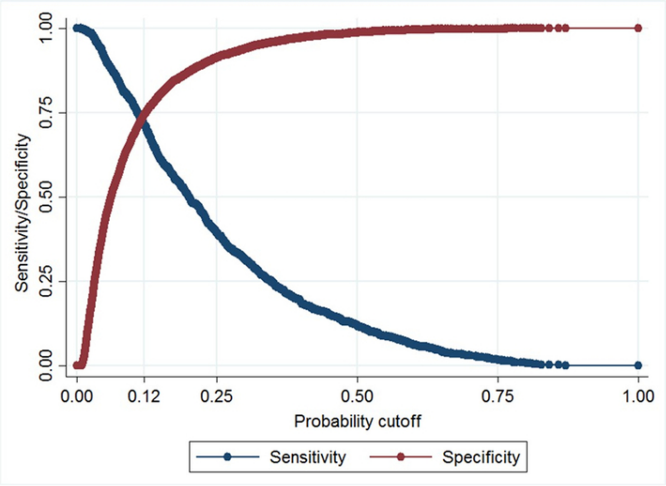

Figure 3 - Maximized probability cut off of sensitivity and specificity of model 1

90x50mm (300 x 300 DPI)

SUPPLEMENTARY FILE 1

Robustness tests were carried out in which the final model was tested with an alternative outcome measure for early cognitive development (British Ability Scales), different coding of variables and the addition of another predictor variable (child care type at age 9 months).

1. Using BAS as an alternative outcome variable
   An alternative measure of early cognitive development contained in the MSC are the British Ability Scales (BAS), measured at age 3. BAS scores were dichotomised to 1 SD below the mean as cut off for 'fail'. There is a moderate positive correlation between BAS and BSRA scores (r=0.5722, p<0.0001). The table below compares performance of the models; there is a small but statistically significant improvement in discrimination using BSRA as an outcome measure compared to BAS.

Outcome variable	N	AUROC (95% CI)
BSRA	9487	0.80 (0.78,0.81)
BAS	9487	0.79 (0.77,0.80)

Ho: area(xb1) = area(xb6); chi2(1) = 9.20, Prob>chi2 = 0.002

2. Robustness tests of the BSRA outcome measure
   The BSRA cut off used in the main analysis was a mean standardised composite score <85, which is 1 standard deviation below the mean. The standardisation sample was from a US population. As the BSRA has not been validated in the UK, we tested the model using dichotomised percentile ranks instead of MSCS as the outcome variable (cut off point 1 SD below mean).

   There was no significant different in model performance (AUROC=0.80 for both models, p=0.43). There is evidence to suggest that within the Millennium Cohort Study percentile scores can be misleading in indicating the difference between the performance of cohort members because they are on an ordinal, rather than interval, scale. An outcome based on MSCS was therefore retained.

3. Coding of predictor variables
   As a sensitivity analysis the coding of 4 predictor variables was altered: maternal age (from categorical to continuous), developmental scores (from categorical to continuous) and ethnicity (from categorical to binary). The impact of this on final model performance is shown below:

Description	n	AUROC	Comparative AUROC (n=9310)
Final model	9487	0.80	0.79 (0.77,0.81)
Developmental score (continuous)	9487	0.80	0.80 (0.78,0.81)
Maternal age (continuous)	9310	0.79	0.79 (0.78,0.81)
Ethnicity (binary)	9487	0.79	0.79 (0.78,0.80)

Ho: area(xb1) = area(xb2) = area(xb3) = area(xb4); chi2(3) = 9.98; Prob>chi2 = 0.02

In summary, there were small but statistically significant differences between the models. The only change which improved model discrimination was using continuous development

For peer review only

scores, so this was incorporated into the final model. There is a U-shaped relationship between school readiness and maternal age, so there was a clear rationale for including this as a categorical predictor.

4. Testing the impact of an additional predictor
There are other measures in the MCS which could have been used as predictors in this analysis. We have done a sensitivity analysis adding childcare type at 9 months to the final model. This reduces the overall discrimination of the model (AUROC = 0.77 vs 0.80), however this could be due to missing data as the child care variable is less complete. There is a statistically significant association with school readiness and child care type in the multivariable model, with children in formal child care settings more likely to be school ready than those being looked after by parents (OR = 1.76, p=0.02)

The Stata Do file for all analyses is available at: https://www.dropbox.com/s/zxsl4cl87imydp0/SchoolreadinessPRM.do?dl=0

For peer review only

SUPPLEMENTARY FILE 2

Table 1 - Adjusted associations for the predictor variables in model 1 (13 predictors) using multiple imputed data (n=11,879)

Predictor	Adjusted OR (95% CI)	Weighting (rank)
**GROUP 1 - DEMOGRAPHIC & INDIVIDUAL FACTORS**		
**Gender**		8.5 (5)
Female	1	
Male	1.86 (1.62,2.14)	
**Ethnicity**		15.7 (3)
White	1	
Mixed	1.04 (0.62,1.75)	
Indian	2.68 (1.85,3.89)	
Pakistani and Bangladeshi	3.85 (2.94,5.04)	
Black or Black British	2.31 (1.43,3.72)	
Other ethnic group	3.95 (2.30,6.77)	
**Mother's age at birth of first child**		1.5 (12)
30-39	1	
40+	1.05 (0.67,1.64)	
20-29	1.22 (0.99,1.51)	
14-19	1.22 (0.93,1.59)	
**Birth weight (<2500grams)**		1.2 (13)
Normal/high	1	
Low birthweight	1.52 (1.18,1.97)	
**Maternal Mental Health (Diagnosed depression/anxiety)**		1.5 (11)
No	1	
Yes	1.15 (0.98,1.34)	
**Child developmental milestones**		
Developmental score	1.10 (1.07,1.13)	2.8 (10)
**GROUP 2 - LIFESTYLE FACTORS**		
**Duration of breastfeeding**		3.6 (9)
6 months or more	1	
6 weeks - 6 months	1.17 (0.92,1.48)	
One week or less	1.15 (0.90,1.48)	
1 - 6 weeks	1.22 (0.96,1.57)	
Never	1.58 (1.29,1.95)	
**GROUP 3 - SOCIAL & COMMUNITY NETWORKS**		
**Number of children in family**		7.1 (6)
One child	1	
Two or three children	1.40 (1.19,1.63)	
Four or more children	2.48 (1.94,3.16)	
**GROUP 4 - LIVING & WORKING CONDITIONS**		
**Maternal education**		16.7 (2)
Degree plus	1	

Diploma	0.88 (0.61,1.26)	
A levels	1.13 (0.80,1.59)	
GCSE A-C	1.34 (1.01,1.78)	
GCSE D-G	1.72 (1.23,2.39)	
None	1.74 (1.28,2.38)	
**Workforce status**		
Both parents in work	1	
One parent in work	0.94 (0.78,1.12)	6.5 (7)
Neither parent in work	1.21 (0.93,1.57)	
**Housing tenure**		
Owner occupied	1	
Private rented	1.18 (0.90,1.54)	5.5 (8)
Social housing	1.43 (1.18,1.72)	
Other	0.96 (0.69,1.35)	
**GROUP 5 - SOCIOECONOMIC AND WIDER FACTORS**		
**Social class**		
Managerial & professional	1	
Intermediate	0.98 (0.75,1.29)	
Small employers & own account	1.32 (0.87,2.00)	17.6 (1)
Lower supervisory & technical	1.50 (1.06,2.13)	
Semi-routine & routine	1.77 (1.38,2.27)	
Never worked & long-term unemployed	2.19 (1.53,3.15)	
**Annual income**		
£33,000+	1	
£22,000-£33,000	1.33 (1.02,1.72)	11.9 (4)
£11,000-£22,000	1.67 (1.30,2.14)	
£0-£11,000	2.14 (1.60,2.87)	
**ROC Analysis**	**AUROC = 0.79 (95% CI 0.78,0.80)**	

For peer review only

SUPPLEMENTARY FILE 3

Integrated discrimination improvement (IDI) analysis was run using Stata function 'idi', which compares the discrimination ability between two logistic regression prediction models. In the first stage of this analysis, the IDI of a PRM with just the strongest predictor variable (social class) was compared to a model with all 13 predictors. Adding the additional 12 predictors lead to a 7.3% increase in IDI. In each subsequent analysis, an additional predictor variable was added according to the ranking of variables from the dominance analysis (Table 1).

Predictor	Weighting	Rank
Social Class	17.38	1
Ethnic group	14.66	2
Maternal education	13.55	3
Income band	12	4
Gender	9.54	5
Number of children	7.84	6
Parent's employment	6.9	7
Housing type	5.65	8
Child development	3.9	9
Breastfeeding	3.9	10
Mother's age at birth of first child	2.87	11
Low birth weight	1.42	12
Mental health	0.38	13

Table 1 - Results of the dominance analysis for model 1

The full results of integrated discrimination improvement (IDI) analysis are shown in Table 2.

Variables included	IDI (%)	p	1-IDI
1	7.3%	<0.00001	92.7%
2	5.3%	<0.00001	94.7%
3	3.8%	<0.00001	96.2%
4	3.5%	<0.00001	96.5%
5	2.3%	<0.00001	97.7%
6	1.3%	<0.00001	98.7%
7	1.0%	<0.00001	99.0%
8	0.9%	<0.00001	99.1%
9	0.6%	0.00001	99.4%
10	0.6%	0.00001	99.4%
11	0.2%	0.01402	99.8%
12	0.0%	0.52356	100.0%

Table 2 - Results of integrated discrimination improvement analysis for 12 models

A 6-predictor model was chosen as this offered the optimal balance between parsimony and discrimination.

SUPPLEMENTARY FILE 4

Table 1 - Adjusted associations for the predictor variables in model 2 (6 predictors) using complete cases (n=11,146) and multiple imputed data (n=11,879). The weightings and rank are from dominance analysis of the complete case sample.

Predictors	Adjusted OR (95% CI) - complete case	Adjusted OR (95% CI) - multiple imputation	Weighting (rank)
**GROUP 1 - DEMOGRAPHIC & INDIVIDUAL FACTORS**			
**Gender**			
Female	1	1	9.9 (5)
Male	1.99 (1.72,2.31)	1.93 (1.68,2.22)	
**Ethnicity**			
White	1	1	13.7 (4)
Mixed	1.2 (0.77,1.88)	1.26 (0.83,1.90)	
Indian	1.64 (1.09,2.47)	1.72 (1.14,2.59)	
Pakistani and Bangladeshi	2.67 (2.10,3.41)	2.71 (2.11,3.47)	
Black or Black British	2.32 (1.52,3.54)	2.69 (1.80,4.02)	
Other ethnic group	1.98 (1.10,3.58)	2.06 (1.27,3.32)	
**GROUP 3 - SOCIAL & COMMUNITY NETWORKS**			
**Number of children in family**			
One child	1	1	9.5 (6)
Two or three children	1.48 (1.27,1.73)	1.45 (1.25,1.69)	
Four or more children	2.89 (2.23,3.75)	2.62 (2.03,3.38)	
**GROUP 4 - LIVING & WORKING CONDITIONS**			
**Maternal education**			
Degree plus	1	1	20.5 (3)
Diploma	0.87 (0.58,1.29)	0.88 (0.60,1.28)	
A levels	1.05 (0.72,1.53)	1.06 (0.74,1.52)	
GCSE A-C	1.43 (1.02,1.99)	1.55 (1.14,2.12)	
GCSE D-G	1.78 (1.23,2.58)	2.14 (1.51,3.03)	
None	2.01 (1.44,2.81)	2.42 (1.77,3.30)	
**GROUP 5 - SOCIOECONOMIC AND WIDER FACTORS**			
**Social class**			
Managerial & professional	1	1	26.0 (1)
Intermediate	1.17 (0.88,1.55)	1.14 (0.86,1.51)	
Small employers & own account	1.44 (0.91,2.28)	1.52 (0.99,2.33)	
Lower supervisory & technical	2.01 (1.42,2.86)	1.92 (1.37,2.68)	
Semi-routine & routine	2.41 (1.86,3.12)	2.16 (1.68,2.78)	
Never worked & long-term unemployed	3.34 (2.41,4.63)	2.95 (2.14,4.07)	
**Annual income**			
£33,000+	1	1	20.6 (2)
£22,000-£33,000	1.33 (0.97,1.81)	2.65 (2.01,3.50)	
£11,000-£22,000	1.88 (1.42,2.50)	1.75 (1.32,2.31)	
£0-£11,000	2.98 (2.26,3.92)	1.29 (0.95,1.75)	

**ROC Analysis**	**AUROC = 0.78** (95% CI 0.77 - 0.79) n=11,146	**AUROC = 0.78** (95% CI 0.77 - 0.79) n=11,879

For peer review only

TRIPOD Checklist: Prediction Model Development

Section/Topic	Item	Checklist Item	Page
**Title and abstract**			
Title	1	Identify the study as developing and/or validating a multivariable prediction model, the target population, and the outcome to be predicted.	1
Abstract	2	Provide a summary of objectives, study design, setting, participants, sample size, predictors, outcome, statistical analysis, results, and conclusions.	2
**Introduction**			
Background and objectives	3a	Explain the medical context (including whether diagnostic or prognostic) and rationale for developing or validating the multivariable prediction model, including references to existing models.	3
	3b	Specify the objectives, including whether the study describes the development or validation of the model or both.	3
**Methods**			
Source of data	4a	Describe the study design or source of data (e.g., randomized trial, cohort, or registry data), separately for the development and validation data sets, if applicable.	3
	4b	Specify the key study dates, including start of accrual; end of accrual; and, if applicable, end of follow-up.	3
Participants	5a	Specify key elements of the study setting (e.g., primary care, secondary care, general population) including number and location of centres.	3-4
	5b	Describe eligibility criteria for participants.	4
	5c	Give details of treatments received, if relevant.	
Outcome	6a	Clearly define the outcome that is predicted by the prediction model, including how and when assessed.	4
	6b	Report any actions to blind assessment of the outcome to be predicted.	
Predictors	7a	Clearly define all predictors used in developing or validating the multivariable prediction model, including how and when they were measured.	4-5
	7b	Report any actions to blind assessment of predictors for the outcome and other predictors.	temporal ordering in cohort
Sample size	8	Explain how the study size was arrived at.	5
Missing data	9	Describe how missing data were handled (e.g., complete-case analysis, single imputation, multiple imputation) with details of any imputation method.	6
Statistical analysis methods	10a	Describe how predictors were handled in the analyses.	5-6
	10b	Specify type of model, all model-building procedures (including any predictor selection), and method for internal validation.	6
	10d	Specify all measures used to assess model performance and, if relevant, to compare multiple models.	6
Risk groups	11	Provide details on how risk groups were created, if done.	
**Results**			
Participants	13a	Describe the flow of participants through the study, including the number of participants with and without the outcome and, if applicable, a summary of the follow-up time. A diagram may be helpful.	6
	13b	Describe the characteristics of the participants (basic demographics, clinical features, available predictors), including the number of participants with missing data for predictors and outcome.	6-7
Model development	14a	Specify the number of participants and outcome events in each analysis.	10
	14b	If done, report the unadjusted association between each candidate predictor and outcome.	8-10
Model specification	15a	Present the full prediction model to allow predictions for individuals (i.e., all regression coefficients, and model intercept or baseline survival at a given time point).	
	15b	Explain how to the use the prediction model.	
Model performance	16	Report performance measures (with CIs) for the prediction model.	10
**Discussion**			
Limitations	18	Discuss any limitations of the study (such as nonrepresentative sample, few events per predictor, missing data).	11-12
Interpretation	19b	Give an overall interpretation of the results, considering objectives, limitations, and results from similar studies, and other relevant evidence.	11
Implications	20	Discuss the potential clinical use of the model and implications for future research.	11-13
**Other information**			
Supplementary information	21	Provide information about the availability of supplementary resources, such as study protocol, Web calculator, and data sets.	
Funding	22	Give the source of funding and the role of the funders for the present study.	

We recommend using the TRIPOD Checklist in conjunction with the TRIPOD Explanation and Elaboration document.

For peer review only - http://bmjopen.bmj.com/site/about/guidelines.xhtml

BMJ Open

Development of a Predictive Risk Model for School Readiness at age 3 years using the UK Millennium Cohort Study

Journal:	*BMJ Open*
Manuscript ID	bmjopen-2018-024851.R3
Article Type:	Research
Date Submitted by the Author:	07-May-2019
Complete List of Authors:	Camacho, Christine; University of Liverpool Department of Public Health and Policy,<br>Straatmann, Viviane; University of Liverpool Department of Public Health and Policy; Karolinska Institute, Aging Research Centre<br>Day, Jennie; University of Liverpool Department of Public Health and Policy<br>Taylor-Robinson, David; University of Liverpool, Department of Public Health and Policy
**Primary Subject Heading**:	Public health
Secondary Subject Heading:	Public health, Epidemiology, Health policy, Research methods
Keywords:	PUBLIC HEALTH, EPIDEMIOLOGY, SOCIAL MEDICINE

SCHOLARONE™
Manuscripts

Development of a Predictive Risk Model for School Readiness at age 3 years using the UK Millennium Cohort Study

Miss CL Camacho*[1], Dr VS Straatmann[1,2], Dr JC Day[1], Prof DC Taylor-Robinson[1]

Affiliations: [1]Public Health and Policy, University of Liverpool, Liverpool, UK; [2]Aging Research Centre, Karolinska Institute, Stockholm, Sweden

Address correspondence to: Christine Camacho, Department of Public Health and Policy, University of Liverpool, Liverpool, United Kingdom, L69 3GB, [c.camacho@nhs.net], +44 07973395585

For peer review only

For peer review only - http://bmjopen.bmj.com/site/about/guidelines.xhtml

ABSTRACT

Objectives

The aim of this study was to develop a predictive risk model (PRM) for school readiness measured at age 3 years using perinatal and early infancy data.

Design and Participants

This paper describes the development of a predictive risk model. Predictors were identified from the UK Millennium Cohort Study (MCS) wave 1 data, collected when participants were 9 months old. The outcome was school readiness at age 3 years, measured by the Bracken School Readiness Assessment. Stepwise selection and dominance analysis were used to specify 2 models. The models were compared by the area under the receiver operating characteristic curve (AUROC) and integrated discrimination improvement (IDI).

Results

Data were available for 9,487 complete cases. At age 3, 11.7% (95% CI 11.0-12.3%) of children were not school ready. The variables identified were: parents' Socio-Economic Classification, child's ethnicity, maternal education, income band, sex, household number of children, mother's age, low birth weight, mother's mental health, infant developmental milestones, breastfeeding, parents' employment, housing type. A parsimonious model included the first six listed variables (model 2). The AUROC for model 1 was 0.80 (95% CI 0.78-0.81) and 0.78 (95% CI 0.77-0.79) for model 2. Model 1 resulted in a small improvement in discrimination (IDI=1.3%, p<0.001).

Conclusions

Perinatal and infant risk factors predicted school readiness at age 3 with good discrimination. Social determinants were strong predictors of school readiness. This study demonstrates that school readiness can be predicted by six attributes collected around the time of birth.

For peer review only

For peer review only - http://bmjopen.bmj.com/site/about/guidelines.xhtml

Strengths and limitations of this study

- Use of a large, representative, and contemporary cohort study to demonstrate the feasibility of predicting school readiness from data collected in infancy.
- Multiple imputation and bootstrapping were used to evaluate the impact of missing data and internal validity, respectively.
- The main outcome measure, the Bracken School Readiness Assessment, was developed in the US, and is not routinely used in the UK.
- This model was not externally validated, which would have given an indication of generalisability.

INTRODUCTION

Early childhood is a critical time for lifelong physical, social, emotional and cognitive development. A wide range of factors are associated with early cognitive development (ECD)[1]. Interventions in the first three years of life can improve the trajectory of ECD[2] and deliver the greatest return on investment[3], yet it is unclear how best to identify children at most risk of delayed ECD, to enable appropriate targeting of interventions.

Cognitive development measures in children are good indicators of later educational achievement, predict health and social care needs in adults[4,5], and are associated with long term health outcomes[6]. There has been a growing policy interest in school readiness as a measure of ECD[7], and school readiness is a key public health indicator in children in the UK. Good school readiness lays a platform for future learning, employment and health[8,9].

School readiness is currently a major focus in England for policy makers, educators and the public health community [10] and national metrics are collected to capture changes over time. In 2017, 29% of children in England were deemed not school ready at the end of their reception year (aged 4-5 years)[11]. The percentage of children school ready was nearly 20% higher in the most affluent decile (80% school ready) compared to the most deprived decile (62% school ready) when areas were classified into deciles according to the Index for Multiple Deprivation [12]. In UK policy there has been a focus on demographic factors e.g. maternal age, in targeting early interventions for children[13]. This study will explore the importance of different variables in predicting school readiness.

Previous research has identified a wide range of variables associated with early cognitive development. Predictive risk models (PRMs) are well-established in many clinical disciplines and have more recently been applied to child development. Using PRMs in this context could facilitate targeted early intervention as part of a proportionate universalism approach, which requires universal action with the scale and intensity of interventions proportionate to the level of need[6]. Most models thus far have shown fair or poor discrimination and there have been very few studies in the UK [14–18]. The aim of this study was to develop, for the first time, a PRM for school readiness measured at age 3 years using perinatal and early infancy data from the UK Millennium Cohort Study (MCS).

For peer review only - http://bmjopen.bmj.com/site/about/guidelines.xhtml

METHODS

Overview

Data from the MCS were used to explore the relationship between the outcome, school readiness, and 29 predictor variables using logistic regression analysis. Following univariable analysis to test for unadjusted associations, automated stepwise regression analyses were used to select variables for inclusion in the PRM. Dominance analysis was used to rank and weight included predictors, and integrated discrimination improvement (IDI) was calculated to assess the difference in performance between models. A receiver operator characteristic (ROC) curve was used to evaluate how well the model discriminated school readiness. The area under an ROC curve (AUROC) gives a measure of how well the regression model predicts school readiness at age 3. Traditionally accepted AUROC cut off points are: 0.9-1 = excellent, 0.8-<0.9 = good, 0.7-<0.8 = fair, 0.6-<0.7 = poor, 0.5-<0.6 = fail[19]. Multiple imputation was used to assess the impact of missing data in the sample.

Data Source

The PRM was developed and validated using MCS data. The MCS is a nationally representative birth cohort study which recruited 18,550 children born from September 2000 to January 2002, followed up in ongoing data collection waves. The sampling frame was government child benefit records, which had almost universal coverage at the time of sampling. The sample was clustered at the level of electoral ward and stratified to allow over representation of children living in deprived areas and areas with high concentrations of ethnic minorities[20]. Further information about the MCS sample is available in the cohort profile[21]. Data were collected from the main responder (usually mothers) by trained interviewers in participants' homes using a combination of interviews and self-completed questions. All singleton children in the first (aged 9 months) and second (aged 3 years) waves of the MCS with completed data for the outcome and predictors were eligible for inclusion (n=9,487).

Outcome

School readiness was measured using the Bracken School Readiness Assessment (BSRA) which consists of 6 subtests relating to colours, letters, numbers/counting, sizes, comparisons and shapes[20]. The assessment was carried out by interviewers during the second data collection wave when children were aged approximately 3 years old. The BSRA and its predecessors have demonstrated good reliability[22] and validity against other measures and teacher assessments[23].

The BSRA raw scores were summed and adjusted for age to provide a standardised composite score[20]. Scores were grouped according to cut-offs recommended by Bracken which reflected a 'normative classification' whereby children were categorised as very delayed, delayed, average, advanced or very advanced [24]. We used the same cut off score as Bracken (mean standardised composite score <85, 1 standard deviation below mean) but collapsed the categories of delayed or very delayed into a single category equivalent to not being school

For peer review only - http://bmjopen.bmj.com/site/about/guidelines.xhtml

ready. We have dichotomised the outcome 'school readiness' in line with UK policy, and to allow the testing of a PRM using ROC analysis which requires a binary outcome [25].

Predictors

29 predictor variables were used, which were collected at age 9 months in the first wave of MCS data collection during which data relevant to pregnancy, birth and the perinatal period was captured retrospectively. These were identified from previous research to predict cognitive development and were included in the MCS[1,2,4,6,26–33]. The selected predictor variables were grouped according to the Dahlgren and Whitehead theoretical model[34] of social determinants of health as depicted in Figure 1. This model was chosen to provide a framework for categorising predictors to allow analysis of the determinants of early cognitive development.

<<Figure 1 here>>

Group 1 – Demographic and Individual factors

Demographic characteristics included child sex, maternal ethnicity, child weight, pre-term birth, mother's age, home language, maternal mental health and child development categorised as shown in Box 1.

Box 1 – Coding of Group 1 demographic and individual factors

Categorisation of Demographic and Individual factors
Child sex – 'female' and 'male'
Maternal ethnicity – 'white', 'mixed', 'Indian', 'Pakistani and Bangladeshi', 'Black' and 'other'
Child weight at birth – low (<2.5kg) or normal/high (≥2.5kg)
Preterm birth – gestation period less than 37 weeks
Mother's age in years at birth of first child – grouped into 4 categories (14-19, 20-29, 30-39, 40+ years)
Home language – 'English only', 'English and another language', 'another language only'
Mental health (1) – Sad or low for >2 weeks since baby, coded as 'yes' or 'no'
Mental health (2) – Diagnosis of depression or serious anxiety, coded as 'yes' or 'no'
Mental health (3) – 9-item modified version of the Rutter Malaise Inventory[39], coded as 'low' or (0-3) 'high' (4-9) scores[27].
Child development – 8 items from Denver Developmental Screening Test and 5 items from MacArthur Communicative Development Inventory, scored on a continuous scale from 13 (above average) to 36 (below average)

Group 2 – Lifestyle Factors

Self-reported maternal smoking was coded as 'never smoked', 'smoked before pregnancy' and 'smoked during pregnancy'. Maternal alcohol consumption during pregnancy were categorised as 'never or very infrequent', 'occasional', 'regularly' and 'most or everyday'. Breastfeeding duration was grouped as 'never', 'one week or less', '1 – 6 weeks', '6 weeks – 6 months' and 'over 6 months'.

Group 3 – Social and Community Networks

The number of children in household was coded as '1', '2-3' or '4+', and being the eldest or only child was recoded as 'yes' or 'no'. The number of parents or carers was either '1' or '2'.

For peer review only - http://bmjopen.bmj.com/site/about/guidelines.xhtml

Mothers were asked how much time they had spent time in care before the age of 17, this was recoded as 'yes' or 'no' to indicate if they had ever been in care.

Group 4 – Living and Working Conditions

Maternal education was categorised into six groups 'degree plus (higher degree and first degree qualifications)', 'diploma (in higher education)', 'A-levels', 'GCSE grades A–C', 'GCSE grades D–G' and 'none of these qualifications'. Parent's employment status was classified as either 'both', 'one' or 'neither' parents in work[1]. Housing tenure was coded as 'owner occupied', 'private rented', 'social housing' and 'other'. The response to the question, "How common is pollution, grime or other environmental problems?" was recoded as 'common', 'not common' and 'not at all'. Presentation for first antenatal visit was recoded as late if after 12 weeks. Maternal attachment was measured using a 6-item Condon Maternal Attachment Questionnaire[35] grouped as 'low (10-21), 'average' (22-23) and 'high (24-27) .

Group 5 – Socioeconomic and Wider Factors

The National Statistics Socio-Economic Classification (NS-SEC) was used to code job details for main respondents (the majority of which were mothers) as: 'managerial & professional', 'intermediate', 'small employers & own account', 'lower supervisory & technical', 'semi-routine & routine', 'never worked & long-term unemployed'. Net household income was reported by identification of the correct band on a show card and grouped into 4 quartile bands[26]: '£0-£11,000', '£11,000-£22,000', '£22,000-£33,000' and '£33,000+'. Poverty was defined as an equivalised household income 60% below the median before housing costs according to the Organisation for Economic Co-operation and Development Household Equivalence Scale. Families reported receipt of any means-tested benefits, including Jobseekers Allowance, Income Support, Working Families Tax Credit or Disabled Persons Tax Credit. Indices of Multiple Deprivation (IMD) from 2004 which had been retrospectively linked to wave 1 data were used to give small area level deprivation measures [20]. IMD scores were divided into quintiles, with 1 the most deprived quintile, and 5 the least deprived.

Statistical analyses

Analyses were conducted using Stata v14.2 (StataCorp LP, 2017). Survey weights were applied to take account of clustering, stratification and oversampling in the survey design, and attrition between survey waves, using the svyset command (pweight=BOVWT2) and svy prefix for regression modelling[36]. The number of events per variable (EPV) exceeds 35, the predictors were checked for collinearity, a large number of predictors were used and all were significantly associated with the outcome suggesting a robust logistic regression model with sufficient sample size [37,38].

Descriptive analysis of each predictor and school readiness was carried out to ascertain the prevalence of each predictor in the sample. Univariable logistic regression analyses calculating odds ratios (ORs) and 95% confidence intervals (95% CI) were carried out to assess the unadjusted association of each variable with the outcome.

[1] Being on leave from work is classed as being in employment

For peer review only - http://bmjopen.bmj.com/site/about/guidelines.xhtml

A multivariable logistic regression model including all 29 variables was reduced using automated forward and backwards stepwise selection (using a cut off p-value of 0.1). Dominance analysis (repeated regression analyses on subsets of variables) was used to produce a ranking and weighting for each predictor in model 1[39]. These rankings were used to specify a more parsimonious model (model 2) containing the top 6 predictors, selected to maximise parsimony and performance. The integrated discrimination improvement (IDI) using the complete case sample from model 1 was calculated to assess difference in performance between models as the percentage change in individuals being correctly assigned by the model[40].

The area under the ROC curve (AUROC) and its 95% CI was used to measure discriminatory power of the models. Classification, including sensitivity and specificity, was assessed at the maximised probability cut off point where the sensitivity and specificity curves intersected. Calibration of the model was assessed using the Pearson Chi-squared test[41]. Bootstrapping was used for internal validation of the final model, without repeating selection of predictors in each bootstrap sample. Model performance was assessed using 1000 bootstrap samples, model optimism was averaged across all iterations to obtain an optimism estimate. An optimism-corrected AUROC, which takes account of overfitting, was calculated by subtracting the optimism estimate from the uncorrected AUROC[42].

A complete case approach was used for the primary analysis. As a sensitivity analysis, multiple imputation by chained equation was performed to impute missing data using the 'mi impute chained' command in Stata. We used predictor variables with relatively little missing data (maternal education, child's sex, mother's age at birth of first child) and the outcome as regular variables in the imputation model. As such individuals with missing data for these 4 items were not included in the final imputed sample (n=11,897). Twenty imputed datasets were generated, and Rubin's rules were used to calculate results across the imputed datasets[43].

Robustness tests were carried out in which the final model was tested with an alternative outcome measure for early cognitive development (the British Ability Scales, also tested at age 3 in the MCS); different coding of outcome and predictor variables (e.g. maternal age as a continuous variable); and with the addition of another predictor variable (child care type at age 9 months). See supplementary file 1 for further details.

Ethics and Patient and public involvement

Ethical approval for each wave of the MCS was granted by NHS Multicentre Research Ethics Committees[44]. No further ethical approval was required for this secondary analysis of MCS data. There was no direct patient or public involvement in this analysis. However, the MCS has an ongoing programme of participant and public engagement.

RESULTS

There were 15,381 singleton children surveyed in MCS2, of which 13,650 had an outcome recorded for school readiness. Of these children 70% (n=9,487) had complete data for the

For peer review only - http://bmjopen.bmj.com/site/about/guidelines.xhtml

outcomes and all the predictor variables. There were no significant differences in the characteristics of the imputed sample and the complete case sample (p value >0.05 for all chi-squared tests) (Table 1); results are reported for complete cases (see Supplementary file 2 for imputed sample results).

Table 1 - Description of perinatal, sociodemographic and economic characteristics by school ready of sample and imputed sample

	Complete Cases (n=9,487)		Imputed Data (n=11,897)	
**Is Child School Ready?**	**Yes (%)**	**No (%)**	**Yes (%)**	**No (%)**
All	88.3	11.7	85.5	14.5
**GROUP 1 - DEMOGRAPHIC & INDIVIDUAL FACTORS**				
**Gender**				
Female	91.6	8.4	89.4	10.6
Male	85.1	14.9	82.6	17.4
**Ethnicity**				
White	90.4	9.6	88.6	11.4
Mixed	91.1	8.9	84.7	15.3
Indian	79.3	20.7	78.1	21.9
Pakistani and Bangladeshi	55.7	44.3	56.3	43.7
Black or Black British	79.8	20.2	68	32
Other ethnic group	73.6	26.4	74.3	25.7
**Mother's age at birth of first child**				
14-19	78	22	76.4	23.6
20-29	87.9	12.1	86.1	13.9
30-39	95	5	94.4	5.6
40+	76.9	23.1	76	24
**Birth weight (<2500grams)**				
normal/high	88.8	11.2	86.1	13.9
low birthweight	80.2	19.8	77.7	22.3
**Maternal Mental Health (Diagnosed depression/anxiety)**				
No	89	11	86	14
Yes	86	14	84.4	15.6
**Child developmental milestones**				
Child development score (mean, 95%CI)	19.3 (19.2,19.3)	19.9 (19.7,20.1)	19.1 (19.0,19.1)	19.6 (19.4,19.7)
**GROUP 2 - LIFESTYLE FACTORS**				
**Duration of breastfeeding**				
6 months or more	92.5	7.5	90.5	9.5
6 weeks - 6 months	89.8	10.2	87.8	12.2
1 - 6 weeks	88.8	11.2	85.9	14.1
one week or less	88.8	11.2	86.4	13.6
Never	82.6	17.4	80	20
**GROUP 3 - SOCIAL & COMMUNITY NETWORKS**				
**Number of children in family**				
One child	92	8	89.1	10.9
Two or three children	87.7	12.3	85	15

For peer review only - http://bmjopen.bmj.com/site/about/guidelines.xhtml

Four or more children	71.7	28.3	70.2	29.8
**GROUP 4 - LIVING & WORKING CONDITIONS**				
**Maternal education**				
Degree plus	95.6	4.4	95.1	4.9
Diploma	94.6	5.4	93.9	6.1
A levels	92.7	7.3	92	8
GCSE A-C	88.5	11.5	87.4	12.6
GCSE D-G	81	19	79.1	20.9
None	71.3	28.7	69.2	30.8
**Workforce status**				
Both parents in work	92.6	7.4	91.6	8.4
One parent in work	85.8	14.2	83.4	16.6
Neither parent in work	68.5	31.5	70.1	29.9
**Housing tenure**				
Owner occupied	91.9	8.1	90.7	9.3
Private rented	83.8	16.2	80.5	19.5
Social housing	75.8	24.2	74.8	25.2
Other	83.4	16.6	81	19
**GROUP 5 - SOCIOECONOMIC AND WIDER FACTORS**				
**Social class**				
managerial & professional	95.5	4.5	94.6	5.4
intermediate	93.1	6.9	92.1	7.9
small employers & own account	91.3	8.7	89.1	10.9
lower supervisory & technical	87.2	12.8	84	16
semi-routine & routine	81.9	18.1	80	20
never worked & long-term unemployed	60.2	39.8	62.1	37.9
**Annual income**				
£33,000+	95.7	4.3	94.9	5.1
£22,000-£33,000	92.5	7.5	91.7	8.3
£11,000-£22,000	85	15	83.9	16.1
£0-£11,000	73.8	26.2	74.1	25.9

11.7% (95%CI 11.0-12.3%) of children aged 3 years were classified as not being school ready, but this varied significantly by the parents' ethnicity, maternal education and social class (Table 1). All 29 predictor variables were significantly associated with school readiness in univariable logistic regression analysis (p<0.1), so none were excluded at this stage.

The stepwise method reduced the final multivariable logistic regression model to 13 predictors: child's sex and ethnicity, mother's age at birth of first child, birthweight, maternal mental health, child development milestones, duration of breastfeeding, number of children in family, maternal education, parents' workforce status, housing tenure, social class and annual family income. In the adjusted analysis, Pakistani and Bangladeshi children were 4 times more likely to not be school ready than white children (OR 4.19 95% CI 3.14-5.58). The full results are shown in Table 2. There was no evidence of collinearity.

For peer review only - http://bmjopen.bmj.com/site/about/guidelines.xhtml

Table 2 - Unadjusted and adjusted associations and dominance analysis for the predictor variables in model 1 (13 predictors)

Predictors	Unadjusted OR (95% CI)	Adjusted OR (95% CI)	Weighting (rank)
**GROUP 1 - DEMOGRAPHIC & INDIVIDUAL FACTORS**			
**Gender**			
Female	1	1	9.5 (5)
Male	1.76 (1.54,2.01)	2.03 (1.72,2.39)	
**Ethnicity**			
White	1	1	14.7 (2)
Mixed	1.4 (0.96,2.04)	1.42 (0.78,2.58)	
Indian	1.85 (1.23,2.77)	2.58 (1.65,4.03)	
Pakistani and Bangladeshi	5.94 (4.82,7.32)	4.27 (3.20,5.69)	
Black or Black British	4.06 (2.90,5.69)	2.1 (1.13,3.88)	
Other ethnic group	2.33 (1.38,3.93)	2.92 (1.55,5.48)	
**Mother's age at birth of first child**			
30-39	1	1	2.9 (11)
40+	2.83 (2.29,3.49)	1.05 (0.68,1.63)	
20-29	5.57 (4.20,7.37)	1.28 (0.98,1.66)	
14-19	6.02 (4.84,7.48)	1.32 (0.95,1.83)	
**Birth weight (<2500grams)**			
Normal/high	1	1	1.4 (12)
Low birthweight	1.7 (1.34,2.16)	1.26 (0.92,1.72)	
**Maternal Mental Health (Diagnosed depression/anxiety)**			
No	1	1	0.4 (13)
Yes	1.33 (1.16,1.53)	1.28 (1.07,1.53)	
**Child developmental milestones**			
Developmental score	1.07 (1.05,1.10)	1.1 (1.07,1.14)	3.9 (11)
**GROUP 2 - LIFESTYLE FACTORS**			
**Duration of breastfeeding**			
6 months or more	1	1	3.9 (10)
6 weeks - 6 months	1.25 (1.02,1.53)	1.05 (0.81,1.36)	
One week or less	1.67 (1.34,2.09)	1.19 (0.89,1.59)	
1 - 6 weeks	1.68 (1.36,2.07)	1.25 (0.96,1.65)	
Never	2.74 (2.29,3.27)	1.49 (1.19,1.87)	
**GROUP 3 - SOCIAL & COMMUNITY NETWORKS**			
**Number of children in family**			
One child	1	1	7.8 (6)
Two or three children	1.44 (1.27,1.63)	1.38 (1.15,1.66)	
Four or more children	3.71 (3.04,4.54)	2.67 (1.94,3.68)	
**GROUP 4 - LIVING & WORKING CONDITIONS**			
**Maternal education**			
Degree plus	1	1	13.6 (3)
Diploma	1.3 (0.93,1.81)	0.81 (0.53,1.24)	
A levels	1.66 (1.22,2.25)	1.02 (0.68,1.55)	
GCSE A-C	3.02 (2.34,3.90)	1.3 (0.89,1.88)	
GCSE D-G	5.55 (4.21,7.30)	1.54 (1.02,2.34)	

For peer review only - http://bmjopen.bmj.com/site/about/guidelines.xhtml

None	9.62 (7.61,12.16)	1.68 (1.15,2.43)	
**Workforce status**			
Both parents in work	1	1	
One parent in work	1.79 (1.49,2.14)	0.82 (0.67,1.00)	6.9 (7)
Neither parent in work	5.39 (4.36,6.67)	1.21 (0.87,1.68)	
**Housing tenure**			
Owner occupied	1	1	
Private rented	2.68 (2.16,3.33)	1.21 (0.87,1.67)	5.7 (8)
Social housing	3.89 (3.34,4.53)	1.45 (1.16,1.81)	
Other	2.65 (2.10,3.35)	0.9 (0.62,1.30)	
**GROUP 5 - SOCIOECONOMIC AND WIDER FACTORS**			
**Social class**			
Managerial & professional	1	1	
Intermediate	1.5 (1.19,1.89)	1.06 (0.77,1.45)	
Small employers & own account	2.11 (1.44,3.08)	1.41 (0.87,2.28)	17.4 (1)
Lower supervisory & technical	3.72 (2.76,5.00)	1.65 (1.09,2.50)	
Semi-routine & routine	4.99 (4.13,6.01)	1.97 (1.46,2.66)	
Never worked & long-term unemployed	12.07 (9.48,15.37)	2.49 (1.69,3.66)	
**Annual income**			
£33,000+	1	1	
£22,000-£33,000	1.71 (1.31,2.25)	1.31 (0.96,1.79)	12.0 (4)
£11,000-£22,000	3.97 (3.12,5.07)	1.64 (1.22,2.22)	
£0-£11,000	7.7 (6.10,9.72)	2.26 (1.60,3.19)	

Dominance analysis showed that social class was the most important predictor (weighting=17.6), followed by ethnic group (weighting=14.7) and maternal education (weighting=13.8) (Table 2). Analysis of the predictor weightings suggests that social factors (average weighting 11.3, SD 4.9) are stronger predictors of school readiness than demographic and lifestyle factors (average weighting 5.5, SD 4.9). IDI was used to test the relative performance of models with all (1-13) variables, with variables added in according to their rank from the dominance analysis (Supplementary File 3). These analyses informed the specification of model 2, which was comprised of the top 6 predictors: social class, child's ethnic group, maternal education, income band, sex and number of children (see Supplementary File 4 for Model 2 results).

The AUROC was 0.80 (95% CI 0.78-0.81) for model 1 (n=9,487), which indicates a "good" level of discrimination[19]. The AUROC for model 2 (n=11,146) was 0.78 (95% CI 0.77-0.79). Internal validation using bootstrap optimism correction suggests that the model would have good discriminatory power in an independent sample (adjusted AUROC model 1 = 0.79, model 2=0.76). The Pearson Chi-squared tests were both non-significant indicating adequate calibration (model 1, p=0.07, model 2, p=0.13)[45]. IDI showed there was a small but significant difference in performance, with model 1 resulting in a 1.3% (p=<0.001) improvement in discrimination (Figure 2).

<<Figure 2 here>>

For peer review only - http://bmjopen.bmj.com/site/about/guidelines.xhtml

Sensitivity and specificity were plotted against probability cut-offs to select the optimal cut off point to assess the PRM's classification (model 1, cut-off=0.12; model 2, cut-off=0.14) (Figure 3**Error! Reference source not found.**). For model 1, at this cut-off point sensitivity was 72% (95% CI 69.0%-74.3%) and specificity was 74% (95% CI 73.5%-75.3%). Sensitivity of model 2 was similar - 72% (95% CI 69.9%-74.5%). Specificity was lower - 71% (95% CI 69.6%-71.4%), so this model would generate more false positive results than the model 1, but performance was still in the acceptable range. At a probability cut-off of 12%, 31% of the screened population tested would be identified as being 'at risk' of poor school readiness using model 1.

<<Figure 3 here>>

A sensitivity analysis using an alternative outcome measure (British Ability Scales, BAS), showed that the BSRA measure led to improved discrimination (AUROC = 0.79 (95% CI 0.78-0.81) for BAS; AUROC = 0.80 (95% CI 0.78-0.81) for BSRA, p=0.002). See supplementary file 1 for further details.

DISCUSSION

Findings

This study developed a PRM for school readiness at age 3 years using perinatal and early childhood data from the MCS. Model 1 with 13 variables had good discrimination (AUROC=0.80) and classification (sensitivity=72%, specificity= 74% at a maximised cut off). Dominance analysis found the most important variables in predicting school readiness related to socioeconomic conditions (social class, maternal education, family income) and ethnicity. A parsimonious model performed similarly well (AUROC=0.78), suggesting it is possible to predict school readiness at age 3 fairly well using just six variables from the perinatal period and early infancy.

Comparison with previous studies

The value added of this study is that it is the first UK study to show that school readiness can be predicted with good discrimination with a small number of variables collected in infancy. The predictors of school readiness identified here corroborate previous findings. Male sex, maternal education, income, family composition, parental employment, housing and breastfeeding have been identified as significant risk factors of delayed ECD in other studies[4,14,15,17,18,26]. Social factors were the most important predictors, corresponding with current thinking on the social determinants of cognitive development[6,46].

The model reported here has good predictive strength, and compares favourably to similar PRMs, which with one exception[17], achieved only fair or poor discrimination[14,15,18,47]. Chittleborough et al used the ALSPAC UK birth cohort to test the predictive validity of 2 models for ECD[14]. They used a different outcome measure (School entry assessment aged 4-5) and used 6 predictors in their model, which appear to be chosen a priori, rather than by a statistical routine. They found that maternal age alone failed to predict ECD (AUROC~0.5), and a model with 6 predictors achieved only poor discrimination (AUROC=0.67). Camargo-

For peer review only - http://bmjopen.bmj.com/site/about/guidelines.xhtml

Figuera et al used IQ as a measure of ECD and developed a PRM with 12 predictors using the Brazilian Pelotas birth cohort; their model had good discrimination (AUROC=0.8) and calibration, with sensitivity and specificity of 72% and 74% respectively[17]. We believe the use of a representative cohort for model development, stepwise regression to select predictor variables and dominance analysis to specify a simplified model contributed to the good performance of this PRM.

Strengths and Limitations

A strength of this study was the use of a representative and contemporary UK cohort study as the data source. This offered a wide range of predictor variables and a large sample size which minimised the likelihood of overfitting. The cohort design also ensured correct temporal ordering and blinding with respect to the predictors. A theoretical model informed the PRM and statistical selection was used to specify variables. Multiple imputation was used to assess the impact of missing data. Bootstrapping showed good internal validity[48].

There are some limitations of this study to be considered. The main outcome, the BSRA, whilst validated as a measure of school readiness, was developed in the US and is not routinely used in the UK[23]. The BSRA measures a small set of pre-academic skills and as such is a limited measure of child development, which can be defined as including broader behavioural and social skills. However, an analysis of MCS data linked to teacher reports showed that Bracken scores are strongly associated with the broader EYFS measure of school readiness used in English schools[4]. The outcome variable was dichotomised to allow ROC curve analysis. We acknowledge the limitations of dichotomising school readiness ethically, conceptually (e.g. children develop at different rates) and statistically (i.e. loss of information) [49,50]. Longitudinal studies are subject to attrition and non-response which can introduce attrition bias, the use of survey weights partially adjust for this, but it was not possible to use these when calculating the AUROC. Sensitivity analysis using multiple imputation showed the effect of missing data was negligible, similar to other PRMs[14,15]. Most of the predictor variables were based on maternal self-report which may be subject to recall bias, and external validation was not conducted. The predictor variables identified may not be causally associated with school readiness and there are other predictors which may be associated with the outcome which were not included in this model e.g. the home learning environment (which was not assessed at 9 months in the MCS) and childcare in infancy[51].

Policy Implications

The existing literature, and these findings, indicate that a PRM could plausibly be used to identify a group of children at high risk of poor ECD who may benefit from early intervention. If implemented as part of a "proportionate universalism" approach[6], PRMs could mitigate socioeconomic inequalities by providing early years settings with a mechanism for directing their resources to those children at highest risk of poor cognitive development. With new child and maternity datasets now being collected electronically in England, it may be possible to apply a PRM at population level through the use of linked administrative datasets as has been done in Australia[15].

For peer review only - http://bmjopen.bmj.com/site/about/guidelines.xhtml

Poor cognitive development is associated with a range of negative health and social outcomes and contributes to inequalities in society[3,5,6], so this is of public health importance. Chittleborough et al showed that even a model with poor discrimination has benefits over just using young maternal age to direct resources[14]. Similarly, McKean et al established that their PRM was better than existing clinical tools used to identify higher-risk children for early intervention[47].

The practical implications of using such a PRM as a screening tool should be considered. The model reported here would identify 31% of children screened as being 'at risk' of delayed school readiness. An exemplar English Local Authority with a total population of 230,000, and 3000 children aged under 1 year would identify 900 'at risk' children per year if the PRM was applied to this cohort. This percentage equates with national data; in 2015/16, 31% of children in England were not school ready when tested at age 4-5[11]. However, the overall accuracy of the model is 74%, so over 200 children would be incorrectly classified. PRMs raise ethical issues; labelling very young children as being at risk of poor development could be stigmatising for families, particularly when social factors are the strongest predictors as in this analysis. PRMs would generate false positives (and false negatives), which could cause unnecessary distress and use of resources.

Use of PRMs to identify children at risk of developmental delay should include support and counselling for families, as well as timely access to appropriate interventions. Nelson et al (2016) comment that Early Intervention services would be overwhelmed by the level of demand generated by such PRMs[18]. A criterion for screening programmes is that interventions should be available, it is thus important to further consider the implications of using a PRM to assess ECD in the context of available resources. Investment in early intervention would be required, which would have opportunity costs for services locally. Further research is needed to test the external validity of this PRM for example in another cohort or with linked administrative datasets such as the EYFS data from English schools. Alternative modelling approaches which do not require a dichotomous outcome could also be tested. Findings from such models could offer more nuanced predictions on school readiness.

CONCLUSION

This study has identified a set of predictive risk factors from the perinatal period and early infancy that can predict school readiness at age 3 with a good level of accuracy. Poor cognitive development is socially patterned, evident from a very young age and leads to persistent disadvantage throughout life. It is possible that PRMs could be used to identify high risk children and target appropriate interventions and resources to improve their developmental trajectories, and to reduce social inequalities early in the life course.

STATEMENTS

Funding

For peer review only - http://bmjopen.bmj.com/site/about/guidelines.xhtml

This work was supported by the UK Public Health Research Consortium (PHRC). The PHRC is funded by the Department of Health and Social Care Policy Research Programme. The views expressed in this paper are those of the authors and do not necessarily reflect those of the Department of Health and Social Care. Information about the wider programme of the PHRC is available from http://phrc.lshtm.ac.uk/. DTR is funded by the MRC on a Clinician Scientist Fellowship (MR/P008577/1).

Competing Interests

We confirm that authors have no conflicts of interest to disclose.

Contributors

CLC, JCD and DTR planned the study. CLC and VSS conducted the analysis under the supervision of DTR. CLC led the drafting of the manuscript. All authors contributed to data interpretation, manuscript drafting and revisions and agreed the submitted version of the manuscript.

Data Sharing

The Millennium Cohort Study dataset is available from the UK Data Service.

Millennium Cohort Study: First Survey, 2001-2003: http://doi.org/10.5255/UKDA-SN-4683-4

Millennium Cohort Study: Second Survey, 2003-2005: http://doi.org/10.5255/UKDA-SN-5350-4

The Data Collection is available to Registered or Authorised Users.

ACKNOWLEDGEMENTS

We would like to acknowledge all the families and researchers who are part of the UK Millennium Cohort Study, without whom this research would not have been possible.

FIGURE CAPTIONS

Figure 1 - Rainbow Model showing determinants of school readiness (adapted from Dahlgren and Whitehead, 1991)

Figure 2 - ROC curves for models 1 (13 predictors) and 2 (6 predictors), showing AUROC and IDI

Figure 3 - Maximized probability cut off of sensitivity and specificity of model 1

For peer review only - http://bmjopen.bmj.com/site/about/guidelines.xhtml

For peer review only

For peer review only

For peer review only - http://bmjopen.bmj.com/site/about/guidelines.xhtml

REFERENCES

1 National Research Council (US) and Institute of Medicine (US) Committee on Integrating the Science of Early Childhood Development. *From Neurons to Neighborhoods: The Science of Early Childhood Development*. Washington (DC): : National Academies Press (US) 2000. http://www.ncbi.nlm.nih.gov/books/NBK225557/

2 Black MM, Walker SP, Fernald LC, *et al.* Early childhood development coming of age: science through the life course. *The Lancet* 2016.

3 Heckman JJ. Skill formation and the economics of investing in disadvantaged children. *Science* 2006;**312**:1900–2. doi:312/5782/1900 [pii]

4 Hobcraft JN, Kiernan KE. Predictive factors from age 3 and infancy for poor child outcomes at age 5 relating to children's development, behaviour and health: evidence from the Millennium Cohort Study. York: : University of York 2010.

5 Caspi A, Houts RM, Belsky DW, *et al.* Childhood forecasting of a small segment of the population with large economic burden. *Nat Hum Behav* 2016;**1**:0005.

6 Marmot M, Allen J, Goldblatt P, *et al.* Fair society, healthy lives: strategic review of health inequalities in England post 2010. Marmot Review Team 2010.

7 Public Health England. Improving school readiness: creating a better start for London. London: 2015.

8 Pia Rebello Britto. School Readiness - A conceptual framework. New York, NY: UNICEF 2012.

9 Marmot M, Friel S, Bell R, *et al.* Closing the gap in a generation: health equity through action on the social determinants of health. *The Lancet* 2008;**372**:1661–9. doi:10.1016/S0140-6736(08)61690-6

10 Abreu L, Roberts N. Children's early years development and school readiness. 2016. https://researchbriefings.parliament.uk/ResearchBriefing/Summary/CDP-2016-0141 (accessed 30 Jan 2019).

11 Public Health England. Public Health Profiles. 2017.https://fingertips.phe.org.uk/profile-group/child-health (accessed 13 Jun 2017).

12 Early years foundation stage profile results: 2017 to 2018. GOV.UK. https://www.gov.uk/government/statistics/early-years-foundation-stage-profile-results-2017-to-2018 (accessed 22 Jan 2019).

13 FPH, FNP. Family Nurse Partnership. Faculty of Public Health 20102015.

14 Chittleborough CR, Lawlor DA, Lynch JW. Young Maternal Age and Poor Child Development: Predictive Validity From a Birth Cohort. *Pediatrics* 2011;**127**:e1436–44. doi:10.1542/peds.2010-3222

For peer review only - http://bmjopen.bmj.com/site/about/guidelines.xhtml

15 Chittleborough CR, Searle AK, Smithers LG, *et al.* How well can poor child development be predicted from early life characteristics?: A whole-of-population data linkage study. *Early Child Res Q* 2016;**35**:19–30.

16 Brownell MD, Ekuma O, Nickel NC, *et al.* A population-based analysis of factors that predict early language and cognitive development. *Early Child Res Q* 2016;**35**:6–18.

17 Camargo-Figuera FA, Barros AJ, Santos IS, *et al.* Early life determinants of low IQ at age 6 in children from the 2004 Pelotas Birth Cohort: a predictive approach. *BMC Pediatr* 2014;**14**:308. doi:10.1186/s12887-014-0308-1

18 Nelson BB, Dudovitz RN, Coker TR, *et al.* Predictors of Poor School Readiness in Children Without Developmental Delay at Age 2. *Pediatrics* 2016;**138**:e20154477. doi:10.1542/peds.2015-4477

19 Pepe MS, Janes H, Longton G, *et al.* Limitations of the Odds Ratio in Gauging the Performance of a Diagnostic, Prognostic, or Screening Marker. *Am J Epidemiol* 2004;**159**:882–90. doi:10.1093/aje/kwh101

20 Kirstine Hansen. Millennium Cohort Study First, Second, Third and Fourth Surveys. A Guide to the Datasets (Seventh Edition). London: : Centre for Longitudinal Studies 2012.

21 Connelly R, Platt L. Cohort Profile: UK Millennium Cohort Study (MCS). *Int J Epidemiol* 2014;**43**:1719–25. doi:10.1093/ije/dyu001

22 Bracken B. Bracken Basic Concept Scale–Revised. San Antonio, TX: : The Psychological Corporation 1998.

23 Panter JE, Bracken BA. Validity of the Bracken School Readiness Assessment for predicting first grade readiness. *Psychol Sch* 2009;**46**:397–409.

24 Connelly R. Millennium Cohort Study Data Note 2013/1: Interpreting Test Scores. London: : Centre for Longitudinal Studies, Institute of Education 2013.

25 Steyerberg E. *Clinical prediction models: a practical approach to development, validation, and updating*. Springer Science & Business Media 2008.

26 Kiernan KE, Mensah FK. Maternal indicators in pregnancy and children's infancy that signal future outcomes for children's development, behaviour and health: evidence from the Millennium Cohort Study. York: : University of York 2010.

27 Kiernan KE, Huerta MC. Economic deprivation, maternal depression, parenting and children's cognitive and emotional development in early childhood. *Br J Sociol* 2008;**59**:783–806.

28 Shenkin SD, Starr JM, Deary IJ. Birth weight and cognitive ability in childhood: a systematic review. *Psychol Bull* 2004;**130**:989.

29 Jefferis BJMH, Power C, Hertzman C. Birth weight, childhood socioeconomic environment, and cognitive development in the 1958 British birth cohort study. *BMJ* 2002;**325**:305. doi:10.1136/bmj.325.7359.305

For peer review only - http://bmjopen.bmj.com/site/about/guidelines.xhtml

30 Kramer MS, Aboud F, Mironova E, *et al.* Breastfeeding and Child Cognitive Development: New Evidence From a Large Randomized Trial. *Arch Gen Psychiatry* 2008;**65**:578–84. doi:10.1001/archpsyc.65.5.578

31 Walker SP, Wachs TD, Grantham-McGregor S, *et al.* Inequality in early childhood: risk and protective factors for early child development. *The Lancet* 2011;**378**:1325–38.

32 Murray GK, Jones PB, Kuh D, *et al.* Infant developmental milestones and subsequent cognitive function. *Ann Neurol* 2007;**62**:128–36. doi:10.1002/ana.21120

33 Kelly Y, Sacker A, Gray R, *et al.* Light drinking in pregnancy, a risk for behavioural problems and cognitive deficits at 3 years of age? *Int J Epidemiol* 2009;**38**:129–40. doi:10.1093/ije/dyn230

34 Dahlgren G, Whitehead M. Policies and strategies to promote social equity in health. *Stockh Inst Future Stud* 1991.

35 Condon JT, Corkindale CJ. The assessment of parent-to-infant attachment: development of a self-report questionnaire instrument. *J Reprod Infant Psychol* 1998;**16**:57–76.

36 Sosthenes C. Ketende, Elizabeth M. Jones. User Guide to Analysing MCS Data Using STATA. Centre for Longitudinal Studies, Institute of Education 2011.

37 Peduzzi P, Concato J, Kemper E, *et al.* A simulation study of the number of events per variable in logistic regression analysis. *J Clin Epidemiol* 1996;**49**:1373–9. doi:10.1016/S0895-4356(96)00236-3

38 Courvoisier DS, Combescure C, Agoritsas T, *et al.* Performance of logistic regression modeling: beyond the number of events per variable, the role of data structure. *J Clin Epidemiol* 2011;**64**:993–1000.

39 Azen R, Traxel N. Using Dominance Analysis to Determine Predictor Importance in Logistic Regression. *J Educ Behav Stat* 2009;**34**:319–47. doi:10.3102/1076998609332754

40 Pencina MJ, D'Agostino RB, Pencina KM, *et al.* Interpreting Incremental Value of Markers Added to Risk Prediction Models. *Am J Epidemiol* 2012;**176**:473–81. doi:10.1093/aje/kws207

41 Windmeijer FAG. The asymptotic distribution of the sum of weighted squared residuals in binary choice models. *Stat Neerlandica* 1990;**44**:69–78.

42 Austin PC, Steyerberg EW. Events per variable (EPV) and the relative performance of different strategies for estimating the out-of-sample validity of logistic regression models. *Stat Methods Med Res* 2017;**26**:796–808.

43 White IR, Royston P, Wood AM. Multiple imputation using chained equations: Issues and guidance for practice. *Stat Med* 2011;**30**:377–99. doi:10.1002/sim.4067

44 Centre for Longitudinal Studies. MCS Ethical Review and Consent. London: Institute of Education 2014.

For peer review only - http://bmjopen.bmj.com/site/about/guidelines.xhtml

For peer review only

45 Hosmer DW, Hjort NL. Goodness-of-fit processes for logistic regression: simulation results. *Stat Med* 2002;**21**:2723–2738.

46 Wilkinson RG, Marmot M. *Social Determinants of Health: The Solid Facts*. World Health Organization 2003.

47 McKean C, Law J, Mensah F, *et al.* Predicting Meaningful Differences in School-Entry Language Skills from Child and Family Factors Measured at 12 months of Age. *Int J Early Child* 2016;**48**:329–51. doi:10.1007/s13158-016-0174-0

48 Steyerberg EW, Harrell FE, Borsboom GJJM, *et al.* Internal validation of predictive models. *J Clin Epidemiol* 2001;**54**:774–81. doi:10.1016/S0895-4356(01)00341-9

49 Senn S. Disappointing dichotomies. *Pharm Stat J Appl Stat Pharm Ind* 2003;**2**:239–240.

50 Altman DG, Royston P. The cost of dichotomising continuous variables. *BMJ* 2006;**332**:1080. doi:10.1136/bmj.332.7549.1080

51 Côté SM, Doyle O, Petitclerc A, *et al.* Child Care in Infancy and Cognitive Performance Until Middle Childhood in the Millennium Cohort Study. *Child Dev* 2013;**84**:1191–208. doi:10.1111/cdev.12049

For peer review only

For peer review only - http://bmjopen.bmj.com/site/about/guidelines.xhtml

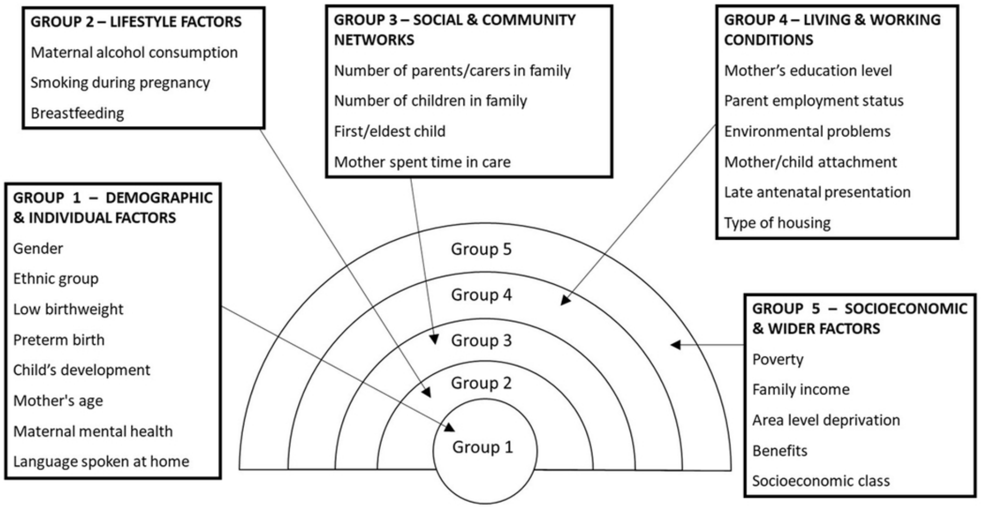

Figure 1 - Rainbow Model showing determinants of school readiness (adapted from Dahlgren and Whitehead, 1991)

90x50mm (300 x 300 DPI)

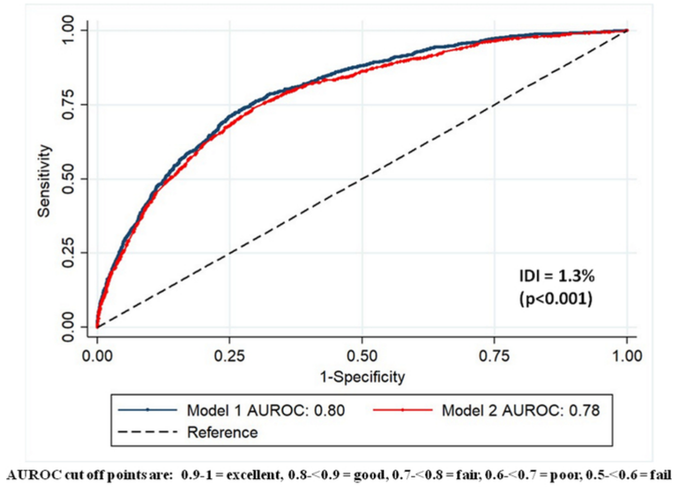

Figure 2 - ROC curves for models 1 (13 predictors) and 2 (6 predictors), showing AUROC and IDI

90x50mm (300 x 300 DPI)

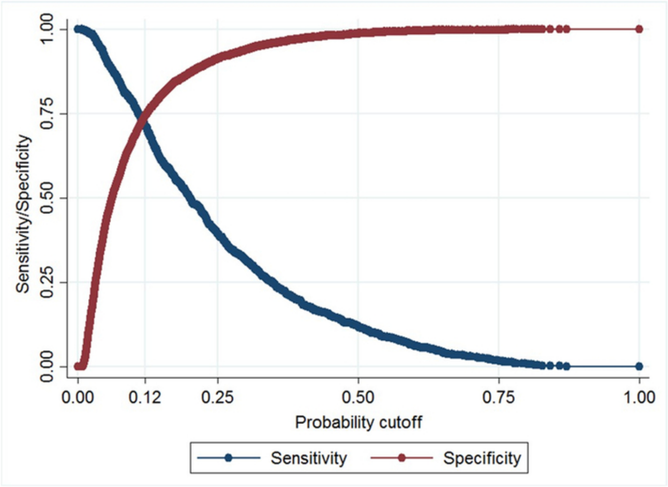

Figure 3 - Maximized probability cut off of sensitivity and specificity of model 1

90x50mm (300 x 300 DPI)

SUPPLEMENTARY FILE 1

Robustness tests were carried out in which the final model was tested with an alternative outcome measure for early cognitive development (British Ability Scales), different coding of variables and the addition of another predictor variable (child care type at age 9 months).

1. Using BAS as an alternative outcome variable
   An alternative measure of early cognitive development contained in the MSC are the British Ability Scales (BAS), measured at age 3. BAS scores were dichotomised to 1 SD below the mean as cut off for 'fail'. There is a moderate positive correlation between BAS and BSRA scores (r=0.5722, p<0.0001). The table below compares performance of the models; there is a small but statistically significant improvement in discrimination using BSRA as an outcome measure compared to BAS.

Outcome variable	N	AUROC (95% CI)
BSRA	9487	0.80 (0.78,0.81)
BAS	9487	0.79 (0.77,0.80)

Ho: area(xb1) = area(xb6); chi2(1) = 9.20, Prob>chi2 = 0.002

2. Robustness tests of the BSRA outcome measure
   The BSRA cut off used in the main analysis was a mean standardised composite score <85, which is 1 standard deviation below the mean. The standardisation sample was from a US population. As the BSRA has not been validated in the UK, we tested the model using dichotomised percentile ranks instead of MSCS as the outcome variable (cut off point 1 SD below mean).

   There was no significant different in model performance (AUROC=0.80 for both models, p=0.43). There is evidence to suggest that within the Millennium Cohort Study percentile scores can be misleading in indicating the difference between the performance of cohort members because they are on an ordinal, rather than interval, scale. An outcome based on MSCS was therefore retained.

3. Coding of predictor variables
   As a sensitivity analysis the coding of 4 predictor variables was altered: maternal age (from categorical to continuous), developmental scores (from categorical to continuous) and ethnicity (from categorical to binary). The impact of this on final model performance is shown below:

Description	n	AUROC	Comparative AUROC (n=9310)
Final model	9487	0.80	0.79 (0.77,0.81)
Developmental score (continuous)	9487	0.80	0.80 (0.78,0.81)
Maternal age (continuous)	9310	0.79	0.79 (0.78,0.81)
Ethnicity (binary)	9487	0.79	0.79 (0.78,0.80)

Ho: area(xb1) = area(xb2) = area(xb3) = area(xb4); chi2(3) = 9.98; Prob>chi2 = 0.02

In summary, there were small but statistically significant differences between the models. The only change which improved model discrimination was using continuous development

For peer review only

scores, so this was incorporated into the final model. There is a U-shaped relationship between school readiness and maternal age, so there was a clear rationale for including this as a categorical predictor.

4. Testing the impact of an additional predictor
There are other measures in the MCS which could have been used as predictors in this analysis. We have done a sensitivity analysis adding childcare type at 9 months to the final model. This reduces the overall discrimination of the model (AUROC = 0.77 vs 0.80), however this could be due to missing data as the child care variable is less complete. There is a statistically significant association with school readiness and child care type in the multivariable model, with children in formal child care settings more likely to be school ready than those being looked after by parents (OR = 1.76, p=0.02)

For peer review only

The Stata Do file for all analyses is available at: https://www.dropbox.com/s/zxsl4cl87imydp0/SchoolreadinessPRM.do?dl=0

SUPPLEMENTARY FILE 2

Table 1 - Adjusted associations for the predictor variables in model 1 (13 predictors) using multiple imputed data (n=11,879)

Predictor	Adjusted OR (95% CI)	Weighting (rank)
**GROUP 1 - DEMOGRAPHIC & INDIVIDUAL FACTORS**		
**Gender**		8.5 (5)
Female	1	
Male	1.86 (1.62,2.14)	
**Ethnicity**		15.7 (3)
White	1	
Mixed	1.04 (0.62,1.75)	
Indian	2.68 (1.85,3.89)	
Pakistani and Bangladeshi	3.85 (2.94,5.04)	
Black or Black British	2.31 (1.43,3.72)	
Other ethnic group	3.95 (2.30,6.77)	
**Mother's age at birth of first child**		1.5 (12)
30-39	1	
40+	1.05 (0.67,1.64)	
20-29	1.22 (0.99,1.51)	
14-19	1.22 (0.93,1.59)	
**Birth weight (<2500grams)**		1.2 (13)
Normal/high	1	
Low birthweight	1.52 (1.18,1.97)	
**Maternal Mental Health (Diagnosed depression/anxiety)**		1.5 (11)
No	1	
Yes	1.15 (0.98,1.34)	
**Child developmental milestones**		
Developmental score	1.10 (1.07,1.13)	2.8 (10)
**GROUP 2 - LIFESTYLE FACTORS**		
**Duration of breastfeeding**		3.6 (9)
6 months or more	1	
6 weeks - 6 months	1.17 (0.92,1.48)	
One week or less	1.15 (0.90,1.48)	
1 - 6 weeks	1.22 (0.96,1.57)	
Never	1.58 (1.29,1.95)	
**GROUP 3 - SOCIAL & COMMUNITY NETWORKS**		
**Number of children in family**		7.1 (6)
One child	1	
Two or three children	1.40 (1.19,1.63)	
Four or more children	2.48 (1.94,3.16)	
**GROUP 4 - LIVING & WORKING CONDITIONS**		
**Maternal education**		16.7 (2)
Degree plus	1	

For peer review only - http://bmjopen.bmj.com/site/about/guidelines.xhtml

Diploma	0.88 (0.61,1.26)	
A levels	1.13 (0.80,1.59)	
GCSE A-C	1.34 (1.01,1.78)	
GCSE D-G	1.72 (1.23,2.39)	
None	1.74 (1.28,2.38)	
**Workforce status**		
Both parents in work	1	
One parent in work	0.94 (0.78,1.12)	6.5 (7)
Neither parent in work	1.21 (0.93,1.57)	
**Housing tenure**		
Owner occupied	1	
Private rented	1.18 (0.90,1.54)	5.5 (8)
Social housing	1.43 (1.18,1.72)	
Other	0.96 (0.69,1.35)	
**GROUP 5 - SOCIOECONOMIC AND WIDER FACTORS**		
**Social class**		
Managerial & professional	1	
Intermediate	0.98 (0.75,1.29)	
Small employers & own account	1.32 (0.87,2.00)	17.6 (1)
Lower supervisory & technical	1.50 (1.06,2.13)	
Semi-routine & routine	1.77 (1.38,2.27)	
Never worked & long-term unemployed	2.19 (1.53,3.15)	
**Annual income**		
£33,000+	1	
£22,000-£33,000	1.33 (1.02,1.72)	11.9 (4)
£11,000-£22,000	1.67 (1.30,2.14)	
£0-£11,000	2.14 (1.60,2.87)	
**ROC Analysis**	**AUROC = 0.79 (95% CI 0.78,0.80)**	

For peer review only

SUPPLEMENTARY FILE 3

Integrated discrimination improvement (IDI) analysis was run using Stata function 'idi', which compares the discrimination ability between two logistic regression prediction models. In the first stage of this analysis, the IDI of a PRM with just the strongest predictor variable (social class) was compared to a model with all 13 predictors. Adding the additional 12 predictors lead to a 7.3% increase in IDI. In each subsequent analysis, an additional predictor variable was added according to the ranking of variables from the dominance analysis (Table 1).

Predictor	Weighting	Rank
Social Class	17.38	1
Ethnic group	14.66	2
Maternal education	13.55	3
Income band	12	4
Gender	9.54	5
Number of children	7.84	6
Parent's employment	6.9	7
Housing type	5.65	8
Child development	3.9	9
Breastfeeding	3.9	10
Mother's age at birth of first child	2.87	11
Low birth weight	1.42	12
Mental health	0.38	13

Table 1 - Results of the dominance analysis for model 1

The full results of integrated discrimination improvement (IDI) analysis are shown in Table 2.

Variables included	IDI (%)	p	1-IDI
1	7.3%	<0.00001	92.7%
2	5.3%	<0.00001	94.7%
3	3.8%	<0.00001	96.2%
4	3.5%	<0.00001	96.5%
5	2.3%	<0.00001	97.7%
6	1.3%	<0.00001	98.7%
7	1.0%	<0.00001	99.0%
8	0.9%	<0.00001	99.1%
9	0.6%	0.00001	99.4%
10	0.6%	0.00001	99.4%
11	0.2%	0.01402	99.8%
12	0.0%	0.52356	100.0%

Table 2 - Results of integrated discrimination improvement analysis for 12 models

A 6-predictor model was chosen as this offered the optimal balance between parsimony and discrimination.

For peer review only

SUPPLEMENTARY FILE 4

Table 1 - Adjusted associations for the predictor variables in model 2 (6 predictors) using complete cases (n=11,146) and multiple imputed data (n=11,879). The weightings and rank are from dominance analysis of the complete case sample.

Predictors	Adjusted OR (95% CI) - complete case	Adjusted OR (95% CI) - multiple imputation	Weighting (rank)
**GROUP 1 - DEMOGRAPHIC & INDIVIDUAL FACTORS**			
**Gender**			
Female	1	1	9.9 (5)
Male	1.99 (1.72,2.31)	1.93 (1.68,2.22)	
**Ethnicity**			
White	1	1	13.7 (4)
Mixed	1.2 (0.77,1.88)	1.26 (0.83,1.90)	
Indian	1.64 (1.09,2.47)	1.72 (1.14,2.59)	
Pakistani and Bangladeshi	2.67 (2.10,3.41)	2.71 (2.11,3.47)	
Black or Black British	2.32 (1.52,3.54)	2.69 (1.80,4.02)	
Other ethnic group	1.98 (1.10,3.58)	2.06 (1.27,3.32)	
**GROUP 3 - SOCIAL & COMMUNITY NETWORKS**			
**Number of children in family**			
One child	1	1	9.5 (6)
Two or three children	1.48 (1.27,1.73)	1.45 (1.25,1.69)	
Four or more children	2.89 (2.23,3.75)	2.62 (2.03,3.38)	
**GROUP 4 - LIVING & WORKING CONDITIONS**			
**Maternal education**			
Degree plus	1	1	20.5 (3)
Diploma	0.87 (0.58,1.29)	0.88 (0.60,1.28)	
A levels	1.05 (0.72,1.53)	1.06 (0.74,1.52)	
GCSE A-C	1.43 (1.02,1.99)	1.55 (1.14,2.12)	
GCSE D-G	1.78 (1.23,2.58)	2.14 (1.51,3.03)	
None	2.01 (1.44,2.81)	2.42 (1.77,3.30)	
**GROUP 5 - SOCIOECONOMIC AND WIDER FACTORS**			
**Social class**			
Managerial & professional	1	1	26.0 (1)
Intermediate	1.17 (0.88,1.55)	1.14 (0.86,1.51)	
Small employers & own account	1.44 (0.91,2.28)	1.52 (0.99,2.33)	
Lower supervisory & technical	2.01 (1.42,2.86)	1.92 (1.37,2.68)	
Semi-routine & routine	2.41 (1.86,3.12)	2.16 (1.68,2.78)	
Never worked & long-term unemployed	3.34 (2.41,4.63)	2.95 (2.14,4.07)	
**Annual income**			
£33,000+	1	1	20.6 (2)
£22,000-£33,000	1.33 (0.97,1.81)	2.65 (2.01,3.50)	
£11,000-£22,000	1.88 (1.42,2.50)	1.75 (1.32,2.31)	
£0-£11,000	2.98 (2.26,3.92)	1.29 (0.95,1.75)	

For peer review only

**ROC Analysis**	**AUROC = 0.78** (95% CI 0.77 - 0.79) n=11,146	**AUROC = 0.78** (95% CI 0.77 - 0.79) n=11,879

For peer review only

TRIPOD Checklist: Prediction Model Development

Section/Topic	Item	Checklist Item	Page
**Title and abstract**			
Title	1	Identify the study as developing and/or validating a multivariable prediction model, the target population, and the outcome to be predicted.	1
Abstract	2	Provide a summary of objectives, study design, setting, participants, sample size, predictors, outcome, statistical analysis, results, and conclusions.	2
**Introduction**			
Background and objectives	3a	Explain the medical context (including whether diagnostic or prognostic) and rationale for developing or validating the multivariable prediction model, including references to existing models.	3
	3b	Specify the objectives, including whether the study describes the development or validation of the model or both.	3
**Methods**			
Source of data	4a	Describe the study design or source of data (e.g., randomized trial, cohort, or registry data), separately for the development and validation data sets, if applicable.	3
	4b	Specify the key study dates, including start of accrual; end of accrual; and, if applicable, end of follow-up.	3
Participants	5a	Specify key elements of the study setting (e.g., primary care, secondary care, general population) including number and location of centres.	3-4
	5b	Describe eligibility criteria for participants.	4
	5c	Give details of treatments received, if relevant.	
Outcome	6a	Clearly define the outcome that is predicted by the prediction model, including how and when assessed.	4
	6b	Report any actions to blind assessment of the outcome to be predicted.	
Predictors	7a	Clearly define all predictors used in developing or validating the multivariable prediction model, including how and when they were measured.	4-5
	7b	Report any actions to blind assessment of predictors for the outcome and other predictors.	temporal ordering in cohort
Sample size	8	Explain how the study size was arrived at.	5
Missing data	9	Describe how missing data were handled (e.g., complete-case analysis, single imputation, multiple imputation) with details of any imputation method.	6
Statistical analysis methods	10a	Describe how predictors were handled in the analyses.	5-6
	10b	Specify type of model, all model-building procedures (including any predictor selection), and method for internal validation.	6
	10d	Specify all measures used to assess model performance and, if relevant, to compare multiple models.	6
Risk groups	11	Provide details on how risk groups were created, if done.	
**Results**			
Participants	13a	Describe the flow of participants through the study, including the number of participants with and without the outcome and, if applicable, a summary of the follow-up time. A diagram may be helpful.	6
	13b	Describe the characteristics of the participants (basic demographics, clinical features, available predictors), including the number of participants with missing data for predictors and outcome.	6-7
Model development	14a	Specify the number of participants and outcome events in each analysis.	10
	14b	If done, report the unadjusted association between each candidate predictor and outcome.	8-10
Model specification	15a	Present the full prediction model to allow predictions for individuals (i.e., all regression coefficients, and model intercept or baseline survival at a given time point).	
	15b	Explain how to the use the prediction model.	
Model performance	16	Report performance measures (with CIs) for the prediction model.	10
**Discussion**			
Limitations	18	Discuss any limitations of the study (such as nonrepresentative sample, few events per predictor, missing data).	11-12
Interpretation	19b	Give an overall interpretation of the results, considering objectives, limitations, and results from similar studies, and other relevant evidence.	11
Implications	20	Discuss the potential clinical use of the model and implications for future research.	11-13
**Other information**			
Supplementary information	21	Provide information about the availability of supplementary resources, such as study protocol, Web calculator, and data sets.	
Funding	22	Give the source of funding and the role of the funders for the present study.	

We recommend using the TRIPOD Checklist in conjunction with the TRIPOD Explanation and Elaboration document.
